# Molecular Phylogeny of Sequenced *Saccharomycetes* Reveals Polyphyly of the Alternative Yeast Codon Usage

**DOI:** 10.1093/gbe/evu152

**Published:** 2014-07-22

**Authors:** Stefanie Mühlhausen, Martin Kollmar

**Affiliations:** Group Systems Biology of Motor Proteins, Department of NMR-Based Structural Biology, Max-Planck-Institute for Biophysical Chemistry, Göttingen, Germany

**Keywords:** genetic code, codon reassignment, codon usage, evolution, *Candida*

## Abstract

The universal genetic code defines the translation of nucleotide triplets, called codons, into amino acids. In many Saccharomycetes a unique alteration of this code affects the translation of the CUG codon, which is normally translated as leucine. Most of the species encoding CUG alternatively as serine belong to the *Candida* genus and were grouped into a so-called CTG clade. However, the “*Candida* genus” is not a monophyletic group and several *Candida* species are known to use the standard CUG translation. The codon identity could have been changed in a single branch, the ancestor of the *Candida*, or to several branches independently leading to a polyphyletic alternative yeast codon usage (AYCU). In order to resolve the monophyly or polyphyly of the AYCU, we performed a phylogenomics analysis of 26 motor and cytoskeletal proteins from 60 sequenced yeast species. By investigating the CUG codon positions with respect to sequence conservation at the respective alignment positions, we were able to unambiguously assign the standard code or AYCU. Quantitative analysis of the highly conserved leucine and serine alignment positions showed that 61.1% and 17% of the CUG codons coding for leucine and serine, respectively, are at highly conserved positions, whereas only 0.6% and 2.3% of the CUG codons, respectively, are at positions conserved in the respective other amino acid. Plotting the codon usage onto the phylogenetic tree revealed the polyphyly of the AYCU with *Pachysolen tannophilus* and the CTG clade branching independently within a time span of 30–100 Ma.

## Introduction

The standard genetic code has been altered in many organisms. In eukaryotes, natural alterations have been identified in mitochondria, in which the universal stop codon UGA is translated as tryptophane (Yokobori et al. 2001; Watanabe and Yokobori 2011), in ciliates, in which the function of the stop codons UAA and UGA was changed to code for glutamine (Tourancheau et al. 1995), and in some *Candida* yeasts (Ohama et al. 1993; Sugita and Nakase 1999; Santos et al. 2011), in which the leucine codon CUG translation was changed to serine. In taxonomic terms, the definition of the genus *Candida* is not very specific. *Candida* species comprise a group of about 850 organisms (Robert et al. 2013), which can be distantly related as, for example, *Candida glabrata*, *Ca. albicans*, and *Ca. caseinolytica*. Although it has been claimed that almost all *Candida* species, with the exceptions of *Ca. glabrata* and *Ca. krusei*, belong to a single clade (Butler et al. 2009), which is commonly referred to as “*Candida* clade,” many analyses showed that *Candida* species are spread over the entire *Saccharomycetes* clade (Diezmann et al. 2004; Kurtzman and Suzuki 2010; Kurtzman et al. 2011; Kurtzman 2011). In addition, different names have been given to the same yeast species in the past depending on the reproductive stage, in which they had been identified. For example, the names *Meyerozyma guilliermondii* (teleomorph), *Ca. guilliermondii* (anamorph), *Yamadazyma guilliermondii*, and *Pichia guilliermondii* are used for the same holomorph (whole fungus, including anamorph and teleomorph).

Because *Ca. albicans* is the most prominent representative of the genus *Candida* and was the first shown to use the alternative CUG encoding, the term “*Candida* clade” is often used synonymous to “CTG clade” and most studies about CUG encoding concentrate on *Ca. albicans* and closely related *Candida* species. Therefore, depending on which node is used for defining the “*Candida* clade,” several species of the branch containing *Ca. albicans* might decode CUG as leucine and many species outside the “*Candida* clade” are also named *Candida*. Similarly, there are many non-*Candida* species decoding CUG as serine. In order to resolve this ambiguity, one of the major questions is whether the change of identity of the CUG codon happened to a single branch or to several branches independently. The latter scenario would be in agreement with the proposed timing of the code alteration (∼270 Ma) and the split of the *Saccharomyces* and *Candida* clades about 170 Ma (Massey et al. 2003; Santos et al. 2011) implying that the CUG codon was highly ambiguous for approximately 100 Myr. Species that branched within this time span could have adopted either of the two decoding possibilities. Most phylogenetic analyses of yeast species used 28s rRNA, 18s rRNA, actin and translation elongation factor-1α for reconstructing single or combined trees, which is a very successful approach when analyzing hundreds of species (Kurtzman 2011). However, higher accuracy for tree topologies is obtained in phylogenomic studies that use dozens to thousands of concatenated homologs in tree computations (Fitzpatrick et al. 2006). For the latter approach, it is important to distinguish orthologs from paralogs. Only a few yeast species have been included in phylogenomic studies so far.

In addition to assigning the CUG codon usage by phylogenetic comparisons, it has been suggested to base the translation of codon CUG on the presence of Q9 as the major ubiquinone (Sugita and Nakase 1999). Ubiquinone (Coenzyme Q, CoQ) is an electron carrier in the respiratory chain and involved in oxidative stress resistance (Szkopińska 2000; Turunen et al. 2004). Most enzymes of the ubiquinone biosynthesis pathway are conserved between *Saccharomyces cerevisiae* and *Ca. albicans*. However, the *Ca. albicans* ubiquinone has a nonaprenyl side chain (Q9) whereas the budding yeast has six isoprene units (Q6). Although a great variety of CoQ types are present in the genus *Candida* (Schauer and Hanschke 1999), the species analyzed at that time, which were known to decode CUG as serine, lacked galactose in the cells and possessed Q9, with the exception of *Clavispora lusitaniae* (Q8). Based on these ideas, an extensive study revealed 89 yeast strains to possess coenzyme Q9 (except for *Cl. lusitaniae*) and to miss galactose in the cell (Sugita and Nakase 1999). However, in vitro translation assays showed 11 of these species to use codon CUG as leucine. The incongruence in many instances of the presence of Q9, absence of galactose, and molecular phylogenetic data has also been reported by others (Kurtzman and Robnett 1997; Diezmann et al. 2004; Kurtzman and Suzuki 2010).

Sixty yeast species (not counting strains and data under embargo) have already been sequenced and assembled (http://www.diark.org, last accessed May 20, 2014). Only half of these genomes, which include both yeasts of the “CTG clade” and of the *Saccharomyces* clade, have been analyzed in detail yet. In addition, sequencing efforts increasingly focus on genomes of rarely studied species, whose phylogenetic relationships are not clear. For most of these genomes, gene predictions were even done and made available using both the Standard Code and the Alternative Yeast Nuclear Code resulting in mixed data sets. In order to determine whether there was a time span with a CUG codon usage ambiguity or whether the codon usage alteration could be attributed to a single event, we performed a phylogenomic analysis of highly validated protein sequences of 26 members of the actin, actin-related, tubulin, myosin, kinesin, dynein, and actin-capping protein families of the sequenced yeast species. CUG codon translation has been assigned by analyzing the respective amino acids of the sequences in the context of the sequence alignments.

## Materials and Methods

### Identification and Annotation of the Proteins

Some fungal actin-related proteins and myosins have been extracted from previously published data sets (Odronitz and Kollmar 2007; Hammesfahr and Kollmar 2012). The sequences were updated based on newer genome assemblies if necessary and corrected for CUG usage. The data for the other proteins and the species not included in the published data sets have essentially been obtained as described (Odronitz and Kollmar 2007). Shortly, the corresponding gene regions have been identified in TBLASTN searches starting with the respective protein sequences of homologs of *Sa. cerevisiae*. The respective genomic regions were submitted to AUGUSTUS (Stanke and Morgenstern 2005) to obtain gene predictions. However, feature sets are only available for a few species of the *Saccharomycetes* clade. Therefore, all hits were subsequently manually analyzed at the genomic DNA level. When necessary, gene predictions were corrected by comparison with the homologs already included in the multiple sequence alignments. Especially, the short exons at the 5′-ends of the tubulin and actin genes were not always identified in automatic gene predictions. Expressed sequence tag data to help in the annotation are only available for a few species in public databases.

In the last years, genome-sequencing efforts have been extended from sequencing species from new branches to sequencing closely related organisms. Here, these species include, for example, *Saccharomyces* and *Eremothecium* species. Protein sequences from these closely related species have been obtained by using the cross-species functionality of WebScipio (Odronitz et al. 2008; Hatje et al. 2011). Nevertheless, for all these genomes TBLASTN searches have been performed. With this strategy, we sought to ensure that we would not miss more divergent protein family homologs, which might have been derived by species-specific inventions or duplications. However, the *Saccharomycetes* belong to the fast-evolving species and therefore these cross-species gene reconstructions only worked for a few species.

All sequence-related data (protein names, corresponding species, sequences, and gene structure reconstructions) and references to genome sequencing centers are available at CyMoBase (http://www.cymobase.org; Odronitz and Kollmar 2006). A list of the analyzed species, their abbreviations as used in the alignments and trees, as well as references and accession numbers is also available as supplementary file S1, Supplementary Material online. WebScipio (Odronitz et al. 2008; Hatje et al. 2011) was used to reconstruct the gene structure of each sequence. Throughout this study we use the teleomorph names of all species.

### Generating the Multiple Sequence Alignment

The protein sequences were added to the already existing multiple sequence alignments (Odronitz and Kollmar 2007; Hammesfahr and Kollmar 2012). In detail, we first aligned every newly predicted sequence to its supposed closest relative using ClustalW (Thompson et al. 2002) and added it then to the multiple sequence alignment. During the subsequent sequence validation process, we manually adjusted the obtained alignment by removing wrongly predicted sequence regions and filling gaps. Still, in those sequences derived from low-coverage genomes many gaps remained. To maintain the integrity of exons preceded or followed by gaps, gaps reflecting missing parts of the genomes were added to the multiple sequence alignment. Sequence homologs of the same protein family (e.g., α-tubulin, β-tubulin, and γ-tubulin) were kept in one single protein family alignment. The actin/actin-related protein alignment therefore contains 11 subfamilies, the CapZ alignment 2, the tubulin alignment 3, the myosin alignment 3, and the kinesin alignment 6. Only single dynein heavy chain homologs have been found in the analyzed species. The sequence alignments can be obtained from CyMoBase.

### Computing Sequence Conservation

The residue conservation at alignment positions was calculated with the conservation code toolbox as implemented by Capra and Singh (2007). Conservation was estimated with the property-entropy method, an entropy measurement refined with respect to chemical properties of amino acids. Scores were calculated with conservation of adjacent amino acids incorporated (window size 3) and not (window size 0). Except for window size and scoring method, standard parameters were used. Unexpectedly, although window sizes were applied, the rest of the alignment still seemed to have an influence, albeit marginal, on the conservation scores. In addition, amino acids given as “X” are replaced by hyphens “-” by the software, which denote gap positions in the alignment. Gap positions, however, indicate a biological relevant deletion of this position in the sequence and thus have a strong influence on the conservation score. Therefore, conservation estimates were performed on small sections of the concatenated alignment including *Schizosaccharomyectes* sequences. For each serine and leucine position, the alignment was reduced to this position and 15 adjacent positions in each direction. Sequences with CUG codons at the respective leucine or serine position were removed from the alignment sections.

### Computing and Visualizing Phylogenetic Trees

For calculating phylogenetic trees, the alignments of the CapZ, actin and actin-related, tubulin, kinesin, and the myosin protein families were split into subfamily alignments and the respective alignments were concatenated (supplementary file S2, Supplementary Material online). As outgroup, sequences from *Schizosaccharomycetes* (*Sc. pombe*, *Sc. octosporus*, *Sc. japonicus*, and *Sc. cryophilus*) were taken. Gblocks v.0.91b (Talavera and Castresana 2007) was run with standard parameters, and half gap positions allowed to reduce the concatenated alignment from 28,202 amino acids to 9,788 amino acids in 230 blocks. The phylogenetic trees were generated using four different methods: Neighbor-Joining (NJ), maximum-likelihood (ML), Bayesian inference, and split networks: 1) ClustalW v.2.0.10 (Thompson et al. 2002) was used to calculate unrooted trees with the NJ method. For each data set, bootstrapping with 1,000 replicates was performed. 2) ML analysis with estimated proportion of invariable sites and bootstrapping (1,000 replicates) was performed using RAxML v.7.3.1 (Stamatakis et al. 2008). To this end, ProtTest v.3.2 was used first to determine the most appropriate of the available 112 amino acid substitution models (Darriba et al. 2011). Within ProtTest, the tree topology was calculated with the BioNJ algorithm and both the branch lengths and the model of protein evolution were optimized simultaneously. The Akaike Information Criterion with a modification to control for small sample size (AICc, with alignment length representing sample size) identified the WAG model with gamma model of rate heterogeneity and site-specific evolutionary rates to be the best. 3) The ML analysis was repeated on an additional data set generated from the concatenated alignment. In this data set, amino acids encoded by CUG codons were substituted by “X.” Gblocks was invoked as described above, with standard parameters and half gap positions allowed, to reduce the alignment to 8,903 positions in 297 blocks. The phylogenetic tree was generated with RAxML under the WAG + Γ + F model and 1,000 replicates were performed. 4) Posterior probabilities were generated using MrBayes v3.2.1 (Ronquist and Huelsenbeck 2003). Using the mixed amino acid option, two independent runs with 100,000 generations, four chains, and a random starting tree were performed. MrBayes used the WAG model for all protein alignments. The trees were calculated based on a reduced data set generated by Gblocks using standard parameters allowing no gap positions. This data set includes 5,038 positions in 131 blocks. Trees were sampled every 1,000th generation and the first 25% of the trees were discarded as “burn-in” before generating a consensus tree. 5) An unrooted phylogenetic split network was generated with SplitsTree v. 4.1.3.1 (Huson and Bryant 2006). The Neighbor-Net method as implemented in SplitsTree was used to identify alternative splits. Phylogenetic trees and networks were visualized with FigTree v.1.3.1 (Rambaut and Drummond 2009) and SplitsTree v.4.13.1, respectively, and are available as supplementary figure S1, Supplementary Material online.

### Estimating the Divergence Times

The divergence times of species using alternative yeast codon usage (AYCU) were estimated with a penalized-likelihood approach as implemented in treePL (Smith and O’Meara 2012). The underlying tree topology was generated with RAxML under the WAG + Γ + F model on the alignment with CUG codons assigned. The splits between *Sa. cerevisiae* and *Ca. albicans*, and *Sa. cerevisiae* and *Sc. pombe* (Heckman et al. 2001; Douzery et al. 2004) were constrained both individually and combined.

## Results

### Phylogeny of Sequenced Yeast Species

**Fig. 1.—:**
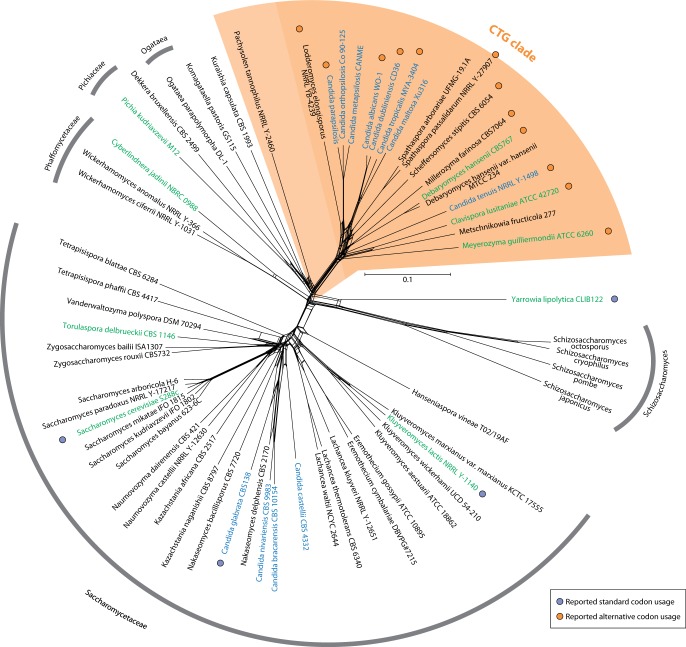
Phylogenetic relationship between Saccharomycetes. The unrooted phylogenetic network was generated using the Neighbor-Net method as implemented in SplitsTree 4.1.3.1. The *Schizosaccharomycetes* species were included as outgroup. The network strongly supports the *Saccharomycetaceae* and the CTG clade (highlighted in orange). The grouping of *Pachysolen tannophilus* is not unambiguously resolved. Species of the genus *Candida* are highlighted in blue (teleomorph names) and green (if anamorphs of the species are called *Candida*) showing the paraphyly (or misassignment) of this genus. Orange and purple dots mark species, for which alternative or standard codon usage has already been shown elsewhere.

In order to obtain a reliable phylogeny of the sequenced yeast species, we choose a small-scale phylogenomics approach. The more data (i.e., orthologs) included in the alignment the better and more robust the phylogenetic trees should become. Another important parameter for the reliability of the computed trees is the quality of the underlying sequence data. Here, we manually determined 26 motor and cytoskeletal proteins for each of the 60 sequenced yeast species. These 26 proteins belong to six major protein families, the actin/actin-related proteins, the tubulins, the myosins, the kinesins, the dynein heavy chain proteins, and the actin capping proteins of the CapZ complex. We included all proteins of these six families in the analysis except some kinesins that are unique to certain species. Many of the actin and tubulin genes contain very short exons of one to three residues that are not included in data sets of automatic gene predictions. By manually inspecting the genomic DNA sequences we could completely reconstruct all genes as long as the genomic sequences did not contain gaps. The concatenated sequences consist of on average 19,300 residues per species amounting to 28,202 alignment positions. Gblocks (Talavera and Castresana 2007) was used with less stringent and more stringent parameters to reduce the alignment to 9,788 and 5,038 positions, respectively. NJ and ML methods were used to construct trees from the extended alignment, and the Bayesian approach to compute a tree from the shorter alignment (supplementary fig. S1, Supplementary Material online). To evaluate the influence of misassigned CUG codons on the tree topology, all CUG codons were translated with “X” and the tree reconstruction repeated. The topology of this unbiased tree is identical to that based on the alignment with assigned CUG codons. As outgroup, we included four *Schizosaccharomyces* species. The constructed trees have almost the same topology with the major exceptions being the placing of Kluyveromyces in the NJ tree, in which they group to *Lachancea* and *Eremothecium*. To highlight alternative phylogenetic relationships between the species, we also constructed a phylogenetic network using the Neighbor-Net method (fig. 1). In agreement with other studies (Kurtzman and Robnett 2013a, 2013b), the trees show that *Yarrowia lipolytica* is the earliest diverging *Saccharomycetes*. The Saccharomycetaceae form a highly supported group together with the early separating Pfaffomycetaceae (supplementary fig. S1, Supplementary Material online). In our analysis, *Pi. kudriavzevii* and *Ogataea parapolymorpha* branch together with *Dekkera bruxellensis*, *Kuraishia capsulata*, and *Komagataella pastoris*. This branch is more closely related to the *Ca. albicans* species group than to the Saccharomycetaceae (supplementary fig. S1, Supplementary Material online), but the species encode CUG as leucine and not as serine. The species, for which the AYCU has been determined previously (e.g., *Ca. albicans*, *Ca. dubliniensis* and *Ca. parapsilosis*), are part of a highly supported group in all phylogenetic trees (fig. 1 and supplementary fig. S1, Supplementary Material online). Therefore, we refer to this group from here on as CTG clade. The CTG clade consists of several “*Candida*” species including *Ca. albicans* and *Ca. tenuis*, and also many others such as species from Metschnikowiaceae and Debaryomycetaceae. In all trees, *Pachysolen* groups outside the CTG clade as sister to the *Pichiaceae/Ogataea* (supplementary fig. S1, Supplementary Material online). In the phylogenetic network, *Pachysolen* is placed at the origin of the CTG clade but there are many connections supporting alternative topologies. Interestingly, species of the *Candida* genus are spread all over the tree grouping to the Saccharomycetes and to various subgroups of the CTG clade (fig. 1).

### Conservation of Amino Acids at CUG Codon Positions in Class I Myosins

**Fig. 2.—:**
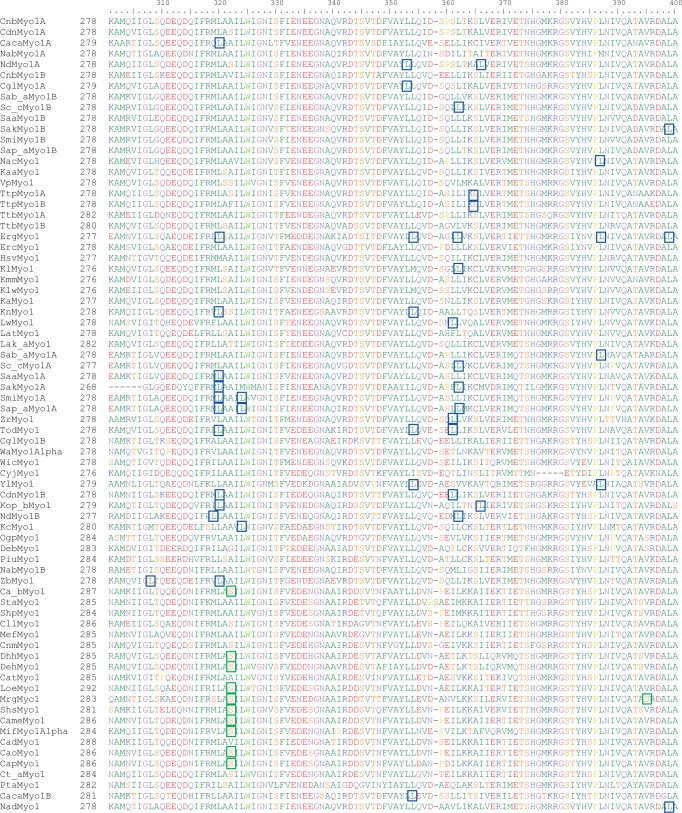
Sequence alignment of the yeast class I myosins highlighting leucines and serines encoded by CUG. The protein sequence alignment represents part of the class I myosin alignment (for the complete alignment, see supplementary fig. S2, Supplementary Material online). Numbers on the left denote the residue numbers of the first amino acids of the sequences in this section of the alignment. All CUG positions occurring in the aligned class I myosin genes are highlighted. We assigned the most probable translation scheme to each species and translated the CUG codons accordingly. Blue and green boxes indicate CUG codons coding for leucine and serine, respectively.

In order to determine whether CUG codons are coding for conserved amino acids (leucine or serine), we performed a thorough analysis of the alignments of all protein subfamilies. As representative example, a part of the alignment of the class I myosins is presented in figure 2 (the full class I myosin alignment is available in supplementary fig. S2, Supplementary Material online). This analysis provided several surprising findings: 1) The class I myosin alignment contains 33 positions with 100% leucine conservation but only 11 positions that show 100% serine conservation; 2) except for *Wickerhamomyces anomalus* and *Ca. tenuis* (no CUG codons in their class I myosin genes), and *Cl. lusitaniae*, *Ca. tropicalis*, and *Pachysolen* (two to three CUG codons in their class I myosin genes but not at conserved positions), all *Saccharomycetaceae*, *Pfaffomycetaceae*, *Pichiaceae*, and *Ogataea* species, and *Dek. bruxellensis*, *Ku. capsulata*, *Ko. pastoris*, and *Yar. lipolytica*, have at least one CUG codon at one of the 100% conserved leucine positions; and 3) except for the sequences of the *Debaryomyces* and *Spathaspora* species investigated here, whose CUG positions are in almost all cases identical, none of the other closely related species (e.g., *Saccharomyces* or *Pichiaceae*) has conserved CUG positions (fig. 2). This is especially apparent for the six analyzed *Saccharomyces* species that have duplicated class I myosins but do not have even a single conserved CUG position between the two paralogs. In addition, CUG positions are not conserved within any of the orthologs. There is one position in the alignment (position 322), at which ten of the species of the CTG clade have a CUG-encoded serine whereas serines, alanines, and glycines are found at that position in the other species (fig. 2).

### Conservation of Amino Acids at CUG Codon Positions in General

**Fig. 3.—:**
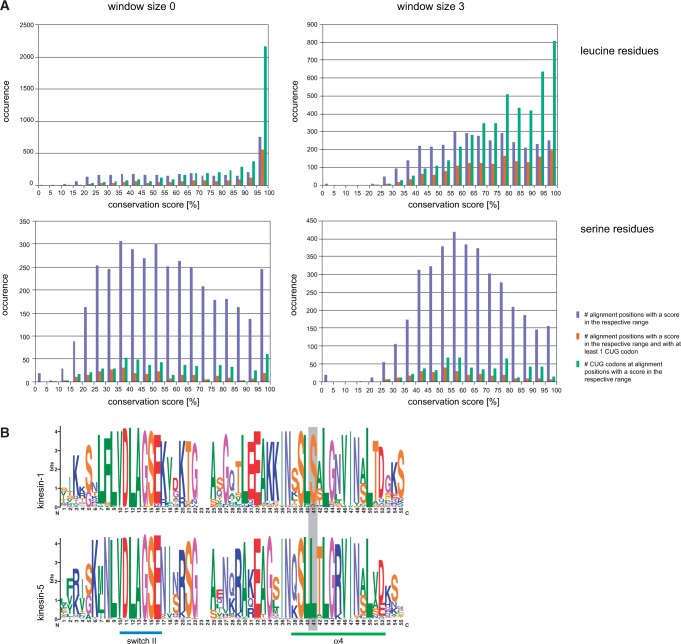
Conservation of serine and leucine positions. (*A*) The charts show the amino acid conservation at all alignment positions of the Gblocks reduced concatenated alignment of the 26 cytoskeletal and motor proteins, at which at least one leucine (upper charts) or one serine (lower charts) is present. The sequence conservation has been determined based on the property-entropy divergence, as described in Capra and Singh (2007). With a window size of 0, each column is scored independently (left row), whereas the surrounding three columns are also taken into account with a window size of 3 (right row). Blue bars represent the number of alignment positions with a conservation score for leucine and serine residues, respectively, within the given half-bounded intervals. Red bars denote the number of alignment positions with respect to conservation, at which at least one CUG codon is present independent of its translation. Green bars give the total numbers of CUG codons at the respective alignment positions. (*B*) The weblogos (Crooks et al. 2004) show the sequence conservation of two kinesin subfamilies, kinesin-1 and kinesin-5, within the family-defining motor domain around the highly conserved switch II and α-helix α4 motifs. At the position within α4 marked by a grey bar, kinesin-1 sequences contain a highly conserved serine whereas kinesin-5 sequences contain a highly conserved leucine indicating the need to resolve subfamily relationships when determining CUG codon usage by sequence conservation.

The analysis of the complete data with respect to amino acid similarity at alignment positions, at which at least one leucine/serine is present, showed that positions with leucines are stronger conserved than positions with serines although in total serines have been observed at as many alignment positions as leucines (fig. 3*A* and supplementary fig. S3, Supplementary Material online). We determined the conservation of each position alone (window size = 0) and within its closest environment (window size = 3). The analysis of CUG codons present at leucine positions showed that CUG codons are not evenly distributed to all positions with leucines but enriched at highly conserved alignment positions (fig. 3*A*). In contrast, there are far less CUG codons at serine positions, and these are in general present at less conserved positions compared with the leucine encoding CUG codons. However, although small there are a considerable number of CUG codons at highly conserved and thus discriminative serine positions (fig. 3*A*). In order to determine the amino acid conservation, it is extremely important to resolve protein subfamily relationships. For example, the kinesin-1 subfamily members have a highly conserved serine at the same position where the kinesin-5 proteins have a highly conserved leucine (fig. 3*B*).

### Assignment of the Codon Usage Scheme by Amino Acid Conservation Patterns

**Fig. 4.—:**
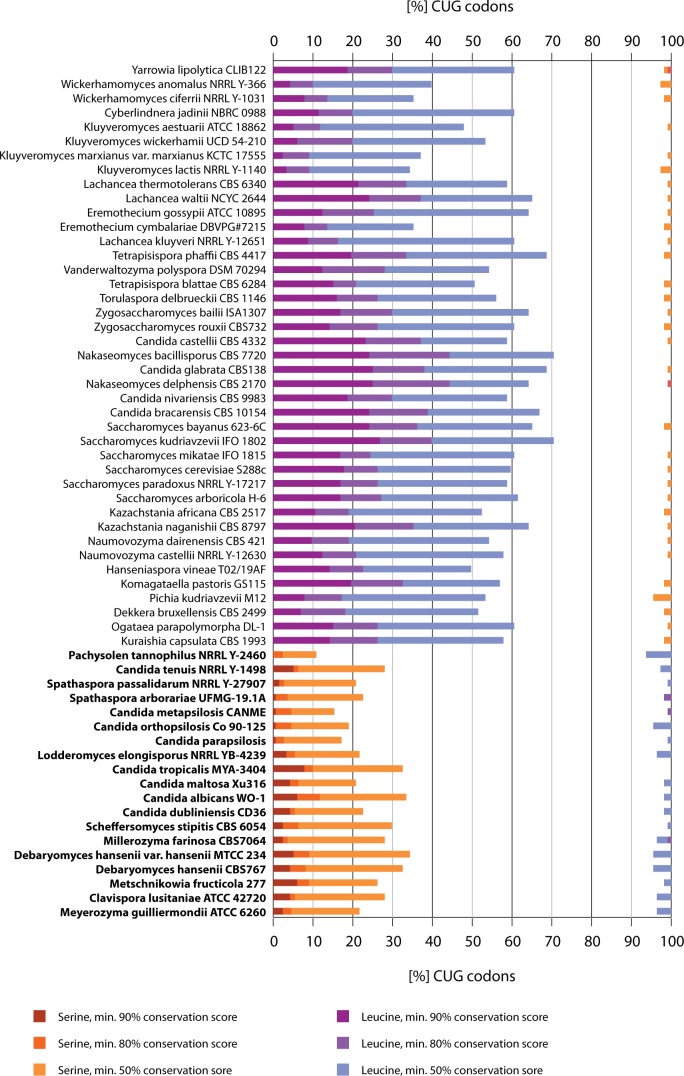
CUG codon usage at conserved leucine and serine positions. The graph presents the CUG codon usage with respect to alignment position conservation. For each species we determined the percentage of CUG codons at alignment positions with conservation scores of ≥90%, ≥80%, and ≥50% and a window size of 3, respectively, in the concatenated alignment of the 26 motor and cytoskeletal proteins. Red and blue colors denote the percentages of CUG codons present at alignment positions enriched in serines and leucines, respectively. For comparison, we plotted the percentages of CUG codons at positions of the assigned codon translation to the left (% CUG codons at leucine positions for species using the standard code and % CUG codons at serine positions for species using the AYCU) and the percentages of CUG codons present at alignment positions enriched in the respective other amino acid to the right. When considering only highly conserved alignment positions (≥90% conserved) the CUG codon translation assignment is unambiguous. Species using the AYCU are highlighted in bold.

In order to determine the CUG codon translation, we investigated whether the CUG codons within the analyzed sequences of each species are at highly conserved leucine or serine positions in the alignment. Therefore, we first determined the percentage of CUG codons at extremely conserved leucine and serine positions (conservation score of ≥90%; fig. 4). The analysis resulted in three groups of species: The first group, including *Sa. cerevisiae*, had 2.8–26.4% (on average 14.3%) of the CUG codons at highly conserved leucine positions. The second group, including *Ca. albicans*, had CUG codons at conserved serine positions although the total numbers were considerably lower than those for the leucine positions (0.9–7.5%; on average 3.4%), *Pachysolen tannophilus* did not have any CUG codons at either conserved position. A similar situation was found when analyzing the CUG codon distribution at a conservation score of more than or equal to 80% (fig. 4). In the same group of species as at the conservation score of more than or equal to 90%, 8.4–44.4% (on average 24.5%) of the CUG codons are at leucine positions. At that conservation level, one of the CUG codons of *Yarrowia* is found at a conserved serine position. At the same conservation score (≥80%), all other species have CUG codons at highly conserved serine positions (2.2–11.8%; on average 6.1%). In five species, one to two of the CUG codons are at conserved leucine positions (*Ca. metapsilosis, Ca. tenuis*, *Cl. lusitaniae*, *Spathaspora arborariae,* and *Millerozyma*). Thus, the species clearly separate into two groups, one with many CUG codons almost exclusively at highly conserved leucine positions, and the other with CUG codons at serine positions (fig. 4). At a modest sequence conservation level (conservation score ≥ 50%), 5,591 (61.1%) of the CUG codons of the first group are at leucine positions (the maximum is 70.2% of the CUG codons in *Sa. kudriavzevii*). At this conservation level, 54 CUG codons (0.6%) are at modestly conserved serine positions (conservation score ≥50%). At the same conservation level, 17% of the CUG codons of the second group (in total 332 codons) are at serine positions, whereas 44 CUG codons (2.4%) are at leucine positions with the same conservation level. The same pattern can be observed when using scores calculated column wise instead of window wise (window size 0; supplementary fig. S4, Supplementary Material online).

### CUG Codon Positions Shared between Species

**Fig. 5.—:**
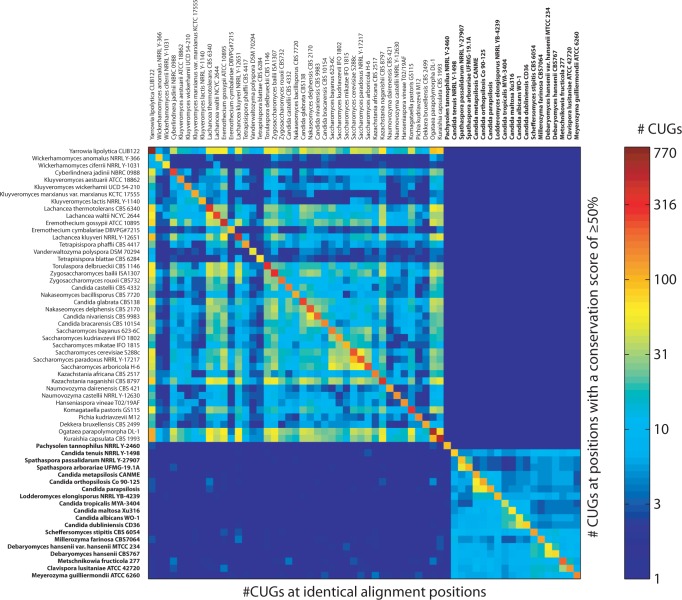
CUG codon positions conserved between species. The heatmap represents the number of CUG codon positions shared between every two species. The upper triangle shows the number of shared CUG codon positions at those positions in the concatenated alignment of the full-length sequences, which have a conservation score of at least 50%, the lower triangle the number of shared CUG codons at all alignment positions. The diagonal represents the total number of CUG codons in the respective species. The number of CUG codons is colored on a logarithmic scale. Species encoding CUG as serine are typed in bold.

It was proposed that all CUG codons had been removed during the time of codon ambiguity and later reintroduced within the Saccharomycetaceae and Candida branches (Massey et al. 2003). If this model was true, species of the Saccharomycetaceae and Candida would be expected to have shared CUG codon positions within but not across these two branches. In order to evaluate CUG codon position conservation, we determined the number of CUG codons shared between every two species (fig. 5). This CUG codon position analysis is independent from CUG codon assignment. For comparison, we analyzed the conservation of the CUG codon positions at all alignment positions (fig. 5, lower triangle) and at positions with a modest conservation level of more than or equal to 50% (fig 5, upper triangle). Independent of whether sequence conservation at alignment positions is required the yeast species group into two distinct classes. Species of the first group containing the *Saccharomycetaceae*, *Pfaffomycetaceae*, *Pichiaceae*, and *Ogataea* species, and *Dek. bruxellensis*, *Ko. pastoris*, *Ku. capsulata*, and *Yar. lipolytica* share considerably more CUG positions than those from the second group formed by the CTG-clade species. When all CUG positions are considered, there are a few positions shared between the two groups. However, if only CUG positions at a modest conservation level of more than or equal to 50% are evaluated, the two groups do not share any CUG positions. The few CUG positions shared at all alignment positions between the two species groups reflect CUG positions in disordered and nonconserved protein regions (see, e.g., the alignment of the class-1 myosin tail regions in supplementary fig. S2, Supplementary Material online). Interestingly, *Pachysolen* does not share any CUG positions with any other species (fig. 5).

### Mapping the Assigned Codon Usage onto the Species Tree

**Fig. 6.—:**
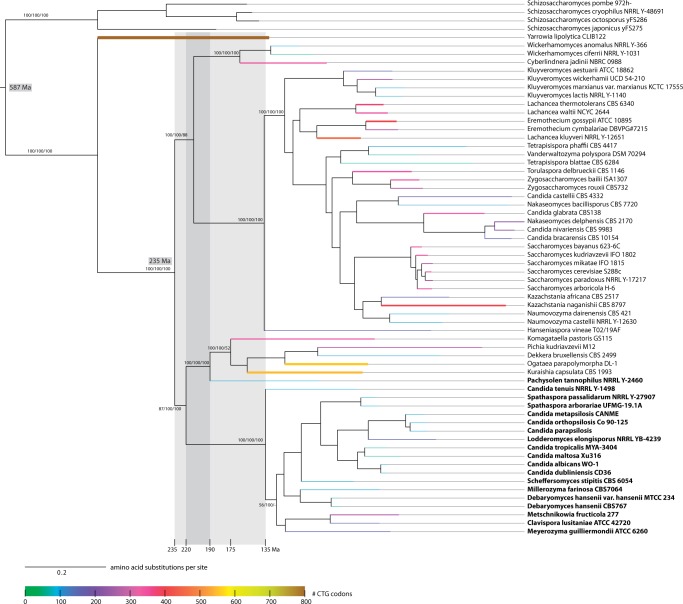
Timeline of the CUG codon reassignment. The tree presents the ML topology generated under the Γ + WAGF model in RAxML showing branch lengths for the concatenated alignments of 26 cytoskeletal and motor proteins. Support for major branches of the RAxML (1,000 bootstrap replicates), MrBayes (posterior probabilities), and ClustalW trees (1,000 replicates) is indicated (for more details, see supplementary fig. S1, Supplementary Material online). Species using the AYCU are highlighted in bold. With the splits of *Schizosaccharomycetes pombe* and *Saccharomyces cerevisiae* and *Sa. cerevisiae* and *Candida albicans* set to 587 and 235 Ma, respectively, the divergence of the CTG clade was estimated to 190 Ma using treePL (Smith and O’Meara 2012). The scale bar denotes amino acid substitutions per site. The width and color of the branches to extant species represent the total number of CUG codons in the respective concatenated sequences.

In order to determine the mono- or polyphyly of the AYCU assignment, we mapped the assigned codon usage onto the reconstructed species tree (fig. 6). This mapping supports the polyphyly of the AYCU assignment because the CTG clade and *Pachysolen* branched at different points during the evolution of the Saccharomycetales. The CUG codon position conservation is also in accordance with the species tree and the codon usage assignment. The CTG clade species group together in the species tree and share CUG codon positions implying that at least some of them have been introduced in the last common ancestor of the CTG clade. *Pachysolen* diverged separate from the CTG clade and therefore does not share any CUG codon positions with species from the CTG clade.

### Timeline for the Separation of the CTG Clade

In order to estimate the time span, in which the CUG codon usage might have been ambiguous, we applied published branching time estimations to our species trees. However, the divergence time estimations for the splits between Archiascomycetes and other Ascomycota and within the Saccharomycetes differ by more than 700 Myr although extensive molecular and fossil record data have been used in respective studies (Heckman et al. 2001; Douzery et al. 2004; Hedges et al. 2004; Berbee and Taylor 2007; for more references, see Hedges et al. 2006). Therefore, we choose some of the most extreme estimates for the calibration (table 1). The divergence time of the CTG clade was calibrated on the basis of the topology and branch lengths of the ML tree under penalized-likelihood constraints as implemented in treePL (Smith and O’Meara 2012). The splits of *Sc. pombe* and *Sa. cerevisiae,* and of *Sa. cerevisiae* and *Ca. albicans* were set to 1,144 and 841 Ma (Heckman et al. 2001), and 587 and 235 Ma (Douzery et al. 2004), respectively. Under these two sets of constraints, the separation of the CTG clade was estimated to 440 and 135 Ma, respectively (table 1), implying that the reassignment of the CUG codon in the CTG clade happened at most 400 and 100 Myr, respectively, after the split of the *Saccharomycetaceae* and the CTG clade (fig. 6). Similarly, a date of at least 190 Ma (640 Ma, respectively) was obtained for the emergence of *Pachysolen* indicating that ancient yeast species emerging in the time of 220–190 Ma (740–640 Ma, respectively) could have independently reassigned the CUG codon translation. This time span is the minimum time of CUG codon ambiguity. The maximum time span depends on the last split before the first appearance of the AYCU (split of the *Saccharomycetaceae* and the branch containing the CTG clade, Pichiaceae, and Ogataea; fig. 6) and both the first split after the last assignment of the AYCU (split of the branch containing Pichiaceae and Ogataea from *Pachysolen*) and the divergence of the CTG clade (135 and 440 Ma, respectively). The maximum time span of codon ambiguity is thus estimated to be 100 Myr (400 Myr, respectively).

**Table 1:** Estimation of the Time Span of the CUG Codon Reassignment

*Sc. pombe–Sa. cerevisiae*	*Sa. cerevisiae– Ca. albicans*	CUG Codon Ambiguity	CUG Codon Reassignment
1,850	**841^a^**	520–841	740–855
**1,144^a^**	730	415–730	605–695
**1,144^a^**	**841^a^**	440–841	640–740
485	**235^b^**	135–235	195–225
**587^b^**	360	205–360	295–340
**587^b^**	**235^b^**	135–235	190–220

Note.—Estimated times in million years for the split between *Sa. cerevisiae* and *Ca. albicans*, *Sa. cerevisiae* and *Sc. pombe*, and the reassignment of the CUG codon were calculated with a Penalized-likelihood program for the phylogenetic tree generated with RAxML using the WAG + Γ + F model. Time constrains for the splits between *Sa. cerevisiae* and *Ca. albicans*, and *Sa. cerevisiae* and *Sc. pombe* as reported by ^a^Heckman et al. (2001) and ^b^Douzery et al. (2004), respectively, were included both individually and combined. For each CUG codon reassignment time computation, constrained time estimates are typed in bold. The CUG codon reassignment time estimates are given as differences between the time estimates for the divergence of *Pachysolen tannophilus* from the Pichiaceae and Ogataea species (first number), and the first occurrence of the ${\text{tRNA}}_{\text{CAG}}^{\text{Ser}}$ (second number; separation of the CTG clade from the branch containing *Pa. tannophilus*, the Pichiaceae and Ogataea species). The CUG codon ambiguity time estimates, however, are given as differences between the time estimates for the latest possible fixation of the ${\text{tRNA}}_{\text{CAG}}^{\text{Ser}}$ (first number; separation of *Ca. tenuis*, the most basal species in the CTG clade) and the earliest possible invention of the ${\text{tRNA}}_{\text{CAG}}^{\text{Ser}}$ (second number; separation of the Saccharomycetaceae and the CTG clade). The time estimate for the divergence of *Sc. pombe* and *Sa. cerevisiae* (1,850 Ma) based on the constraint of 841 Ma for the split of *Sa. cerevisiae* and *Ca. albicans* predates the emergence of eukaryotes and should be ignored.

## Discussion

The AYCU has already been assigned to ten of the sequenced yeast genomes: *Ca. albicans* (Jones et al. 2004; Butler et al. 2009), *Ca. dubliniensis* (Jackson et al. 2009), *Ca. parapsilosis* (Butler et al. 2009), *Ca. tropicalis* (Butler et al. 2009), *Cl. lusitaniae* (Butler et al. 2009), *Debaryomyces hansenii* (Dujon et al. 2004; Butler et al. 2009), *Lodderomyces elongisporus* (Butler et al. 2009), *Me. guilliermondii* (Butler et al. 2009), *Scheffersomyces stipites* (Jeffries et al. 2007), and *Spathaspora passalidarum* (Wohlbach et al. 2011), and the standard codon usage was shown for several of the *Saccharomyces* species. Mass spectrometry and Edman sequencing of salt-mediated killer toxin demonstrated that *Millerozyma farinosa* also uses the AYCU (Suzuki et al. 2002). An in vitro translation study showed 78 *Candida* species to use the AYCU including the above listed *Candida* strains (Sugita and Nakase 1999), and in addition *Ca. tanzawaensis* and *Ca. tenuis*, for which the genome sequence had been assembled recently (Wohlbach et al. 2011). Nevertheless, the gene prediction data sets for *Ca. tanzawaensis* and *Ca. tenuis* available at the respective sequencing centers include predictions both with AYCU and with standard codon usage. However, for highly resolved phylogenetic studies, protein ortholog assignments and functional studies, naming only a few applications, the accuracy of the protein sequences is important. The best and most accurate way to determine a species codon usage would be the experimental analysis of some proteins known to contain CUG codons by, for example, mass spectrometry. However, as long as proteins cannot be enriched this is extremely difficult to conduct, and the most prevalent endogenous proteins might not contain CUG codons. For example, our analysis showed that actin genes from only a few yeasts (11 of 60 analyzed) contain CUG codons. Other approaches only provide indirect evidence. As discussed, the presence or absence of cellular components such as galactose or Q9 does not correlate with CUG codon usage. Here, we hypothesized that the codon usage can unambiguously be assigned by analyzing three types of data: A highly resolved phylogenetic tree, sequence conservation at CUG coding positions, and conservation of CUG codon positions across species. To obtain significant numbers of sequences, we were restricted to analyze sequenced genomes. Up to May 2014, genomes of 60 yeast species have been sequenced, assembled, and released. We did not include genomes in the analysis, which were sequenced with low-coverage, and only included one strain per species (e.g., 73 different *Sa. cerevisiae* strains have been sequenced, of which only the reference strain was included in our analysis). To avoid bias by misassigning CUG codons, we excluded all CUG positions from the alignment and the sequence conservation calculations.

For the reconstruction of the yeast tree we concatenated 26 cytoskeletal and motor proteins, which are key-components of eukaryotic cells. We decided to only use proteins in the analysis that are at least present in all unikonts (the eukaryotic super-kingdom including Fungi, Metazoa, Apusozoa, and Amoebozoa) to ensure that we do not have extensive missing data. The topology of the species tree was almost identical independent of the reconstruction method used (NJ, ML, and Bayesian; fig. 6). Although this particular set of species has never been analyzed before, the branching of most subbranches and individual species is comparable to those found in previously published yeast trees (see references in Introduction section). The species known to use either the standard code (*Saccharomycetaceae*) or the AYCU (see above) grouped into two distinct clades. It seemed obvious to assume, but had to be shown, that all other species within these two groups, for which the codon usage has not yet been confirmed, use the same codon usage as the known species. In terms of a mono- or polyphyletic origin of the AYCU, it would be necessary to resolve the codon usage of the species of the other major branches (*Phaffomycetaceae* clade and the branch containing the *Pichiaceae* and *Ogataea*) and the species that diverged before the combined *Saccharomycetaceae*/CTG clades such as *Yar. lipolytica*.

To determine the most probable CUG codon usage for each species, we analyzed the amino acid conservation at the respective CUG codon positions in the protein sequence alignments. Amino acid positions highly conserved over hundreds of million years (divergence time of Schizosaccharomyces and Saccharomycetes) are expected to represent structurally and functionally important sites in proteins. A small polar residue at a position, which is characterized by conserved large hydrophobic residues in the other species, seems very unlikely. Similarly, large hydrophobic residues at positions conserved in small and polar amino acids should be strongly disfavored. Our data show that the assignment of the standard codon usage is facilitated by the enrichment of CUGs at highly conserved leucine positions. In this respect only determining the class I myosin of a species with unknown CUG codon usage and comparing this sequence to the available data (fig. 2 and supplementary fig. S2, Supplementary Material online) would in most cases be enough to find out whether the respective species uses the standard or the alternative yeast CUG codon translation. If CUG codons can be mapped to conserved leucine positions, it will be highly probable that the respective species translates CUG as leucine. The AYCU will be assigned to a species if CUG codons are mapped to highly conserved serine positions or if CUG codons are not present at highly conserved leucine positions (proof by contradiction). In addition to the class I myosins, several proteins could be identified and compared until a conserved serine position was found. The latter approach would lead to an unambiguous assignment and was used in our analysis here. It has been proposed that CUG codons were not reintroduced randomly in CTG clade genomes but by avoiding structurally sensitive sites (Rocha et al. 2011). This is in agreement with the different biochemical properties of leucine and serine and the associated consequences for protein folding and solubility. Leucine positions are in general stronger conserved than serine positions, leucines are usually located in the core of protein domains, and mutations at leucine positions are therefore restricted. Compared with serine, the introduction of leucine-encoding CUG codons is possible by synonymous mutations from CUN codons or by transition from thymine from the UUG codon. Transitions are more favorable and generated at higher frequencies than transversions. Such silent substitutions seem to be very common given the low conservation of the CUG codons within the yeast species using the Standard Code. In contrast, mutating an existing serine codon to a CUG codon would require at least two mutations. Mutations from other codons than leucine or serine codons would either require the less likely transversions (e.g., GUG to CUG) or very unusual amino acid mutations (e.g., from proline to serine). Therefore, it is not surprising to find less CUG codons in CTG clade species, and those codons at less conserved positions. Overall, we could demonstrate that especially the CUG codons coding for leucine, and also a substantial number of the CUG codons in species using the AYCU, are at conserved alignment positions (fig. 4). Although seven of the CUG codons coding for leucine and serine, respectively, are at positions supporting the respective opposite codon translation based on an amino acid conservation level of more than or equal to 80%, the majority of the CUG codon positions supports either the standard or the AYCU. This is in accordance with protein structure and in vivo studies showing that single point mutations and even partial reversion of the CUG identity are tolerated in *Ca. albicans* although large numbers of mutations cause profound cellular changes such as morphological variation and altered gene expression (Cutfield et al. 2000; Gomes et al. 2007; Miranda et al. 2007; Rocha et al. 2011; Miranda et al. 2013; Bezerra et al. 2013). Because we excluded all CUG codon positions from the analysis, our assignments are independent from previous knowledge. Our data unambiguously support the assignment of the standard codon usage to the *Saccharomycetaceae*, and the AYCU to the eight species, which were known to translate CUG as serine. From these data, we conclude that the CUG codon assignment can unambiguously be resolved by analyzing a considerable amount of sequences and that our CUG codon translation assignment is robust. Therefore, we assigned the AYCU to the CTG clade species and *Pachysolen*. Although our data strongly support an unambiguous assignment of the CUG codon usage, we acknowledge that the final judgment should come from experiments.

When plotting the assigned AYCU onto the species in the phylogenetic trees it became evident that the CUG codon usage must have been ambiguous for a long time in the ancestry of the Saccharomycetes leading to polyphyly of the AYCU (fig. 6). This means that ancestral species that emerged during this time span could have adopted either the standard or the AYC usage. In the case of a long-time span of codon ambiguity, the original CUG codons in the ancestor of the Saccharomycetes were most likely erased and later reintroduced in the emerging branches either as leucine codons or as serine codons. In the less likely and mutually exclusive scenario, the CUG ambiguity appeared only once in the ancestor of the CTG clade. The combination of the reconstructed species trees with our CUG codon assignment strongly supports the first scenario of a polyphyletic origin of the AYCU.

Based on the phylogeny of the analyzed species *Pachysolen* does not group to the CTG clade. In addition, *Pachysolen* does not have any CUG codons at highly conserved leucine positions, but at conserved serine positions. The phylogenetic grouping of *Pachysolen* has not been analyzed in detail so far, and not at all in the context of a taxonomic sampling as used in our study. The evolutionary grouping and CUG codon usage were also not analyzed in the study of the *Pachysolen* draft genome sequence (Liu et al. 2012). A previous study based on 28s rDNA and 18s rDNA only resolved the *Saccharomycetes* but not the grouping of *Pachysolen* and none of the other clades shown to be monophyletic in our trees (including the CTG clade). In addition, support for the branchings was low (Suh et al. 2006). In contrast, we used a phylogenomics approach for the tree reconstruction resulting in strongly supported branching of *Pachysolen*.

The separate branching of *Pachysolen* is in agreement with the previously proposed model of a long-time span of codon usage ambiguity (Sugita and Nakase 1999). If the CUG codon usage was ambiguous for a long time, it would be natural to assume that species diverged within this time span, and that those species could have adapted either of the usages. Supposed, that the codon usage was ambiguous for a long time, it seems highly nonparsimonious, that either species did not branch within this time span or that all species except the ancestor of the CTG clade adapted the standard codon usage. A monophyletic group of the species using the AYCU is also highly unlikely given the missing conservation of CUG codon positions between the CTG clade species and *Pachysolen*. To date the codon reassignments, we dated the ML tree based on various separation time estimates for the splits of *Sc. pombe* and *Sa. cerevisiae*, and *Sa. cerevisiae* and *Ca. albicans* (fig. 6 and table 1). Accordingly, there is a maximum time span of codon ambiguity of 100–400 Myr ranging from the first possible appearance of the serine ${\text{tRNA}}_{\text{CAG}}^{\text{Ser}}$ (separation of the Saccharomycetes and the branch containing the CTG clade, the Pichiacea and Ogataea) to the split of the most basal species of the CTG clade (*Ca. tenuis*). Assuming an unambiguous usage of the CUG codon in *Pachysolen* and the CTG clade, the minimal time span of CUG codon ambiguity was 30–100 Myr (fig. 6 and table 1). This is well in agreement with a previous study, which suggested an ambiguous usage of the CUG codon for 100 Myr (Sugita and Nakase 1999). However, the presented time estimates are based on the species analyzed in this study. It is well reasonable to assume that further sequenced species will change the time estimates in two directions (this might already happen by analyzing the yeast species whose data are still under embargo). The inclusion of more basal CTG clade species will decrease the current codon ambiguity time. The identification of further species using the AYCU, which do not group to the CTG clade or to *Pachysolen*, instead might increase both the codon ambiguity and codon reassignment time ranges. Such species include yeasts, which might even branch before the split of the Saccharomycetaceae and the CTG clade/Pichiacea/Ogataea, as well as additional species diverging in the sister branch of the CTG clade, and species separating early in the Saccharomycetaceae branch.

## Supplementary Material

Supplementary files S1–S4 and figures S1–S4 are available at *Genome Biology and Evolution* online (http://www.gbe.oxfordjournals.org/).

Supplementary Data

## Notes

This manuscript is a revised version of a previous manuscript, which was retracted because of unresolved issues caused by changed data usage policies. It is very similar to the previous version except that protein sequences predicted and annotated based on genome assemblies produced by the DOE Joint Genome Institute have been removed. Instead, sequences from another twelve yeasts have been added, which were not available when finishing the analysis for the previous manuscript, to improve the statistical basis for the CTG codon assignment.
