# Supplementary material for: Molecular Phylogeny of Sequenced Saccharomycetes Reveals Polyphyly of the Alternative Yeast Codon Usage
Source: Genome Biol Evol. 2014 Jul 22;6(12):3222–37. doi: 10.1093/gbe/evu152 (PMC4986446; doi:10.1093/gbe/evu152)
Supplement: Supplementary Data [file supp_6_12_3222__index.html]

Molecular Phylogeny of Sequenced Saccharomycetes Reveals Polyphyly of the Alternative Yeast Codon Usage — Molecular Phylogeny of Sequenced Saccharomycetes Reveals Polyphyly of the Alternative Yeast Codon Usage — Supplementary Data 

# Molecular Phylogeny of Sequenced *Saccharomycetes* Reveals Polyphyly of the Alternative Yeast Codon Usage

## Supplementary Data

files

**Files in this Data Supplement:**

- Supplementary Data - zip file
