# Supplementary material for: Molecular Phylogeny of Sequenced Saccharomycetes Reveals Polyphyly of the Alternative Yeast Codon Usage
Source: Genome Biol Evol. 2014 Jul 22;6(12):3222–37. doi: 10.1093/gbe/evu152 (PMC4986446; doi:10.1093/gbe/evu152)
Supplement: Supplementary Data [file supp_evu152_suppl_data.zip › FigureS1.pdf]

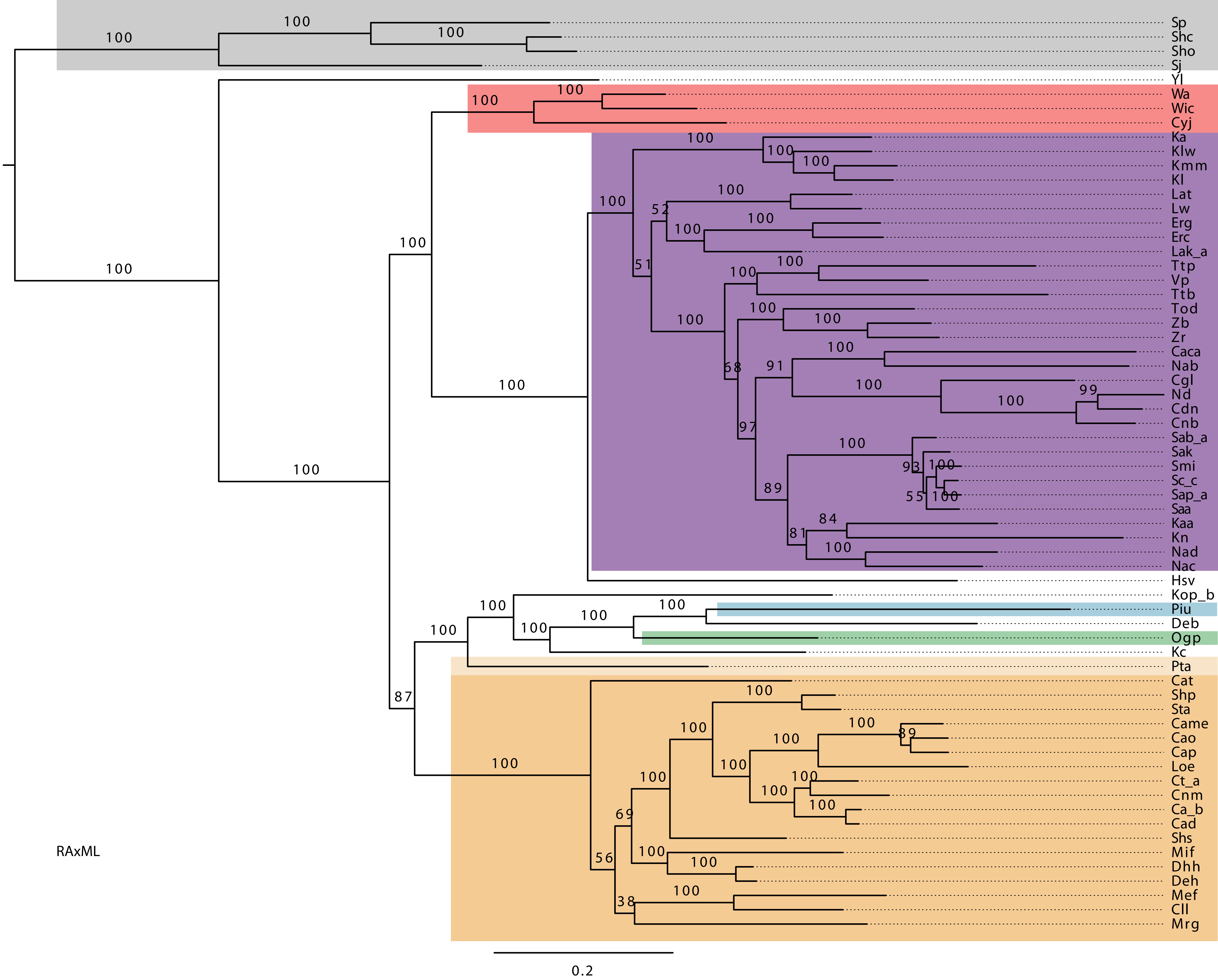
Sp
Shc
Sho
Sj
Yl
Wa
Wic
Cyj
Ka
Klw
Kmm
Kl
Lat
Lw
Erg
Erc
Lak_a
Ttp
Vp
Ttb
Tod
Zb
Zr
Caca
Nab
Cgl
Nd
Cdn
Cnb
Sab_a
Sak
Smi
Sc_c
Sap_a
Saa
Kaa
Kn
Nad
Nac
Hsv
Kop_b
Piu
Deb
Ogp
Kc
Pta
Cat
Shp
Sta
Came
Cao
Cap
Loe
Ct_a
Cnm
Ca_b
Cad
Shs
Mif
Dhh
Deh
Mef
Cll
Mrg
RAxML
0.2

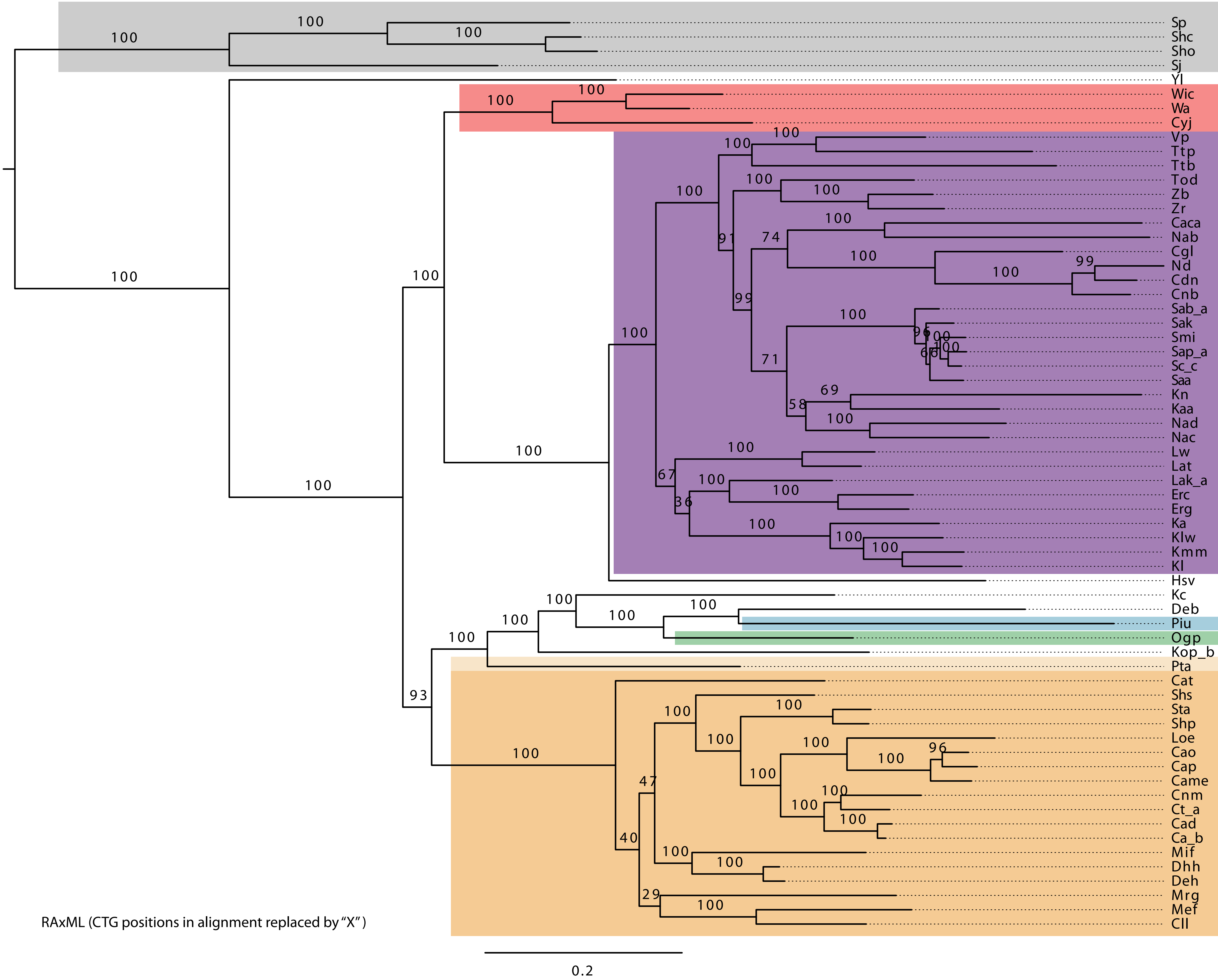
Sp
Shc
Sho
Sj
Yl
Wic
Wa
Cyj
Vp
Ttp
Ttb
Tod
Zb
Zr
Caca
Nab
Cgl
Nd
Cdn
Cnb
Sab_a
Sak
Smi
Sap_a
Sc_c
Saa
Kn
Kaa
Nad
Nac
Lw
Lat
Lak_a
Erc
Erg
Ka
Klw
Kmm
Kl
Hsv
Kc
Deb
Piu
Ogp
Kop_b
Pta
Cat
Shs
Sta
Shp
Loe
Cao
Cap
Came
Cnm
Ct_a
Cad
Ca_b
Mif
Dhh
Deh
Mrg
Mef
Cll
RAxML (CTG positions in alignment replaced by "X")
0.2

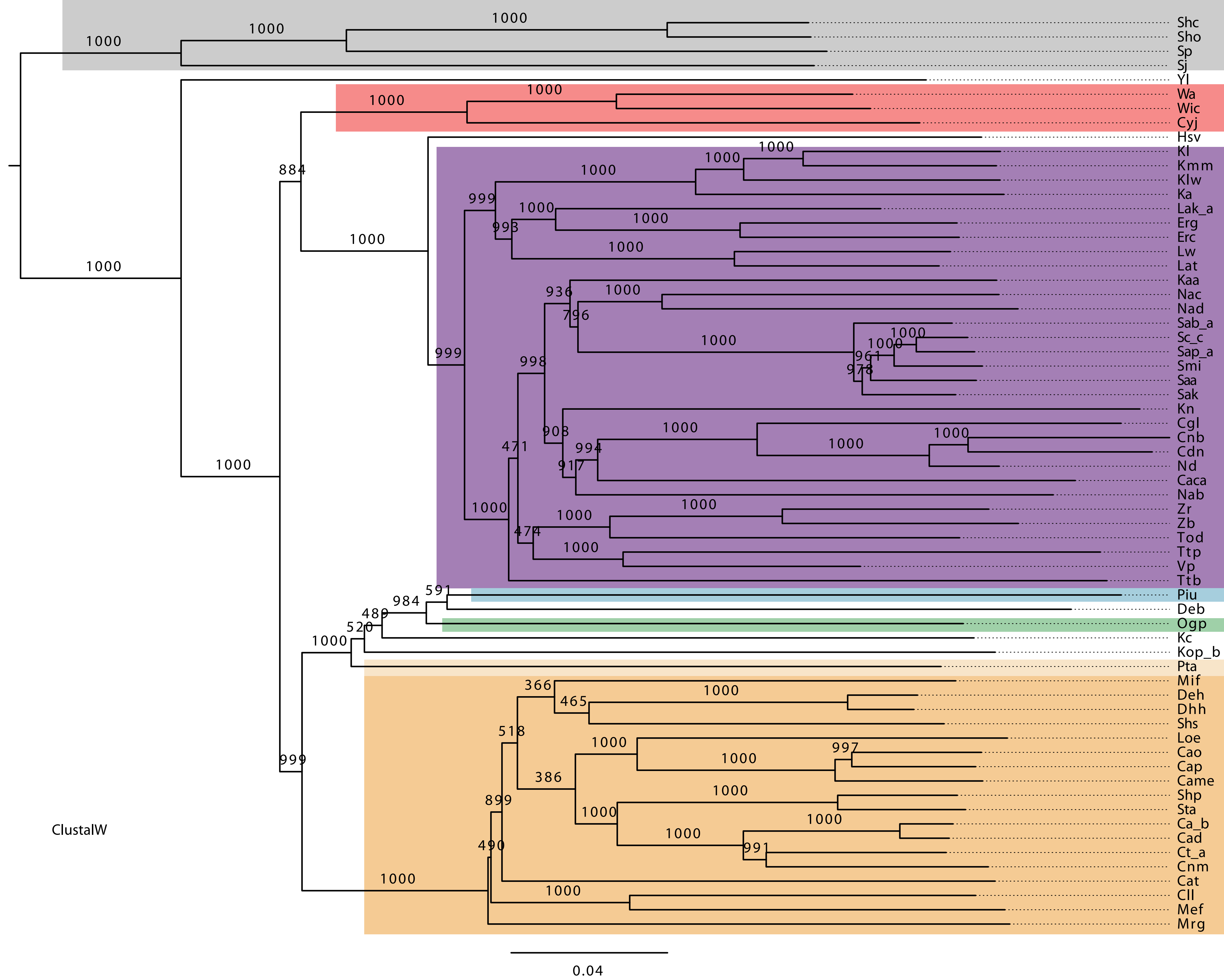
Shc
Sho
Sp
Sj
Yl
Wa
Wic
Cyj
Hsv
Kl
Kmm
Klw
Ka
Lak_a
Erg
Erc
Lw
Lat
Kaa
Nac
Nad
Sab_a
Sc_c
Sap_a
Smi
Saa
Sak
Kn
Cgl
Cnb
Cdn
Nd
Caca
Nab
Zr
Zb
Tod
Ttp
Vp
Ttb
Piu
Deb
Ogp
Kc
Kop_b
Pta
Mif
Deh
Dhh
Shs
Loe
Cao
Cap
Came
Shp
Sta
Ca_b
Cad
Ct_a
Cnm
Cat
Cll
Mef
Mrg
1000
884
999
993
936
796
961
978
998
908
471
994
917
474
591
984
489
520
366
465
518
997
386
899
490
991
ClustalW
0.04

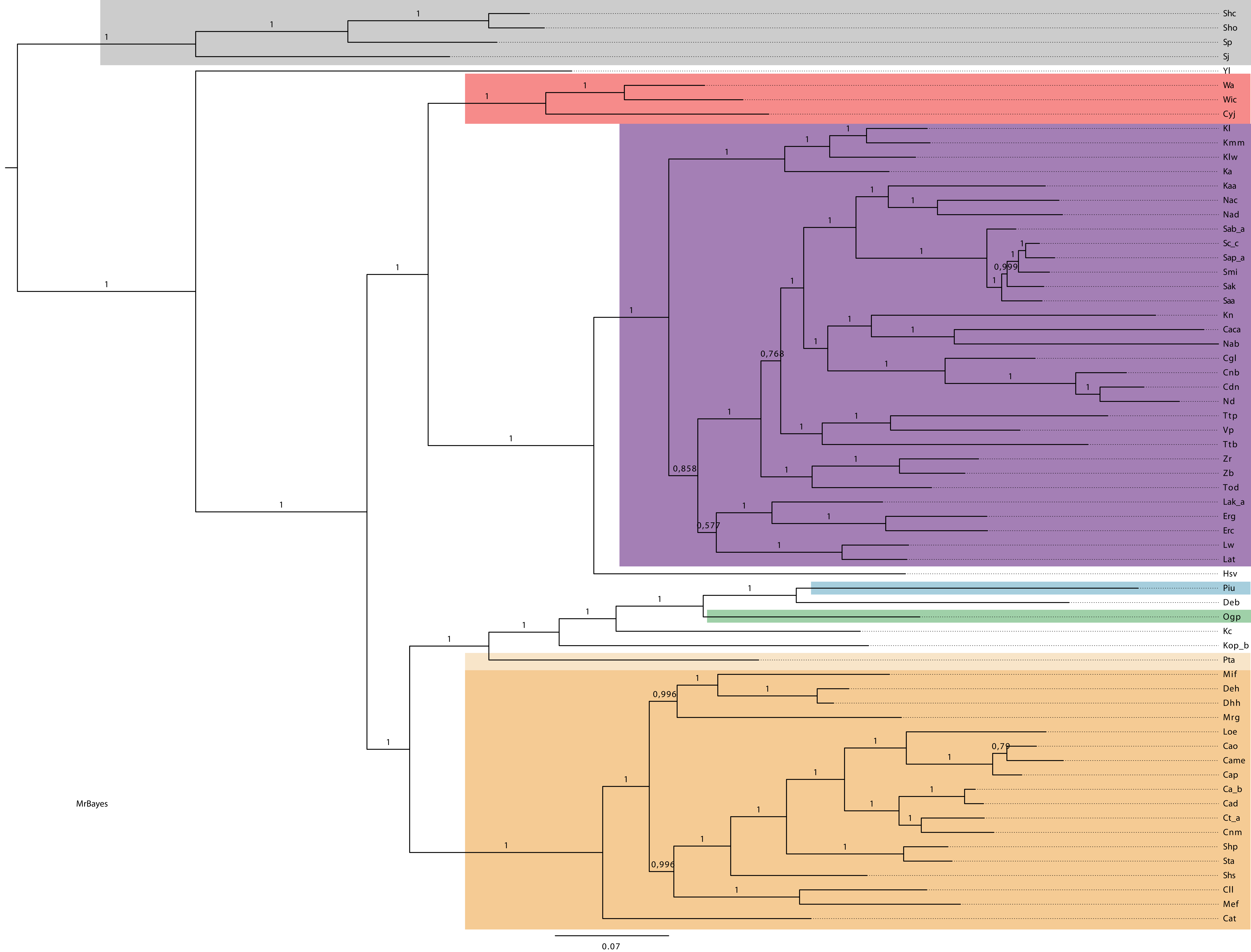
Shc
Sho
Sp
Sj
Yl
Wa
Wic
Cyj
Kl
Kmm
Klw
Ka
Kaa
Nac
Nad
Sab_a
Sc_c
Sap_a
Smi
Sak
Saa
Kn
Caca
Nab
Cgl
Cnb
Cdn
Nd
Ttp
Vp
Ttb
Zr
Zb
Tod
Lak_a
Erg
Erc
Lw
Lat
Hsv
Piu
Deb
Ogp
Kc
Kop_b
Pta
Mif
Deh
Dhh
Mrg
Loe
Cao
Came
Cap
Ca_b
Cad
Ct_a
Cnm
Shp
Sta
Shs
Cll
Mef
Cat
MrBayes
0.07

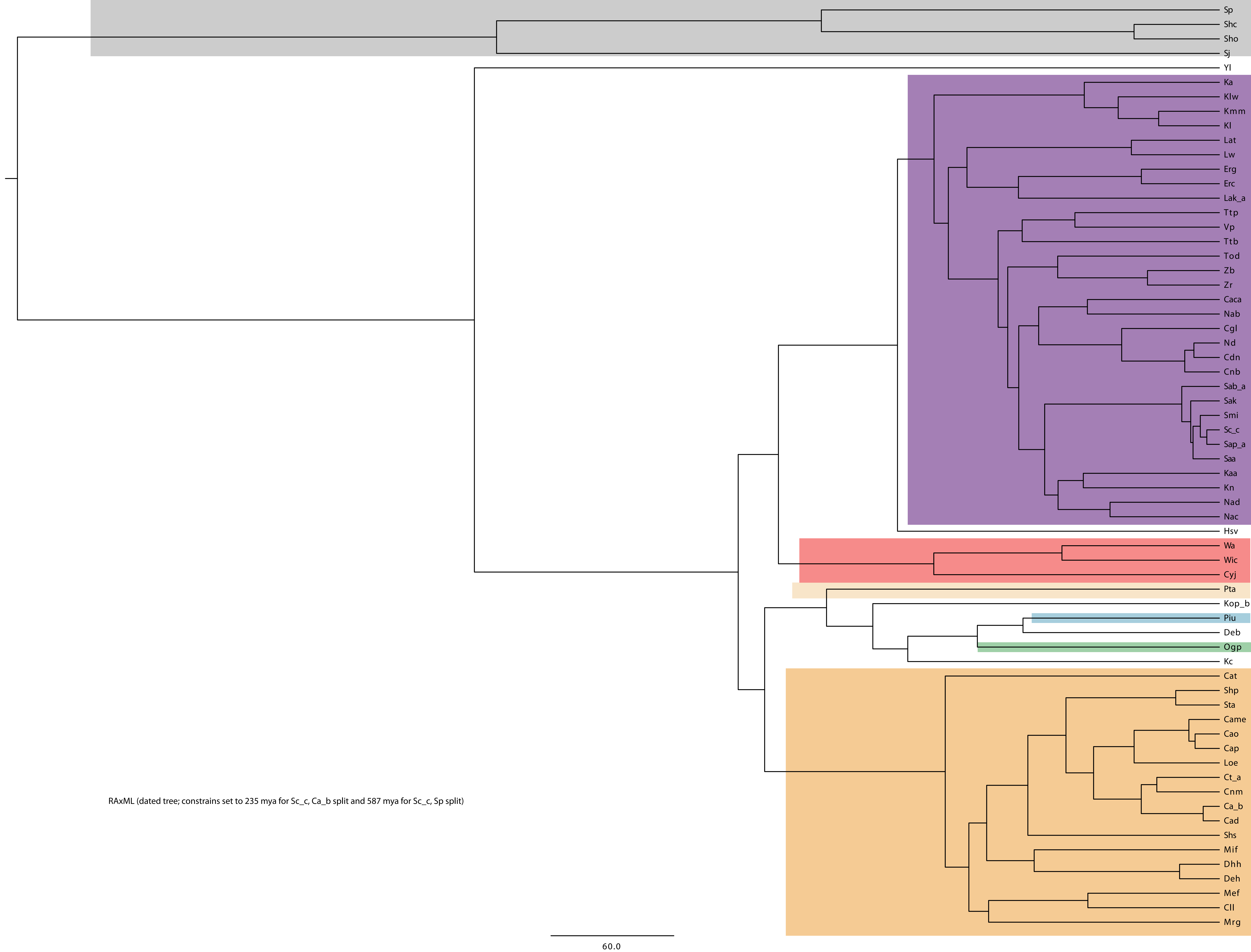
Sp
Shc
Sho
Sj
Yl
Ka
Klw
Kmm
Kl
Lat
Lw
Erg
Erc
Lak_a
Ttp
Vp
Ttb
Tod
Zb
Zr
Caca
Nab
Cgl
Nd
Cdn
Cnb
Sab_a
Sak
Smi
Sc_c
Sap_a
Saa
Kaa
Kn
Nad
Nac
Hsv
Wa
Wic
Cyj
Pta
Kop_b
Piu
Deb
Ogp
Kc
Cat
Shp
Sta
Came
Cao
Cap
Loe
Ct_a
Cnm
Ca_b
Cad
Shs
Mif
Dhh
Deh
Mef
Cll
Mrg
RAxML (dated tree; constrains set to 235 mya for Sc_c, Ca_b split and 587 mya for Sc_c, Sp split)
60.0
