# Supplementary material for: Molecular Phylogeny of Sequenced Saccharomycetes Reveals Polyphyly of the Alternative Yeast Codon Usage
Source: Genome Biol Evol. 2014 Jul 22;6(12):3222–37. doi: 10.1093/gbe/evu152 (PMC4986446; doi:10.1093/gbe/evu152)

```
                        10        20        30        40        50        60        70        80        90       100
              ....|....|....|....|....|....|....|....|....|....|....|....|....|....|....|....|....|....|....|....|
CnbMyo1A      1 -MALLKRGARNKTQ---QEPPKKG-----------GKIKKATFESSKKKEVGVSDLTLLTTISDESINDNLKKRFLNGSIYTYIGHVLISVNPFRDLGIY
CdnMyo1A      1 -MALLKRGARNKTQ---QEPPKKG-----------GKIKKATFESSKKKEVGVSDLTLLTTISDESINDNLKKRFQNGTIYTYIGHVLISVNPFRDLGIY
CacaMyo1A     1 -MGLIKRTARNKTS---QEPAKRS-----------THIKKATFDSSKKKEVGVSDLTLLSTISDESINDNLKKRFQNGTIYTYIGHVLISVNPFRDLGIY
NabMyo1A      1 -MALIKRKVRTKGT---QEPPKRS-----------GQIKKATFDSSKKKEVGVSDLTLLSTISDEAINDNLKKRFQNGSIYTYIGHVLISVNPFRDLGIY
NdMyo1A       1 -MALLKRGARNKTQ---QEPPKKG-----------GKIKKATFEPAKKKEVGVSDLTLLTTISDESINENLKKRFLNGTIYTYIGHVLISVNPFRDLGIY
CnbMyo1B      1 -MAIIKKVARSKAG---QAPAKRT-----------TKIRKATYDSSKKKEIGVSDLTLLSKISDESINDNLKRRFEHGIIYTYIGYVLISVNPFRDLGIY
CglMyo1A      1 -MAILKRGARNKTH---QEPAKRGG----------NNIKKAAFETSKKKEVGVSDLTLLTSISDESINDNLKKRFLNGTIYTYIGHVLISVNPFRDLGIY
Sab_aMyo1B    1 -MAILKRGARKKVH---QEPAKRS-----------ANIKKATFDSSKKKEVGVSDLTLLSKISDEAINENLKKRFQNATIYTYIGHVLISVNPFRDLGIY
Sc_cMyo1B     1 -MAILKRGARKKVH---QEPAKRS-----------ANIKKATFDSSKKKEVGVSDLTLLSKISDEAINENLKKRFLNATIYTYIGHVLISVNPFRDLGIY
SaaMyo1B      1 -MAILKRGARKKAH---QEPVKRS-----------ANIKKATFDSSKKKEVGVSDLTLLSKISDEAINDNLKKRFQNATIYTYIGHVLISVNPFRDLGIY
SakMyo1B      1 -MAILKRGARKKAH---QEPAKRS-----------TNIKKATFDSSKKKEVGVSDLTLLSKISDEAINENLKKRFQNATIYTYIGHVLISVNPFRDLGIY
SmiMyo1B      1 -MAILKRGARKKAH---QEPAKRS-----------ANIKKATFDSSKKKEVGVSDLTLLSKISDEAINENLKKRFMNATIYTYIGHVLISVNPFRDLGIY
Sap_aMyo1B    1 -MAILKRGARKKVH---QEPAKRS-----------ANIKKATFDSSKKKEVGVSDLTLLSQISDEAINENLKKRFLNATIYTYIGHVLISVNPFRDLGIY
NacMyo1       1 -MALLKRGARNKNA---QEPAKRS-----------AKIKKATFDATKKKEVGVSDLTLLSKISDETINDNLKKRFMNGTIYTYIGHVLISVNPFRDLGIY
KaaMyo1       1 -MALIKRGARSKTA---QAPQKRA-----------ANIKKATFDSSKKKEVGVSDLTLLSKISDQAINENLKKRFQNGTIYTYIGHVLISVNPFRDLGIY
VpMyo1        1 -MAIIKRGARNKTA---QEPAKRS-----------AKIKKATYDSGKKKEVGVSDLTLLSTISDEAINENLKKRFTNGTIYTYIGHVLISVNPFRDLGIY
TtpMyo1A      1 -MAIIKRGARNKSA---QEPEKRS-----------AKIKKATFDVGKKKEVGVSDLTLLTKISDESINENLKKRFLNGIIYTYIGHVLISVNPFRDLGIY
TtpMyo1B      1 -MAIIKRGARNKSA---QEPEKRS-----------AKIKKATFDVGKKKEVGVSDLTLLTKISDESINENLKKXFLNGIIYTYIGHVLISVNPFRDLGIY
TtbMyo1A      1 -MAIIKRGARDRGIT--RESNQRSG----------GKIKKASYDSSKKKEVGVSDLTLLSKISDEAINENLKKRFQNGLIYTYIGQVLISVNPFRDLGIY
TtbMyo1B      1 -MALLKRGGRAKIQQ--STTHKKNG----------NQIKKATFDSTKKKEVGVSDLTLLSTISDEAINDNLKKRFLNGTIYTYIGYVLISVNPFRDLGIY
ErgMyo1       1 -MAIIKRGVRQKTQ----PPAKRT-----------TNIKKATFDSGKKKEVGVSDLTLLSKISDEHINENLKRRFENGSIYTYIGHVLISVNPFRDLGIY
ErcMyo1       1 -MAIIKRGVRNKTAQ---PPVKRS-----------ANIKKATFDSGKKKEVGVSDLTLLSQITDSHINENLKKRFQNGTIYTYIGHVLISVNPFRDLGIY
HsvMyo1       1 -MAIIKRGARNKNQ---AAPEKRSA----------KKIQKAAFDTTKKKEVGVSDLTLLTKISNESINENLEKRFKNGIIYTYIGNVLISVNPFRDLGIY
KlMyo1        1 -MALIRR-AKNKVA-----PQKRAE----------TTIKKATFDATKKKEVGVSDLTLLSSISDDAINQNLKKRFENGTIYTYIGHVLISVNPFRDLGIY
KmmMyo1       1 -MAIIRR-AKNKPA-----PQKRAD----------ATIKKATFDATKKKEVGVSDLTLLSSISDDAINQNLKKRFENGTIYTYIGHVLISVNPFRDLGIY
KlwMyo1       1 -MAIIRR-AKNKAA-----PQKRAE----------ATIKKATFDAHKKKEVGVSDLTLLSSISDEAINNNLKKRFENGTIYTYIGHVLISVNPFRDLGIY
KaMyo1        1 -MALIRR-AKNKPA-----PQKRAEA---------GAIKKATFESNKKKEVGVSDLTLLSTITDDSINTNLKKRFQNGSIYTYIGHVLISVNPFRDLGIY
KnMyo1        1 -MALIKRGARKKTS---QAPEKRS-----------AKIKKATFDATKKKEVGVSDLTLLSTISDESINDNLKKRFLNGSIYTYIGHVLISVNPFRDLGIY
LwMyo1        1 -MAIIKRGARNKTA---QQPAKRS-----------GQIKKATFESNKKREVGVSDLTLLSKISDEAINENLKKRFQNGTIYTYIGHVLISVNPFRDLGIY
LatMyo1       1 -MAIIKRGARNKTA---QQPAKRS-----------GQIKKATFESNKKKEVGVSDLTLLSKISDESINDNLKKRFQNGTIYTYIGHVLISVNPFRDLGIY
Lak_aMyo1     1 -MAIIKRGARNKSA---QQPTKRSDGGK-------ANIKKATFESSKKKEIGVSDLTLLSKISDESINENLKKRFQNGTIYTYIGHVLISVNPFRDLGIY
Sab_aMyo1A    1 -MAVIKRGARKKDV---KEPKKRS-----------AKIKKATFDAHKKKEVGVSDLTLLSKISDESINENLKKRFKNGIIYTYIGHVLISVNPFRDLGIY
Sc_cMyo1A     1 -MAVIKKGARRKDV---KEPKKRS-----------AKIKKATFDANKKKEVGISDLTLLSKISDESINENLKKRFKNGIIYTYIGHVLISVNPFRDLGIY
SaaMyo1A      1 -MAVIKRGTRKKDV---KEPKKRS-----------AKIKKATFDANKKKEVGVSDLTLLSKISDESINENLKKRFKNGIIYTYIGHVLISVNPFRDLGIY
SakMyo1A      1 -MAVIKRGARKKDV---KEPKKRS-----------AKIKKATFDANKKKEVGVSDLTLLSKISDESINENLKKRFKNGFIYTYIGHVLISVNPFRDLGIY
SmiMyo1A      1 -MAVIKRGARRKDV---KEPKKRS-----------AKIKKATFDANKKKEVGVSDLTLLSKISDESINENLRKRFKNGIIYTYIGHVLISVNPFRDLGIY
Sap_aMyo1A    1 -MAVIKRGARRKDV---KEPKKRS-----------AKIKKATFDANKKKEVGVSDLTLLSKISDESINENLKKRFKNGIIYTYIGHVLISVNPFRDLGIY
ZrMyo1        1 -MAIIKKGARTKTA---AAPEKRS-----------ARIKKASFDTTKRKEVGVSDLTLLSTISDEAINENLKKRFKNGAIYTYIGHVLISVNPFRDLGIY
TodMyo1       1 -MAIIKRGARSKAT---AAPAKRS-----------AQIKKATYDSSKKNEVGVSDLTLLTTISDQSINENLKKRFLNGSIYTYIGHVLISVNPFRDLGIY
CglMyo1B      1 -MAIIKRVVRSKAG---QAPVKKG-----------AKIKKASYDSSRKKEVGVSDLTLLSKISDESINDNLKKRFEHGIIYTYIGYVLISVNPFRDLGIY
WaMyo1Alpha   1 -MGITRRAGKTAR----QQPPKRN-------------GIQKATFDAGKKKEVGVSDLTLLTKISDESINDNLKKRFENGTIYTYIGHVLISVNPFRDLGIY
WicMyo1       1 -MGITKRVGRSNK----QPPPKRN-------------GIQKATFDVGKKKEVGVSDLTLLTKISDESINENLQKRFDNGTIYTYIGHVLISVNPFRDLGIY
CyjMyo1       1 -MAITRRPGKGK-----QQPPKRN------------NGIQKATFEAHKKREVGVSDLTLLTKITDEAINENLKKRFENGTIYTYIGHVLISVNPFRDLGIY
YlMyo1        1 -MAVTKRAGRRAQG--GTQPAKGA------------QGVKKATFESGKKREVGVSDLTLLSKVSEEAINENLKKRFENGTIYTYIGHVLISVNPFRDLGIY
CdnMyo1B      1 -MAIIKRGARNKT----QAPAKRGG----------AKIKKATYDSSKKKEIGVSDLTLLSKISDDSINDNLKRRFEHGIIYTYIGYVLISVNPFRDLGIY
Kop_bMyo1     1 -MAITKRIGRNKQV--REQPAKR-------------GGVKKAEFDVHKKKEVGVSDLTLLSTISDNSINENLEKRFKNGTIYTYIGHVLISVNPFRDLGLY
NdMyo1B       1 -MAIIKRGARNKA----QAPAKRG-----------TKIKKATYDSSKKKEIGVSDLTLLSKISDDSINDNLKRRFQHGIIYTYIGYVLISVNPFKDLGIY
KcMyo1        1 -MAITKRAGRNKAHAANEQPPKR------------AAVKKAQFEATKKKEVGVSDLTLLSTISDVAINENLEKRFHNGTIYTYIGHVLISVNPFRDLGIY
OgpMyo1       1 MAVLSRKAVRTKNT-GTGPPPKKTN----------APVKKAQFESNKTKEVGVSDLTLLSTISDQTINDNLEKRFHHGTIYTYIGHVLISVNPFRDLGIY
DebMyo1       1 MAGFLRKRNRNRNK--TAPPPKKGG----------GKIKRAQYEHSRKKEVGVSDLTLLSTISDESINENLHKRFENGIIYTYIGQVLISVNPFRDLGIY
PiuMyo1       1 MMGFSKRS-RQKTA----APARQSASANG------GGIKKATYEHTKKKEVGVSDLTLLSTISDESINDNLKKRFENGIIYTYIGHVLISVNPFRDLGIY
NabMyo1B      1 -MAVIKRGVRTKPS---AGTVKKT-----------TKIKKATFDSSKQNEVGVSDLTLLSKISDDSINSNLKKRFQNGLIYTYIGHVLISVNPFRDLGIY
ZbMyo1        1 -MAIIKKGARTKTA---AAPQKRS-----------AKIKKAAFDTTKRKEVGVSDLTLLSTISDEAINDNLKKRFKNGSIYTYIGHVLISVNPFRDLGIY
Ca_bMyo1      1 -MAIVKRGGRTKTK-QQQVPAKSSGGGSS------GGIKKAEFDITKKKEVGVSDLTLLSKITDEAINENLHKRFMNDTIYTYIGHVLISVNPFRDLGIY
StaMyo1       1 -MGIVKRRGDKKN---QAPPAKSSGGGSG------GGIKKAEFDITKKKEVGVSDLTLLSKITDEAINDNLHKRFMNDTIYTYIGHVLISVNPFRDLGIY
ShpMyo1       1 -MAIVKLGGRKKNQ---APPAKSNGSGG-------GGIKKAEFDITKKKEVGVSDLTLLSKITDEAINDNLHKRFMNDTIYTYIGHVLISVNPFRDLGIY
CllMyo1       1 -MAIVKKGGRTRAK-QQAAPAK-------------SGIKKAEFDITKKKEVGVSDLTLLSKITDEAINENLHKRFMNGTIYTYIGHVLISVNPFQDLGIY
MefMyo1       1 -MAIVKRGARSKAK-QQAPPAK-------------SGIKKAEFDLTKKKEVGVSDLTLLSKISDDAINENLQKRFMNGSIYTYIGHVLISVNPFQDLGIY
CnmMyo1       1 -MAIVKRGGRTKTK-QQQAPARATAGG--------GGIKKAEFDITKKKEVGVSDLTLLSKITDEAINENLQKRFMNDTIYTYIGHVLISVNPFRDLGIY
DhhMyo1       1 -MAIVKRGVRTKNK-QSQQPSK-------------SGIKKAEFDLHKKKEVGVSDLTLLSKIADDAINDNLYKRFMNSTIYTYIGHVLISVNPFEDLGIY
DehMyo1       1 -MAIVKRGVRTKNK-QSQQPSK-------------SGIKKAEFDLHKKKEVGVSDLTLLSKIADDAINDNLYKRFMNSTIYTYIGHVLISVNPFEDLGIY
CatMyo1       1 -MAVVKRGGRKKDK-QQAPPAK-------------SGIKKAEFDITKKKEVGVSDLTLLSKITDDAINDNLHKRFMNNTIYTYIGHVLISVNPFQDLGIY
LoeMyo1       1 -MAIVKRGGRTRAK-QQQAPAKVNNGSGAGAGAG-GGIQKAEFDITKKKEVGVSDLTLLSKITDEAINENLHKRFMNDTIYTYIGHVLISVNPFRDLGIY
MrgMyo1       1 -MAIVKRGARSKAK-QEAPAKS--------------GIKKXAEFDLHKKKEVGVSDLTLLSKISDDSINDNLHKRFMNNTIYTYIGHVLISVNPFQDLGIY
ShsMyo1       1 -MAIVKRGGRTKNKTQQQAPAK-------------SGIKKAEFDLHKSKEVGVSDLTLLSKITDEAINDNLHKRFMNGTIYTYIGHVLISVNPFQDLGIY
CameMyo1      1 -MAIVKRGGRSKAK-QQQAPAKVNGSSG-------GGIKKAEFDITKKKEVGVSDLTLLSKITDEAINDNLHKRFMNDTIYTYIGHVLISVNPFRDLGIY
MifMyo1Alpha  1 -MAIVKRGGRSKAK-QQAPPTK-------------SGIKKAEFEFHKKKEVGVSDLTLLTKITDEAINENLQKRFMNDTIYTYIGHVLISVNPFRDLGIY
CadMyo1       1 -MAIVKRGGRTKTK-QQQVPAKSSGGASGG-----GGIKKAEFDITKKKEVGVSDLTLLSKITDEAINENLHKRFMNDTIYTYIGHVLISVNPFRDLGIY
CaoMyo1       1 -MAIVKRGGRSKAK-QQQAPAKVNGSSA-------GGIKKAEFDVTKKKEVGVSDLTLLSKITDEAINDNLHKRFMNDTIYTYIGHVLISVNPFRDLGIY
CapMyo1       1 -MAIVKRGGRSKAK-QQQAPAKSNGSSG-------GGIKKAEFDVTKKKEVGVSDLTLLSKITDEAINDNLHKRFMNDTIYTYIGHVLISVNPFRDLGIY
Ct_aMyo1      1 -MAIVKRGGRNKAK-QQQAPARANGGS--------GGIKKAEFDITKKKEVGVSDLTLLSKITDEAINENLQKRFMNDTIYTYIGHVLISVNPFRDLGIY
PtaMyo1       1 -MAITKRAGRTKRS-GATKTSAEPPAK--------RGIQKATFDASKKKEVGVSDLTLLSTVTDESINDNLEKRFKNNTIYTYIGNVLISVNPFKDLGIY
CacaMyo1B     1 -MAIIKRGVRSERT--RVPATKA-----------SGKIKKATFKGSDTKEAGVSDLTLLSKISDAAINENLKKRFFSGLIYTYIGHVLISVNPFKDLGIY
NadMyo1       1 -MGLIRRGVRNKTA---AEPVKRSAK-----------IKKATFDATKKKEVGVSDLTLLSKISDESINENLKKRFMNATIYTYIGHVLISVNPFRDLGIY
```

```
                          110       120       130       140       150       160       170       180       190       200
                  ....|....|....|....|....|....|....|....|....|....|....|....|....|....|....|....|....|....|....|....|
CnbMyo1A       86 TEAIMQSYQGKNRLEVPPHVYAIAESMYYNLKAYNENQCVIISGESGAGKTEAAKKIMEYIAATSSTHSESIG-------KIKDMVLATNPLLESFGCAK
CdnMyo1A       86 TDAIMQSYQGKNRLEVPPHVYAIAESMYYNLKAYNENQCVIISGESGAGKTEAAKKIMEYIAATSSTHSESIG-------KIKDMVLATNPLLESFGCAK
CacaMyo1A      86 TDAVMQSYQGRNRLEVPPHVFAIAESMYYNMKAYNENQCVIISGESGAGKTEAAKQIMQYIAASSTSSNSESIG------KIKDMVLATNPLLESFGCAK
NabMyo1A       86 TDAIMATYQGKNRLEVPPHVYAIAESMYYNMKAYNENQCVIISGESGAGKTEAAKRIMQYIAAASTSNSESIG-------KIKDMVLATNPLLESFGCAK
NdMyo1A        86 TDAIMQSYQGKNRLEVPPHVYAIAESMYYNLKAYNENQCVIISGESGAGKTEAAKKIMEYIAATSSTHSKSIG-------KIKDMVLATNPLLESFGCAK
CnbMyo1B       86 TDDVMESYQGKNRLEVPPHVFAIAESMYYNLKSYNENQCVIISGESGAGKTEAAKRIMQYIAATSSTHSESIG-------QIKNMVLATNPLLEAFGCAK
CglMyo1A       87 TDAIMKSYQGKNRLEVPPHVYAISEAMYYNLKAYNENQCVIISGESGAGKTEAAKKIMEYIAATSSTHSESIG-------KIKDMVLATNPLLESFGCAK
Sab_aMyo1B     86 TDAIMDEYKGKNRLEVPPHVFAIAESMYYNMKSYNENQCVIISGESGAGKTEAAKRIMQYIAAASSAHTESIG-------KIKDMVLATNPLLESFGCAK
Sc_cMyo1B      86 TDAVMNEYKGKNRLEVPPHVFAIAESMYYNMKSYNENQCVIISGESGAGKTEAAKRIMQYIAAASSTHTESIG-------KIKDMVLATNPLLESFGCAK
SaaMyo1B       86 TDAVMNEYKGKNRLEVPPHVYAIAESMYYNMKSYNENQCVIISGESGAGKTEAAKQIMQYIAAASSAHTESIG-------KIKDMVLATNPLLESFGCAK
SakMyo1B       86 TDAVMNEYKGKNRLEVPPHVFAIAESMYYNMKSYNENQCVIISGESGAGKTEAAKRIMQYIAAASSAHTESIG-------KIKDMVLSTNPLLESFGCAK
SmiMyo1B       86 TDAVMNEYKGKNRLEVPPHVFAIAESMYYNMKSYNENQCVIISGESGAGKTEAAKRIMQYIAAASSTHTESIG-------KIKDMVLATNPLLESFGCAK
Sap_aMyo1B     86 TDAVMNEYKGKNRLEVPPHVFAIAESMYYNMKSYNENQCVIISGESGAGKTEAAKRIMQYIAAASSTHTESIG-------KIKDMVLATNPLLESFGCAK
NacMyo1        86 TDAIVESYKGKNRLEVPPHVFAIAESMYYNMKSYNENQCVIISGESGAGKTEAAKRIMQYIASASTTHSESIG-------KIKDMVLATNPLLESFGCAK
KaaMyo1        86 TDATLEHYKGKNRLEVPPHVFAIAEAMYYNLKSYNENQCVIISGESGAGKTEAAKRIMQYIAAASTTHSDSIA-------KIKDMVLATNPLLESFGCAK
VpMyo1         86 TDAVLESYKGKNRLEVPPHVFAIAESMYYNLKAYNENQCVIISGESGAGKTEAAKRIMQYIASASESNSESIG-------KIKDMVLATNPLLESFGCAK
TtpMyo1A       86 TDAVLQSYKGKNRLEVPPHVYAIAESMYYNLKAYNESQCVIISGESGAGKTEAAKRIMQYIAAASDTNSESIS-------KIKDMVLATNPLLESFGCAK
TtpMyo1B       86 TDAVLQSYKGKXRLEVPPHVYAIAESMYYNLKAYNESQCVIISGESGAGKTEAAKRIMQYIAAASDTNSESIS-------KIKDMVLATNPLLESFGCAK
TtbMyo1A       88 SQEILESYKGKNRLEAPPHVFAIAEQMYYNLQAYNEGQCVIISGESGAGKTEAAKRIMQYVAHSSGSNSHSESIK-----RINDIVLATNPLLESFGCAK
TtbMyo1B       88 TDAVLQSYKGKNRLEAPPHVFAIAESMYYNMKSYNENQCVIISGESGAGKTEAAKRIMQYIAASSSSNSESIT-------KIKDIVLATNPLLESFGCAK
ErgMyo1        85 TDQVLETYKGRNRLEVPPHVFAIAEAMYYNLKAYNENQCVIISGESGAGKTEAAKRIMQYIAAASSSHEASIG-------RIKDMVLATNPLLESFGCAK
ErcMyo1        86 TDQVLATYKGKNRLEVPPHVFGIAESMYYNLRAYNENQCVIISGESGAGKTEAAKRIMQYIASVSSSHEQSIG-------KIKDMVLATNPLLESFGCAK
HsvMyo1        87 TDAVLQSYKGKNRLEVPPHVFAIAESMYYNLKAYNENQSVIISGESGAGKTEAAKRIMQYIAAASSSQQDSIT-------KIKDMVLATNPLLESFGCAK
KlMyo1         84 TDAVLESYKGKNRLEVPPHVFAIAEAMYYNLKAYNENQCVIISGESGAGKTEAAKRIMQYIAAASSTHEASIG-------KIKDMVLATNPLLESFGCAK
KmmMyo1        84 TDAVLQSYKGKNRLEVPPHVFAIAEAMYYNLKAYNENQCVIISGESGAGKTEAAKRIMQYIAAASDTHEASIG-------KIKDMVLATNPLLESFGCAK
KlwMyo1        84 TDAVLQSYKGKNRLEVPPHVFAIAEAMYYNLKAYNENQCVIISGESGAGKTEAAKRIMQYIAAASSSHEASIG-------KIKDMVLATNPLLESFGCAK
KaMyo1         85 TDAVLQSYKGKNRLEVSPHVFAIAESMYYNLKAYNENQCVIISGESGAGKTEAAKRIMQYIAAASSTHEASIG-------KIKDMVLATNPLLESFGCAK
KnMyo1         86 TAAVLESYRGKNRLEVPPHVFAIAEAMYYNMKSYNTNQCVIISGESGAGKTEAAKRIMQYIAAASTSHSESIG-------KIKDMVLATNPLLEAFGCAK
LwMyo1         86 TDSVLRSYVGKNRLEVAPHVFAIAESMYYNMKAYNENQCVIISGESGAGKTEAAKRIMQYIAAASETHSDSIG-------KIKDMVLATNPLLESFGCAK
LatMyo1        86 TDSVLKSYMGKNRLEVPPHVFAIAESMYYNMKAYNENQCVIISGESGAGKTEAAKRIMQYIAAASNTHSDSIG-------KIKDMVLATNPLLESFGCAK
Lak_aMyo1      90 TDQVLESYKGKNRLEVPPHVFAIAEAMYYNLKAYNENQCVIISGESGAGKTEAAKRIMQYIAAASSTHSESIG-------KIKDMVLATNPLLESFGCAK
Sab_aMyo1A     86 TDSVLESYKGKNRLEVPPHVFAIAESMYYNLKSYNENQCVIISGESGAGKTEAAKRIMQYIAAASSSHSESIG-------KIKDMVLATNPLLESFGCAK
Sc_cMyo1A      86 TNAVLESYKGKNRLEVPPHVFAIAESMYYNLKSYNENQCVIISGESGAGKTEAAKRIMQYIAAASNSHSESIG-------KIKDMVLATNPLLESFGCAK
SaaMyo1A       86 TEAVLESYKGKNRLEVPPHVFAIAESMYYNLKSYNENQCVIISGESGAGKTEAAKRIMQYIASASNSHSESIG-------KIKDMVLATNPLLESFGCAT
SakMyo1A       86 TDAVLESYKGKNRLEVPPHVFAIAESMYYNLKSYNENQCVIISGESGAGKTEAAKRIMQYIAAASNSHSESIG-------RIKDMVLATNPLLESFGCAT
SmiMyo1A       86 TNTVLETYKGKNRLEVPPHVFGIAESMYYNLKSYNENQCVIISGESGAGKTEAAKRIMQYIAATSNSHSESIG-------KIKDMVLATNPLLESFGCAK
Sap_aMyo1A     86 TNAVLESYKGKNRLEVPPHVFAIAESMYYNLKSYNENQCVIISGESGAGKTEAAKRIMQYIAAASNSHSESIG-------KIKDMVLATNPLLESFGCAK
ZrMyo1         86 TDAVLESYRGKNRLEAPPHVFAISESMYYNMKAYNENQCVIISGESGAGKTEAAKRIMQYIAAASTTHTESIG-------KIKDMVLATNPLLESFGCAK
TodMyo1        86 TDAILESYKGKNRLEVPPHVFAIAESMYYNMKAYNENQCVIISGESGAGKTEAAKRIMQYIAAASTNHSESIG-------KIKDMVLATNPLLESFGCAK
CglMyo1B       86 TDDTMKSYQGKNRLEAPPHVFAIAENMYYNLKSYNENQCVIISGESGAGKTEAAKRIMQYIAATSSTHSESIS-------KIKDMVLATNPLLESFGCAK
WaMyo1Alpha    84 TDKVLESYEGKNRLEVPPHVFAIAESMFYNMKAYNESQCVIISGESGAGKTEAAKRIMQYVAAVSGSSSSSQQIQ-----QIKDIVLATNPLLESFGCAK
WicMyo1        84 TDQVLSTYQGKNRLEVPPHVFAIAESMFYNLKAYNESQCVIISGESGAGKTEAAKRIMQYIASVSGSNNSSKQIQ-----QIKDIVLATNPLLESFGCAK
CyjMyo1        84 TEDVLKSYEGKNRLEVPPHVFAVAESMVYNMKAYNESQCVIISGESGAGKTEAAKRIMQYIAAATATSVSSKSIT-----EIRDIVLATNPLLESFGCAK
YlMyo1         87 TDAVLESYKGKNRLEVPPHVFSIAESMYYNMKSYSENQCVIISGESGAGKTEASKRIMQYIASASGGSSSNIQ-------KIKDMVLATNPLLEAFGCAK
CdnMyo1B       86 TDEVMQSYQGKNRLEAPPHVFAIAESMYYNLKSYNENQCVIISGESGAGKTEAAKRIMQYIAATSSTHSESIG-------KIKDMVLATNPLLEAFGCAK
Kop_bMyo1      86 TDAILNSYRGKNRLEVPPHVFAIAESMFYNMQAYAESQCVIISGESGAGKTEAAKQIMQYIAAVSGGQSSSQIQ------QIKDMVLATNPLLESFGCAK
NdMyo1B        85 TDEIMRSYQGKNRLEAPPHVFAIAESMYYNMKSYNENQCVIISGESGAGKTEAAKQIMQYIAAMSSTHSESIG-------KIKDMVLATNPLLEAFGCAK
KcMyo1         88 SDAVLESYRGKNRLEVPPHVFAIAESMYYNMRAYDENQCVIISGESGAGKTEAAKRIMQYVAAVSGGTSSSIQ-------QIKDMVLATNPLLESFGCAK
OgpMyo1        90 TDEVLNSYKNKNRLEVPPHVFAIAESMYYNMRAYNENQCVIISGESGAGKTEAAKRIMQYIAAVSGSYTSSSHIQ-----RIKDMVLATNPLLESFGCAK
DebMyo1        89 TQEVIDSYKNTNRLEVPPHVYAIAESMYHNMVAYSESQCVIISGESGAGKTEAAKRIMQYIANISGSMTTSAEIK-----HINDMVLATNPLLESFGCAK
PiuMyo1        90 TLETLLSYKNKNRLEVPPHVYAIAENMYHNMMAYNDNQCVIISGESGAGKTEAAKRIMQYIAQVSGSLTNSTQIQ-----RINDMVLATNPLLESFGCAK
NabMyo1B       86 TTEIMGKYKGKNRLEVPPHVFAIAESMYYNMKSYNENQCVIISGESGAGKTEAAKRIMQYIAAVSSSHSQSIS-------KIKDMVLATNPLLESFGCAK
ZbMyo1         86 TDAVLESYRGKNRLEAPPHVFAISEAMYYNMKAYNENQCVIISGESGAGKTEAAKRIMQYIAAASSTHTQSIG-------KIKDMVLATNPLLESFGCAK
Ca_bMyo1       93 TLENLNKYKGRNRLEVPPHVFAIAESMYYNLKSYGENQCVIISGESGAGKTEAAKQIMQYIANVSVDQDNVEIS------KIKDMVLATNPLLESFGCAK
StaMyo1        91 TDEHLNKYRGRNRLEVPPHVFAIAESMYYHLKSYSENQCVIISGESGAGKTEAAKQIMQYIANVSVDKGNAEIS------KIKDMVLATNPLLESFGCAK
ShpMyo1        90 TPEHLDKYRGRNRLEVPPHVFAIAESMYYHLKSYGENQCVIISGESGAGKTEAAKQIMQYIANVSVDKGNVEIT------KIKDMVLATNPLLESFGCAK
CllMyo1        86 THETLNMYKGKNRLEVPPHVYAIAESMYYNLKSYGDNQCVIISGESGAGKTEAAKQIMQYIANVSVDDQLSSTSDSSGISKIKDMVLATNPLLESFGCAK
MefMyo1        86 TPQTLALYKGKNRLEVPPHVYAIAETMYYHLKSYGENQCVIISGESGAGKTEAAKQIMQYIANVSVDDQIGSGSGGGGIAKIKDMVLATNPLLESFGCAK
CnmMyo1        91 TMDNLNKYKGRNRLEVPPHVFAIAESMYYNLKSYGENQCVIISGESGAGKTEAAKQIMQYIANVSVGTENDEIS------KIKDMVLATNPLLESFGCAK
DhhMyo1        86 TLENLNKYKGKNRLEVPPHVFAIAESMYYNLKSYGENQCVIISGESGAGKTEAAKQIMQYIANVSVDDQMGAGGDGDIS-KIKDMVLATNPLLESFGCAK
DehMyo1        86 TPEHLNKYKGKNRLEVPPHVFAIAESMYYNLKSYGDNQCVIISGESGAGKTEAAKQIMQYIANVSVDDQVSAGGDGGIS-KIKDMVLATNPLLESFGCAK
CatMyo1        86 TQENLNKYKGKNRLEVPPHVFAIAETMYYNLKSYGENQCVIISGESGAGKTEAAKQIMQYIANVSVEDHGKALANNDIL-KIKDMVLATNPLLESFGCAK
LoeMyo1        98 TLENLNKYKGRNRLEVPPHVFAIAESMYYHLKSYGENQCVIISGESGAGKTEAAKQIMQYIANVSVDSGNAEIS------KIKDMVLATNPLLESFGCAK
MrgMyo1        86 TKEYLNMYKGKNRLEVPPHVFAIAESMYYHLKSYGESQCVIISGESGAGKTEAAKQIMQYIANVSVDDKVSTTSEIT---QIKDMVLATNPLLESFGCAK
ShsMyo1        87 GPETLSKYRGRNRLEVPPHVFAIAESMYYNLKSYNENQCVIISGESGAGKTEAAKQIMQYIANVSVDSGNAEIS------KIKDMVLATNPLLESFGCAK
CameMyo1       92 TMETLNKYKGRNRLEVPPHVFAIAESMYYHLKSYGENQCVIISGESGAGKTEAAKQIMQYIANVSVDTGNAEIS------KIKDMVLATNPLLESFGCAK
MifMyo1Alpha   86 TTSTLNEYKGRNRLEVPPHVFAIAESMYYNLKSYGDNQCVIISGESGAGKTEAAKQIMQYIAYVSVDDQAGANSEGIS--KIKDMVLATNPLLESFGCAK
CadMyo1        94 TLENLNKYKGRNRLEVPPHVFAIAESMYYNLKSYGENQCVIISGESGAGKTEAAKQIMQYIANVSVDQDNIEIS------KIKNMVLATNPLLESFGCAK
CaoMyo1        92 TMETLNKYKGRNRLEVPPHVFAIAESMYYHLKSYGENQCVIISGESGAGKTEAAKQIMQYIANVSVDSGNAEIT------KIKDMVLATNPLLESFGCAK
CapMyo1        92 TSETLNKYKGRNRLEVPPHVFAIAESMYYHLKSYGENQCVIISGESGAGKTEAAKQIMQYIANVSVDTGNAEIT------KIKDMVLATNPLLESFGCAK
Ct_aMyo1       91 TMDNLNKYKGRNRLEVPPHVFAIAESMYYNLKSYGENQCVIISGESGAGKTEAAKQIMQYIANVSVDAQNAEIS------KIKDMVLATNPLLESFGCAK
PtaMyo1        91 TDKVLESYRGKNRLEVQPHVFAIAESMYYQMKSYSENQCVIISGESGAGKTEAAKRIMQYIAEVSGGSNSTEIQ------KIKDMVLATNPLLESFGCAK
CacaMyo1B      87 TQAVMDSYKGKNRLERPPHVFAIAESMFYNMKFYGENQCVIISGESGAGKTEAAKQIMQYIAAVSSSTSDSQSIG-----RIKDMVLATNPLLESFGCAK
NadMyo1        86 TDAILDSYKGKNRLEVPPHVFAIAESMYYNLKSYNESQCVIISGESGAGKTEAAKRIMQYIASAS-TSHSESIG------KIKDMVLATNPLLESFGCAK
```

```
                               210       220       230       240       250       260       270       280       290       300
                      ....|....|....|....|....|....|....|....|....|....|....|....|....|....|....|....|....|....|....|....|
CnbMyo1A        179  TLRNNNSSRHGKYLEIKFNSQFE-PCAGNITNYLLEKQRVVSQITNERNFHIFYQFTKGAPESYRQSFGVQLPEQYVYTSASKCTSVDTIDDVKDFQETL
CdnMyo1A        179  TLRNNNSSRHGKYLEIRFNAQFE-PCAGNITNYLLEKQRVVSQITNERNFHIFYQFTKGAPENYRQLFGVQLPEQYVYTSASKCTSVDTIDDVKDFQDTI
CacaMyo1A       180  TLRNNNSSRHGKYLEIKFNAQFE-PCAGNITNYLLEKQRVVSQIKNERNFHIFYQLTKGASDNYRQIFGIQQPEQYIYTAASQCTTVDTIDDIKDWEGTL
NabMyo1A        179  TLRNNNSSRHGKYLEIRFNAQYE-PCAGNITNYLLEKQRVVSQIQNERNFHIFYQFTKGASESYRQTFGVQPPEQYVYTAAAKCTSVDNIDDVTDYQETL
NdMyo1A         179  TLRNNNSSRHGKYLEIRFNAQFE-PCAGNITNYLLEKQRVVSQITNERNFHIFYQFTKGAPENYRQSFGVQLPEQYVYTSASKCTSVDTIDDVKDFQDTI
CnbMyo1B        179  TLRNNNSSRHGKYLEIKFNAQFE-PCAGNITNYLLEKQRVVGQIKNERNFHIFYQFTKGASDEYRQLFGVQQPEQYIYTSASQCTKVDNMDDVKEFQETL
CglMyo1A        180  TLRNNNSSRHGKYLEIRFNSQFE-PCAGNITNYLLEKQRVVGQITNERNFHIFYQFTKGASDNYRQTFGVQLPEQYVYTSASKCTSVDTIDDVKDFEATI
Sab_aMyo1B      179  TLRNNNSSRHGKYLEIKFNNQFE-PCAGNITNYLLEKQRVVSQIKNERNFHIFYQFTKGASDAYRQTFGVQKPEQYVYTAAAGCISADTIDDLQDYQETL
Sc_cMyo1B       179  TLRNNNSSRHGKYLEIKFNNQFE-PCAGNITNYLLEKQRVVSQIKNERNFHIFYQFTKGASDAYRQTFGVQKPEQYVYTAAAGCISAETIDDLQDYQETL
SaaMyo1B        179  TLRNNNSSRHGKYLEIKFNSQFE-PCAGNITNYLLEKQRVVSQIKNERNFHIFYQFTKGASDAYRQTFGVQKPEQYVYTAAAGCISADTIDDLQDYQETL
SakMyo1B        179  TLRNNNSSRHGKYLEIKFNNQFE-PCAGNITNYLLEKQRVVSQIKNERNFHIFYQFTKGASDTYRQTFGVQKPEQYVYTAAAGCISADTIDDLQDYQETL
SmiMyo1B        179  TLRNNNSSRHGKYLEIKFNNQFE-PCAGNITNYLLEKQRVVSQIKNERNFHIFYQFTKGASDAYRQTFGVQKPEQYVYTAAAGCISADTIDDLQDYQETL
Sap_aMyo1B      179  TLRNNNSSRHGKYLEIKFNNQFE-PCAGNITNYLLEKQRVVSQIKNERNFHIFYQFTKGASDAYRQTFGVQKPEQYVYTAAAGCISAETIDDLQDYQETL
NacMyo1         179  TLRNNNSSRHGKYLEIRFNSQFE-PCAGNITNYLLEKQRVVSQIKNERNFHIFYQFTKGASDAYRQTFGVQKPEQYIYTSASGCTSVDTIDDVNDYRETL
KaaMyo1         179  TLRNNNSSRHGKYLEIKFNSQFE-PCAGNITNYLLEKQRVVGQIKNERNFHIFYQFTKGASENYRQMFGVQQPDQYIYTSASGCISVENMDDVKDFAETI
VpMyo1          179  TLRNNNSSRHGKYLEIRFNNQFE-PCAGNITNYLLEKQRVVSQIKEERNFHIFYQFTKGASENYRQTFGVQKPEQYIYTSAAGCTSVDTIDDVKDYEDTL
TtpMyo1A        179  TLRNNNSSRHGKYLEIKFNAQYE-PCAGNITNYLLEKQRVVSQIKNERNFHIFYQFTKGASENYRQTFGVQKPEQYVYTSAAGCTSVDTIDDIKDFADTI
TtpMyo1B        179  TLRNNNSSRHGKYLEIKFNAQYE-PCAGNITNYLLEKQRVVSQIKNERNFHIFYQFTKGASENYRQTFGVQKPEQYVYTSAAGCTSVDTIDDIKDFADTI
TtbMyo1A        183  TLRNNNSSRHGKYLEIKFNKFFE-PCAGNITNYLLEKQRVVSQIRDERNFHIFYQYTKGASETYRQTFGVQLPEQYLYTSASGCTEVNGINDVNEFAETI
TtbMyo1B        181  TLRNNNSSRHGKYLEIKFNSQFE-PCAGNITNYLLEKQRVVSQIKNERNFHIFYQFTKAAPESYRQAFGIQTPDRYIYTSASGCISVDTIDDVKDFQDTL
ErgMyo1         178  TLRNNNSSRHGKYLEIRFNAQFE-PCAGQITNYLLEKQRVVGQIKNERNFHIFYQFSKGASDRYRKTYGVQLPEQYVYTSASGCTSVDTIDDLNDYEATL
ErcMyo1         179  TLRNNNSSRHGKYLEIRFNSQFE-PCAGNITNYLLEKQRVVSQIKNERNFHIFYQFTRGASDTYRKIYGVQLPEHYIYTSASGCTSVDTIDDLEDYKETL
HsvMyo1         180  TLRNNNSSRHGKYLEIKFNATFD-PISANITNYLLEKQRVVSQIKNERNFHIFYQFTKGASDNYRQIYGVQKPEQYVYTA--TCTTADTIDDLKDWQDTL
KlMyo1          177  TLRNNNSSRHGKYLEIRFNEQFE-PCAGQITNYLLEKQRVVGQIRNERNFHIFYQFTKGASDTYRQNFGVLQPDQYIYTSASGCTSVDTIDDLHDYQETI
KmmMyo1         177  TLRNNNSSRHGKYLEIRFNEQFE-PCAGQITNYLLEKQRVVGQIKNERNFHIFYQFTKGASDTYRQNFGVLQPDQYVYTSASGCTSVDTIDDLDDFKETL
KlwMyo1         177  TLRNNNSSRHGKYLEIRFNEQFE-PCAGQITNYLLEKQRVVGQIKNERNFHIFYQFTKGASDVYRQNFGVLQPDQYIYTSASGCTSVDTIDDLDDFKETL
KaMyo1          178  TLRNNNSSRHGKYLEIRFNEKFE-PCAGQITNYLLEKQRVVGQIKNERNFHIFYQFTKGAPDSYRQNFGVLQPDQYIYTSASGCTSVDTIDDLKDFNETL
KnMyo1          179  TLRNNNSSRHGKYLEIKFNSQFE-PCAGNITNYLLEKQRVVSQIKNERNFHIFYQFTKGASDAYRQTFGVQKPEQYIYTSASGCTSVDNMDDVKEFGETL
LwMyo1          179  TLRNNNSSRHGKYLEIRFNAQFE-PCAGNITNYLLEKQRVVGQLKDERNFHIFYQFTKGASDNYRQTFGVQKPEQYLYTSASGCTSVDTIDDLSDFQETL
LatMyo1         179  TLRNNNSSRHGKYLEIRFNAQFE-PCAGNITNYLLEKQRVVGQLKDERNFHIFYQFTKGASDNYRQLFGVQMPEQYIYTSASGCTSVDTIDDLSDFQATL
Lak_aMyo1       183  TLRNNNSSRHGKYLEIRFNSRFE-PCAGNITNYLLEKQRVVGQIQNERNFHIFYQFTKGASESYRQLFGVQLPEQYIYTSASGCTSVDTIDDTKDYQETL
Sab_aMyo1A      179  TLRNNNSSRHGKYLEIKFNSQFE-PCAGNITNYLLEKQRVVSQIKHERNFHIFYQFTKGASDAYRQTFGVQMPEQYIYTAAAGCTSADTIDDVKDYQGTL
Sc_cMyo1A       179  TLRNNNSSRHGKYLEIKFNSQFE-PCAGNITNYLLEKQRVVGQIKNERNFHIFYQFTKGASDTYKQMFGVQMPEQYIYTAAAGCTSADQLMR-KDYEGTL
SaaMyo1A        179  TLRNNNSSRHGKYLEIKFNSQFE-PCAGNITNYLLEKQRVVSQIKNERNFHIFYQFTKGASDAYRQTFGVQVPEQYVYTAAAGCTTVDTIDDIKDYQGTL
SakMyo1A        179  TLRNNNSSRHGKYLEIKFNSQFE-PCAGNITNYLLEKQRVVSQIKNERNFHIFYQFTKGASDVYRQTFGVQMPEQYIYTAAAGCTTADTID---------
SmiMyo1A        179  TLRNNNSSRHGKYLEIKFNSQFE-PCAGNITNYLLEKQRVVSQIKNERNFHIFYQFTKGASDAYRQTFGVKMPEEYIYTAAAGCTTADTIDDIKDYQGTI
Sap_aMyo1A      179  TLRNNNSSRHGKYLEIKFNSQFE-PCAGNITNYLLEKQRVVSQIKNERNFHIFYQFTKGASDTYRQMLGVQMPEQYIYTAAAGCTTADTIDDIKDFQGTL
ZrMyo1          179  TLRNNNSSRHGKYLEIRFNSLFE-PIAGNITNYLLEKQRVVGQIKNERNFHIFYQYTKGATENYRQLFGVQSPEQYVYTSRAGCTSVEGIDDTKDFQATL
TodMyo1         179  TLRNNNSSRHGKYLEIRFNAQFE-PCAGNITNYLLEKQRVVSQIKNERNFHIFYQFTKGASENYRQTFGVQKPEQYIYTSASGCTSVDTVDDLKDYQATL
CglMyo1B        179  TLRNNNSSRHGKYLEIKFDAHFQ-PCAGHITNYLLEKQRVVGQIKNERNFHIFYQFTKGAPEEYRQLFGVQQPEQYIYTSASQCTAVENMDDVEEFNETL
WaMyo1Alpha     179  TLRNNNSSRHGKYLEINFNAQFE-PVSGNITNYLLEKQRVVGQIKNERNFHIFYQFTKGASENYRQTFGIQEPDRYIYTSSAGCISVDSIDDLKDFQDTL
WicMyo1         179  TLRNNNSSRHGKYLEIQFNNQFE-PVSGNITNYLLEKQRVVGQIQNERNFHIFYQFTKGASENYRQIFGVQQPDHYIYTSRAKCITVDSIDDLKDFNDTL
CyjMyo1         179  TLRNNNSSRHGKYLEINFSEQYE-PVSGKITNYLLEKQRVVGQNKGERNFHIFYQFTKAAPESYRQTFGVQGPEQYLYTSG--TTSVEGIDDAKDFQETL
YlMyo1          180  TLRNDNSSRHGKYLEIQFNNQAE-PVGANITNYLLEKGRVVGQIRNERNFHIFYQFAKAASENYRSTFGIQPPEAYTYTSASGCTSVNGINDEAEFKETL
CdnMyo1B        179  TLRNNNSSRHGKYLEIKFNAQFE-PCAGNITNYLLEKQRVVGQIKNERNFHIFYQLTKGATDEYRQLFGIQAPEQYIYTSASQCTSVENMDDVKEFQETL
Kop_bMyo1       180  TLRNNNSSRHGKYLEIQFNAGYE-PIGAHITNYLLEKQRVVGQIRNERNFHIFYQFTKSANENYRSTFGVQLPEQYVYTSASGCTTVDTIDDVKEYKDTL
NdMyo1B         178  TLRNNNSSRHGKYLEIKFNEQFE-PCAGNITNYLLEKQRVVGQIRNERNFHIFYQFTKGAKDEYRQLYGVRTPENYIYTSASQCTTVENMDDVKEFQETL
KcMyo1          181  TLRNDNSSRHGKYLEIQFNKLYE-PVSAHITNYLLEKQRVVGQIKNERNFHIFYQFTKAASENYRTTYGVQQPEQYVYTSASGCTSVSSIDDPKDFEDTL
OgpMyo1         185  TLRNNNSSRHGKYLEIQFNKSFE-PVSANITNYLLEKQRVVGQIKNERNFHIFYQYTKGASDQYRQTFGVQNPEQYVYTSASGCTSVDSIDDLKDFKDTL
DebMyo1         184  TLRNDNSSRHGKYLEINFNKAFE-PVSANITNYLLEKQRVVGQIQNERNFHIFYQFTKGASDKYRQTYGVQQPEQYLYTSASGCTSVDSIDDLANFNDTL
PiuMyo1         185  TLRNNNSSRHGKYLEIQFNKSFE-PVSANITNYLLEKQRVVGQIQNERNFHIFYQLTKAADDNYRQIFGVQKPEQYVYTAQSGCTSVDSIDDVHDYTETL
NabMyo1B        179  TLRNNNSSRHGKYLEIRFNAQYE-PCAGNITNYLLEKQRVVSQIENERNFHIFYQFTKGTSDHYRQLYGVQEPEKYIYTGASKCVTVDKIDDLADYQETL
ZbMyo1          179  TLRNNNSSRHGKYLEIRFNSLFE-PIAGNITNYLLEKQRVVSQIKNERNFHIFYQFTKGASENYRQIYGVQKPEQYVYTSKAGCTTVDTIDDKKDFQATL
Ca_bMyo1        187  TLRNNNSSRHGKYLEIKFSEGNYQPIAAHITNYLLEKQRVVSQITNERNFHIFYQFTKHCPPQYQQMFGIQGPETYVYTSAAKCINVDGVDDAKDFQDTL
StaMyo1         185  TLRNNNSSRHGKYLEIQFSEGSYQPIAAHITNYLLEKQRVVSQITNERNFHIFYQFTKACPEKYKQMFGIQGPESYIYTAAAKCIDVEGINDRNDFQDTI
ShpMyo1         184  TLRNNNSSRHGKYLEIQFSEGSYQPIAAHITNYLLEKQRVVSQITNERNFHIFYQFTKACPEKYKQQFGIQGPESYIYTAAAKCIDVDGINDSKDFNDTI
CllMyo1         186  TLRNNNSSRHGKYLEIFFDPQSHRPVAAHITNYLLEKQRVVQQITNERNFHIFYQFTKHCPPQYAQMFGIQGPETYIYTSAAKCVSADGIDDGKDFQETI
MefMyo1         186  TLRNNNSSRHGKYLEIYFNPENQ-PSAAHITNYLLEKQRVVQQISNERNFHIFYQFTKHCPPQYQQAYGVQGPESYVYTSAAKCINADGIDDAKDFQETI
CnmMyo1         185  TLRNNNSSRHGKYLEITFNEKNYQPTAAHITNYLLEKQRVVSQITNERNFHIFYQFTKNCPPQYQQAFGIQGPETYIYTSAAKCINVDGVDDTKDFKDTL
DhhMyo1         185  TLRNNNSSRHGKYLEIYFNPSNYQPVAAHITNYLLEKQRVVSQITNERNFHIFYQFTKHCPPQYQQSYGIQGPETYIYTSAAKCINVDGVDDSKDLQDTL
DehMyo1         185  TLRNNNSSRHGKYLEIYFNPSNYQPVSAHITNYLLEKQRVVSQITNERNFHIFYQFTKHCPPQYQQSFGIQGPETYVYTSAAKCINVDGVDDSKDLQDTL
CatMyo1         185  TLRNNNSSRHGKYLEIYFNPSNYQPVAAHITNYLLEKQRVVSQITNERNFHIFYQFTKHCPDDYKQKFGIQGPETYIYTSAAGCVSVDGVNDSSDFQETL
LoeMyo1         192  TLRNNNSSRHGKYLEIKFSEGSYQPIAAHITNYLLEKQRVVSQITNERNFHIFYQFTKHCPPQYQQQFGIQGPETYVYTSAAKCITVDGIDDGKDFKDTL
MrgMyo1         183  TLRNNNSSRHGKYLEIYFNPSNYQPVAAHITNYLLEKQRVVSQITNERNFHIFYQLTKSCPPEYKQSFGLQGPETYVYTSAAKCIDVEGINDGKDFAETL
ShsMyo1         181  TLRNNNSSRHGKYLEIQFSESNHQPIAAHITNYLLEKQRVVSQITNERNFHIFYQFTKHCPTQYQQQFGIQGPETYVYTSSAKCINVDGIDDSKDLDETL
CameMyo1        186  TLRNNNSSRHGKYLEIKFSEGSYQPVAAHITNYLLEKQRVVSQITNERNFHIFYQFTKHCPPQYQQQFGISGPETYVYTSAAKCISVDGIDDGKDFKDTL
MifMyo1Alpha    184  TLRNNNSSRHGKYLEIYFDPSTYHPSAAHITNYLLEKQRIVSQITNERNFHIFYQFTKNCPPQYQQAYGIQGPESYIYTSAARCIDVDGINDSKDFNDTL
CadMyo1         188  TLRNNNSSRHGKYLEIKFNENNYQPIAAHITNYLLEKQRVVSQITNERNFHIFYQFTKNCPPQYQQMFGIQSPETYVYTSAAKCINVDGIDDAQDFQDTL
CaoMyo1         186  TLRNNNSSRHGKYLEIKFSEGSYQPVAAHITNYLLEKQRVVSQITNERNFHIFYQFTKHCPPQYQQQFGIQGPETYVYTSAAKCISVDGIDDGKDFKDTL
CapMyo1         186  TLRNNNSSRHGKYLEIKFSEGSYQPVAAHITNYLLEKQRVVSQISNERNFHIFYQFTKHCPPQYQQQFGIQGPETYVYTSAAKCITVDGIDDGKDFKDTL
Ct_aMyo1        185  TLRNNNSSRHGKYLEIMFNETYQ-PVAAHITNYLLEKQRVVSQITNERNFHIFYQFTKNCPPQYRESFGIQGPETYIYTSAAKCINVDGVDDTKDFQDTL
PtaMyo1         185  TLRNNNSSRHGKYLEIFFNSQDE-PIAGNITNYLLEKNRVVQQLNNERNYHIFYQLTK--SEKYRQVFGVQPPEKYQYTYGAKCTSVNGIDDLKDFDATL
CacaMyo1B       182  TLRNNNSSRHGKYLEIRFNSMDV-PCAGHITNYLLEKQRVVSQIKNERNFHIFYQLTKGATDHYRHIFGIQEPEKYIYTAASNCTSVDTLDDIKDFEGTI
NadMyo1         179  TLRNNNSSRHGKYLEIKFNSQFE-PCAGNITNYLLEKQRVVGQIKNERNFHIFYQFTKGASDTYRQTFGVQKPEQYIYTSASGCTSVDTIDDLKDYQETL
```

```
                       310       320       330       340       350       360       370       380       390       400
CnbMyo1A      278  KAMQIIGLSQDEQDQIFRMLAAILWIGNISFIENEEGNAQVRDTSVTDFVAYLLQID-SPSLTKSLVERIVETNHGMKRGSVYHVPLNIVQATAVRDALA
CdnMyo1A      278  KAMQVIGLSQEEQDQIFRMLASILWIGNISFIENEEGNAQVRDTSVTDFVAYLLQID-SPSLTKALVERIVETNHGMKRGSVYHVPLNIVQATAVRDALA
CacaMyo1A     279  KAARTIGLSQEEQDQIFRQLAAILWIGNLTFVENEEGNAQVRDTSVSDFIAYLLQVE-SELLIKCIVERTVETNHGMKRGSVYHVPLNIVQANAVRDALA
NabMyo1A      278  KAMQTIGLAQEEQDNIFRMLAAILWIGNISFIENEEGNAQIRDTSVTDFVAYLLQIN-SDILITAITERTVETNHGMKRGSVYHIPMNIVQATAVRDALA
NdMyo1A       278  KAMQIIGLSQDEQDQIFRMLASILWIGNISFIENEEGNAQVRDTSVTDFVAYLLQVD-SPSLTKALVERIVETSHGMKRGSVYHVPLNIVQATAVRDALA
CnbMyo1B      278  KAMEIIGLSKEEQDQIFRMLAVILWIGNISFIENEEGNAQIRDTSVTAFVAYLLQVQ-EELLIKSLIERIIETGHGAKRGSTYHSPLNIVQATAVRDALA
CglMyo1A      279  KAMQVIGLAQEEQDQIFRMLAAILWIGNISFIENEEGNAQVRDTSVTDFVAYLLQVD-SQSLIKALVERIVETNHGSRRGSVYHVPLNIVQATAVRDALA
Sab_aMyo1B    278  KAMKVIGLGQEEQDQIFRMLAAILWIGNISFIENEEGNAQVRDTSVTDFVAYLLQID-GQLLVKSLVERIMETNHGMKRGSVYHVPLNIVQADAVRDALA
Sc_cMyo1B     278  KAMRVIGLGQEEQDQIFRMLAAILWIGNVSFIENEEGNAQVRDTSVTDFVAYLLQID-SQLLIKSLVERIMETNHGMKRGSVYHVPLNIVQADAVRDALA
SaaMyo1B      278  KAMRVIGLEQDEQDQIFRMLAAILWIGNVSFIENEEGNAQVRDTSVTDFVAYLLQID-SQLLLKSLVERIMETSHGMKRGSVYHVPLNVVQADAVRDALA
SakMyo1B      278  KAMRVIGLGQEEQDQIFRMLAAILWIGNVSFTENEEGNSQVRDTSVTDFVAYLLQID-SQLLIKSLVERIMETNHGMKRGSVYHVPLNIVQADAVRDALA
SmiMyo1B      278  KAMRVIGLGQEEQDQIFRMLAAILWIGNVSFIENEEGNAQVRDTSVTDFVAYLLQID-SQLLIKSLVERIMETNHGMKRGSVYHVPLNVVQADAVRDALA
Sap_aMyo1B    278  KAMRVIGLGQEEQDQIFRMLAAILWIGNVSFIENEEGNAQVRDTSVTDFVAYLLQID-SQLLTKSLVERIMETNHGMKRGSVYHVPLNIVQADAVRDALA
NacMyo1       278  KAMEVIGLHQEEQDQIFRMLAAVLWIGNVSFVENEEGNAQVRDTSVTDFVAYLLQID-APLLIKSLVERVMETNHGMRRGSVYHVPLNIVQATAVRDALA
KaaMyo1       278  KAMQVIGLTQQEQDEIFRMLSAILWIGNITFIENEEGNAQVRDTSVTDFVAYLLQVD-SQLLIKSIVERTMETNHGMRRGSIYHVPLNIVQATAVKDALA
VpMyo1        278  KAMQVIGLSQDEQDQIFRMLSSILWVGNISFVENEEGNAQVRDTSVTDFVAYLLQID-SQVLMKALVERTMETSHGMRRGSVYHVPLNIVQATAVRDALA
TtpMyo1A      278  KAMQIIGLSQDEQDQIFRMLASILWIGNISFVENEEGNAQVRDTSVTDFVAYLLQID-ASILIKGLVERTMETSHGMRRGSVYHVPLNIVQANAAKDALA
TtpMyo1B      278  KAMQIIGLSQDEQDQIFRMLAFILWIGNISFVENEEGNAQVRDTSVTDFVAYLLQID-ASILIKGLVERTMETSHGMRRGSVYHVPLNIVQANAAEDALA
TtbMyo1A      282  KAMEIIGLDQNEQDQIFRMLAAILWIGNITFEENDEGNAQVRDTSVTDFVAYLLEVD-SPLLIKSLVERIMETSHGSQRGSVYHVPLNITQATAVRDALA
TtbMyo1B      280  KAMQVIGLSQDEQDQIFRMLSAILWIGNITFVENDEGNAQVADSSVTDFVAYLLQVD-AGVLVKSLVERIMETNHGMKRGSVYHVPLNRVQATAVRDALA
ErgMyo1       277  EAMNVIGLSQAEQDEIFRLLSAILWIGNVTFMEDDEGNAKIADTSITDFVAYLLQVD-AGLLVKSLVERTIETTHGMRRGSIYNVPLNIVQATAVRDALA
ErcMyo1       278  KAMGVIGLSQAEQDEIFRMLAAILWIGNITFAENDEGNAQVGDTTVTDFLAYLLQVD-PDLLLKSLVERVIETNHGMRRGSIYNVPLNIVQATAVRDALA
HsvMyo1       277  KAMNTIGVTQQEQDEIFRMMAAILWIGNVTFVENDEGNAQVKDTSVTDFVAYLLQVD-SQILIKCLVERIMETNHGMRRGSVYHVPLNRVQATAVRDALA
KlMyo1        276  KAMQVIGLSQEEQDQIFRMLSAILWIGNVTFVENNEGNAEVRDTSVTDFVAYLMQVD-SGILIKCLVERVMETGHGSRRGSVYHVPLNVVQATAVRDALA
KmmMyo1       276  KAMDVIGLSQDEQDQIFRMLSAILWIGNISFIENDEGNSQVRDTSVTDFVAYLLQVD-ASLLIKCLVERVMETGHGARRGSVYHVPLNVVQANAVRDALA
KlwMyo1       276  KAMEIIGLSQDEQDQIFRMLSAILWIGNISFVENDEGNSEVRDTSVTDFVAYLMQVD-SSLLVKCLVERVMETGHGSRRGSVYHVPLNVVQATAVKDALA
KaMyo1        277  KAMQVIGLSQDEQDQIFRMLAAILWIGNISFVENEEGNAQIRDTSVTDFVAYLLQVD-SNLLIKSLVERVMETGHGSKRGSIYHVPLNIVQASAVKDALA
KnMyo1        278  KAMQIIGLSQEEQDQIFRVLSSILWIGNITFAENEEGSAAVRDTSVTDFVAYLLQID-AALLTQSLVERTMETNHGMRRGSIYHVPLNVVQATAVRDALA
LwMyo1        278  NAMSVIGITQHEQDEVFRFLAAILWIGNISFTENEEGNAQVRDTSVTDFVAYLLQVD-SNLLVKSLVERVMETNHGMRRGSIYHVPLNVVQATAVKDALA
LatMyo1       278  KAMQVIGITQQEQDELFRLLSAILWIGNISFTENEEGNAQVCDTSVTDFVAYLLQVD-AHFLTQALVERVMETNHGMRRGSVYHVPLNVVQATAVKDALA
Lak_aMyo1     282  KAMQVIGLSQEEQDQIFRLLATILWIGNISFTEDEEGNAQVRDTSVTDFVAYLLQVD-SQLLIKCLVERVMETSHGMRRGSVYHVPLNIVQATAVKDALA
Sab_aMyo1A    278  EAMRTIGLVQEEQDQIFRMLAAILWIGNISFIENEEGNAQVRDTSVTDFVAYLLQVD-APLLIKCLVERIMQTSHGMKRGSVYHVPLNAVQATAARDALA
Sc_cMyo1A     277  EAMRTIGLVQEEQDQIFRMLAAILWIGNISFIENEEGNAQVGDTSVTDFVAYLLQVD-ASLLVKCLVERIMQTSHGMKRGSVYHVPLNPVQATAVRDALA
SaaMyo1A      278  EAMKTIGLVQEEQDQIFRMLAAILWIGNISFVENEEGNAQVRDTSVTDFVAYLLQVD-ASLLIKCLVERIMQTSHGMRRGSVYHVPLNAVQATAVKDALA
SakMyo1A      268  ------GLGQEDQYQFFRMLAAIMWMANISFIENEEANAQVRDTSVTDFAAYILQVD-APLLIKCMVDRIMQTILGMKRGSVYHVPLNTVQATAVRDALA
SmiMyo1A      278  EAMRTIGLAQEEQDQIFRMLAALLWVGNISFIENEEGNAQVRDTSVTDFVAYLLQVD-ASLLIKCLVERIMQTSHGMRRGSVYHVPLNPVQATAVRDALA
Sap_aMyo1A    278  EAMRTIGLAQEEQDQIFRMLAALLWIGNISFIENEEGNAQIRDTSVTDFVAYLLQVD-ASLLMKCLVERIMQTSHGMRRGSVYHVPLNPVQATAVRDALA
ZrMyo1        278  AAMRVIGLSQEEQDEIFRVLAAILWTGNITFMENDEGNAQVRDTSVTDFVAYLLQVD-SQLLVKSLVERIMETNHGMRRGSVYHVPLTTVHATAVKDALA
TodMyo1       278  KAMQVIGLSQEEQDQIFRLLAAILWIGNITFVEDEEGNAKIRDTSVTDFVAYLLQVE-SELLIKSLVERVLETNHGMRRGSVYHVPLNAVQATAVKDALA
CglMyo1B      278  NAMRTIGLTKSEQDQIFRALAAILWIGNISFVENEAGNAEIRDKSVTTFVAYLLEVQ-EELLIKALIERIIETTHGAKRGSTYHSPLNIIQATAVRDALA
WaMyo1Alpha   278  NAMQTVGITQPEQDQIFRVLAAILWIGNITFVENEEGNAQVRDTSVTDFVAYLLQVD-SETLNKAVTERVMETSHGMRRGSVYNVPLNITQATAVRDALA
WicMyo1       278  SAMQTIGVTQDEQDQIFRILAAILWIGNITFMENDEGNSQVKDTSVTDFVAYLLQVD-SEILIKSITQRVMETSHGMKRGSVYEVPLNITQAIAVKDALA
CyjMyo1       276  KAMQIIGIDQEQQDQIFRILAAILWIGNITFVEDEQGNSTVRDSSVTKFVAYLISVD-EQTLINSLITRVMTTMN-----ETYDIPLNPTQATAVKNALA
YlMyo1        279  AAMNLIGLTQAEQDNLFKLLAAILWIGNMSFVEDKDGNAAIADVSVPNFVAYLLEVD-AESVVKAVTQRIMETSRGGRRGSVYEVALNIAQATSVRDALA
CdnMyo1B      278  NAMNIIGLSKEEQDQIFRMLAAILWIGNISFIEDEQGNAQIRDTSVTTFVAYLLQVQ-EELLIKSLVERIIETGHGAKRGSTYHSPLNIVQATAVRDALA
Kop_bMyo1     279  KAMQTIGLTQDEQDQVFRILAAILWIGNISFVENEDGNAQIRDSSVTDFVAYLLQVN-AQILTNSLIERIVETSHGSKRGSIYHVPLNITQATAVRDALS
NdMyo1B       277  RAMDIIGLAKEEQDQIFRLLAAILWIGNISFVENEQGNAQIRDTSVTTFVAYLLQVQ-EDMLIKSLVERVIETSHGAKRGSTYHSPLNIVQATAVRDALA
KcMyo1        280  KAMRTIGMTQEEQDELFSLLAAVLWIGNVSFAEDAEGNSTIRDTSVTDFVAYLLQVD-SQILCKSLTERTLETNHGMKRGSIYHVPLNMTQATAVRDALA
OgpMyo1       284  ASMTTIGLTQEEQDQVFRVLAAILWIGNITFVEDAEGNAQVRDTGVTDFVAYLLQVN-SEVLVKSIIERTMETSHGMRRGSVYHVPLNPVQATASRDALA
DebMyo1       283  KAMDVIGITDEERDQIFRILAGILWIGNITFTEDEEGNAAIADTSVTDFVAYLLQVD-AQTLCKSVVERTIQTFHGMRRGSIYHSPLNPVQATASRDALA
PiuMyo1       284  KAMDTIGLSSEERDHVFRLLSAILWIGNVSFIEDEEGNSKIRDESVTNFVAYLLQVD-ATLLCKSLTERTMETSHGMKRGSIYHVPLNTTQATAVKDALA
NabMyo1B      278  RAMETIGISQEEQDNIFRLLAAILWTGNISFVENEEGNAQIRDTSVTDFVAYLLQVD-SQMLIGSIIERIMETSHGSRRGSIYNVPLNIVQATAVKDALA
ZbMyo1        278  KAMQVIGLTQEEQDEIFRVLAAILWIGNITFGENDEGNAEVRDTSVTDFVAYLLQVD-SQLLIKSLVERIMETNHGMRRGSVYHVPLNTVQATAVRDALA
Ca_bMyo1      287  NAMKIIGLTQQEQDNIFRMLASILWIGNISFVEDENGNAAIRDDSVTNFAAYLLDVN-PEILKKAIIEKTIETSHGMRRGSTYHSPLNIVQATAVRDALA
StaMyo1       285  NAMNIIGLTQDEQDNIFKMLAAILWIGNISFVEDESGNAAIRDETVTQFVAYLLDVPSAEIMKKAIIERTIETTHGSRRGSTYHVPLNIVQATSVRDALA
ShpMyo1       284  NAMNIIGLTQEEQDNIFRMLAAILWVGNISFVEDESGNAAIRDETVTQFVAYLLDVS-PEIMKKAIIERTIETTHGSRRGSTYHVPLNIVQATSVRDALA
CllMyo1       286  RAMEIIGLSQAEQDNIFRMLASILWIGNISFVEDESGNATIRDAGVTNFVAYLLEVS-PEILQKAIVERIIETSHGMRRGSTYHVPLNIVQATAVRDALA
MefMyo1       285  NAMNVIGLAQVEQDNIFRMLAAILWIGNISFVEDENGNAAVRDEGVTNFVAYLLEVN-AEILKKSIIERIIETSHGMRRGSTYHVPLNITQATAVRDALA
CnmMyo1       285  NAMQVIGLTQQEQDSIFRMLASILWIGNISFVEDEHGNAAIRDESVTNFVGYLLDVS-GEIVKKSIIERTIETSFGSRRGSTYHSPLNIVQATAVRDALA
DhhMyo1       285  RAMQVIGLSQEEQDNIFRMLASILWIGNISFVEDEHGNAAIRDESVTAFVAYLLDVN-AETLKTSLIQRVMQTSHGMRRGSTYHVPLNIVQATSVRDALA
DehMyo1       285  RAMQVIGLSQDEQDNIFRMLASILWVGNVSFVEDDNGNAAVRDESVTAFIAYLLDVD-AETLKTSLIQRVMQTSHGMRRGSTYHVPLNIVQATSVRDALA
CatMyo1       285  NAMKVIGITPQEQDHIFRMLAAILWVGNISYVEDEHGNAAIRDESVINFVAYLLETD-AESVKKSITEKIVQTSHGMRRGSTYHSPLNIVQATAVRDALA
LoeMyo1       292  NAMNIIGLSQAEQDNIFRILASILWTGNISFVEDESGNAAIRDDSVTNFVAYLLDVN-AEILKKAITERTIETSHGMRRGSTYHVPLNIVQATAVRDALA
MrgMyo1       283  QAMNTIGLSKAEQDNIFRSLASILWIGNISFVENEDGNAAIRDDTVTTFVAYLLEVD-ANVLKKSILERVIETSHGMRRGSTYHVPLNIVQATASRDALA
ShsMyo1       281  SAMKIIGLTELEQNNIFRMIASILWIGNVSFVEDESGNAAIRDDSVTQFVAYLLEVN-PEILKKAIVERVIETTHGMRRGSTYHVPLNIVQATSVRDALA
CameMyo1      286  NAMNVIGLTQDEQDNIFRMLASILWIGNISFVEDESGNAAIRDDSVTNFVAYLLDVN-PEILKKAIIERTIETSHGMKRGSTYHVPLNIVQATAVRDALA
MifMyo1Alpha  284  KAMQIIGLTQDEQDNIFRVLASILWIGNISFVEDENGNAIPRDESVTNFVAYLLNVE-PVILKTAFVQRVMQTSHGMRRGSTYHVPLNIVQATAVRDALA
CadMyo1       288  NAMKIIGLTQQEQDNIFRMLAVILWIGNISFIEDENGNATIRDDSVTNFVAYLLDVN-SEILKKAIIERTIETSHGMRRGSTYHSPLNIVQATAVRDALA
CaoMyo1       286  NAMNVIGLSQEEQDNIFRMLASILWIGNISFVEDESGNAAIRDDSVTNFVAYLLDVN-PEILKKAIIERTIETSHGMKRGSTYHVPLNIVQATAVRDALA
CapMyo1       286  NAMNVIGLSQDEQDNIFRMLASILWIGNISFIEDESGNAAIRDDSVTNFVAYLLDAN-PDILKKAIIERTIETSHGMRRGSTYHVPLNIVQATAVRDALA
Ct_aMyo1      284  NAMQIIGLSQDEQDQIFRMLASILWVGNISFVEDENGNAAIRDESVTNFVAYLLGVN-TEILKKAIIERTIETSHGMRRGSTYHSPLNIVQATAVRDALA
PtaMyo1       282  NAMSIIGLSQEEQDQIFRILSAILWIGNVLFVENEDANSAIGDQGVINYIAYLLQVD-AEQLAKSLTERIMETSHGMKRGSIYHVPLNLTQAYAVRDALA
CacaMyo1B     281  NAMKTIGLSQTEQDHIFRLLAAILWIGNISFIENEEGSAQIRDTSVTDFVAYLLEVD-SSLLITSIVERILETSHGTKRGSIYHTPLNIVQATAVRDGLA
NadMyo1       278  KAMQTIGLAQEEQDQIFRMLAAILWIGNISFIENEEGNAQVRDTSVTDFVAYLLQVD-AAVLIKALVERIMETSHGMRRGSVYHVPLNIVQATAVRDALA
```

```
                              410       420       430       440       450       460       470       480       490       500
                     ....|....|....|....|....|....|....|....|....|....|....|....|....|....|....|....|....|....|....|....|
CnbMyo1A        377  KAIYNNLFDWIVDRVNISLQAFPGAD---KSIGILDIYGFEIFEHNSFEQICINYVNEKLQQIFIQLTLKSEQETYEKEQIQWTPIKYFDNKVVCDLIEA
CdnMyo1A        377  KAIYNNLFEWIVDRVNISLQAFPGAD---KSIGILDIYGFEIFEHNSFEQICINYVNEKLQQIFIQLTLKSEQETYEREQIQWTPIKYFDNKVVCDLIEA
CacaMyo1A       378  KAIYNNLFDWIVDRVNISLQAFSGAE---KSIGILDIYGFEIFEHNSFEQICINYVNEKLQQIFIQLTLKSEQETYEREKIEWTPIKFFDNKVVCELIEA
NabMyo1A        377  KAIYNNLFDWIVDRVNISLQAYPGAD---KSIGILDIYGFEIFEHNSFEQICINYVNEKLQQIFIQLTLKSEQETYEREKIQWTPIKFFDNKVVCDLIEA
NdMyo1A         377  KAIYNNLFEWIVDRVNISLQAFPGAD---KSIGILDIYGFEIFEHNSFEQICINYVNEKLQQIFIQLTLKSEQETYEREQIQWTPIKYFDNKVVCDLIEA
CnbMyo1B        377  KGIYNNLFDWIVERVNVSLQAFPGAD---KSIGILDIYGFEIFEHNSFEQICINYVNEKLQQIFIQLTLKSEQETYQKEQIQWTPIKYFDNKVVCDLIEA
CglMyo1A        378  KAIYNNLFEWIVDRVNKSLHAYPGAD---KSIGILDIYGFEIFEHNSFEQICINYVNEKLQQIFIQLTLKSEQDTYAREKIQWTPIKYFDNKVVCDLIEA
Sab_aMyo1B      377  KAIYNNLFDWIVGRVNKSLQAFPGAE---KSIGILDIYGFEIFEHNSFEQICINYVNEKLQQIFIQLTLKSEQETYEREKIQWTPIKYFDNKVVCDLIEA
Sc_cMyo1B       377  KAIYNNLFDWIVSRVNKSLQAFPGAE---KSIGILDIYGFEIFEHNSFEQICINYVNEKLQQIFIQLTLKSEQETYEREKIQWTPIKYFDNKVVCDLIEA
SaaMyo1B        377  KAVYNNLFDWIVGRVNKSLQAFPGAE---KSIGILDIYGFEIFEHNSFEQICINYVNEKLQQIFIQLTLKSEQETYEREKIQWTPIKYFDNKVVCDLIEA
SakMyo1B        377  KAIYNNLFDWIVGRVNKSLQAFPGAE---KSIGILDIYGFEIFEHNSFEQICINYVNEKLQQIFIQLTLKSEQETYERERIEWTPIKYFDNKVVCDLIEA
SmiMyo1B        377  KAIYNNLFDWIVGRVNKSLQAFSGAE---KSIGILDIYGFEIFEHNSFEQICINYVNEKLQQIFIQLTLKSEQETYEREKIQWTPIKYFDNKVVCDLIEA
Sap_aMyo1B      377  KAIYNNLFDWIVSRVNKSLQAFPGAE---KSIGILDIYGFEIFEHNSFEQICINYVNEKLQQIFIQLTLKSEQETYEREKIQWTPIKYFDNKVVCDLIEA
NacMyo1         377  KAIYNNLFEWIVGRVNVSLQAYPGAD---RSIGILDIYGFEIFEHNSFEQICINYVNEKLQQIFIQLTLKSEQETYKREKIKWTPIKYFDNKVVCDLIEA
KaaMyo1         377  KAIYNNLFDWIVDRVNVSLQAFPGAD---KSIGILDIYGFEIFEHNSFEQICINYVNEKLQQIFIQLTLKSEQEEYAREKIQWTPIKYFDNKVVCDLIEA
VpMyo1          377  KAIYNNMFDWIVERVNISLQAYPGAD---KSIGILDIYGFEIFEHNSFEQICINYVNEKLQQIFIQLTLKSEQETYEREQIQWTPIKFFDNKIVCDLIES
TtpMyo1A        377  KAIYNNLFDWIVERVNVSLQAYPGAD---KSIGILDIYGFEIFEHNSFEQICINYVNEKLQQIFIQLTLKSEQETYEKEQIQWTPIKFFDNKVVCELIEG
TtpMyo1B        377  KAIYNNLFDWIVERVNVSLQAYPGAD---KSIGILDIYGFEIFEHNSFEQICINYVNEKLQQIFIQLTLKSEQETYEKEQIQWTPIKFFDNKVVCELIEG
TtbMyo1A        381  KAIYSNLFDWIVDRVNLSLQGNDGAANADKAIGILDIYGFEIFEHNSFEQLCINYVNEKLQQIFIQLTLKSEQETYEREQIQWTPIEFFDNKVVCDLIEG
TtbMyo1B        379  KAIYNNLFDWIVDRVNLSLQAYPGAD---KSIGILDIYGFEIFEHNSFEQLCINYVNEKLQQIFIQLTLKSEQETYAREQIAWTPIKYFDNKVVCDLIES
ErgMyo1         376  KAIYNNLFEWIVDRVNVSLQALPGAE---KSIGILDIYGFEIFEHNSFEQICINYVNEKLQQIFIQLTLKSEQEEYAKEQIQWTPIKYFDNKVVCELIEA
ErcMyo1         377  MAIYNNLFEWIVDRVNVSLHAFPGAD---KSIGILDIYGFEIFDHNSFEQICINYVNEKLQQIFIQLTLKSEQDEYAREQIQWTPIKYFDNKVVCDLIEA
HsvMyo1         376  KAIYSNLFDWIVSRVNVSLGALSGGD---KSIGILDIYGFEIFEKNSFEQLCINYVNEKLQQIFIQLTLKSEQEEYAREKIQWTPIQYFDNKVVCDLIEA
KlMyo1          375  KAIYNNLFDWIVDRVNVSLKAFPGAV---KSIGILDIYGFEIFEHNSFEQICINYVNEKLQQIFIQLTLKSEQEEYNKEQIQWTPIKYFDNKVVCDLIES
KmmMyo1         375  KAIYSNLFDWIVDRVNVSLKAFPGAV---KSIGILDIYGFEIFEHNSFEQICINYVNEKLQQIFIQLTLKSEQEEYNREQIQWTPIKYFDNKVVCDLIEA
KlwMyo1         375  KAIYNNLFEWIVDRVNVSLKAFPGAV---KSIGILDIYGFEIFEHNSFEQICINYVNEKLQQIFIQLTLKSEQEEYEKEQIQWTPIKYFDNKVVCDLIEA
KaMyo1          376  KGIYNNLFEWIVDRVNISLQAYPGAV---KSIGILDIYGFEIFEHNSFEQICINYVNEKLQQIFIQLTLKSEQEEYKREQIEWTPIKYFDNKVVCDLIES
KnMyo1          377  KAIYNNLFDWIVDRVNVSLQAYPGAD---KSIGILDIYGFEIFEHNSFEQICINYVNEKLQQIFIQLTLKQEQETYEKEQIEWTPIKYFDNKVVCDLIEA
LwMyo1          377  KAIYNNLFDWTVDRVNLSLQALPGAN---KSIGILDIYGFEIFDYNSFEQICINYVNEKLQQIFIQLTLKSEQEEYAREQIEWTPIKYFDNRVVCDLIEA
LatMyo1         377  KAIYNNLFDWIVDRVNVSLQAFPGAN---KSIGILDIYGFEIFDYNSFEQICINYVNEKLQQIFIQLTLKSEQEEYAREQIQWTPIKYFDNRVVCDLIEA
Lak_aMyo1       381  KAIYNNLFEWIVDRVNVSLQAYPGAD---KSIGILDIYGFEIFDHNSFEQICINYVNEKLQQIFIQLTLKSEQEEYAREQIQWTPIKYFDNKVVCELIEA
Sab_aMyo1A      377  KAIYNNLFNWIVDRVNVSLQAFPGAD---KSIGILDIYGFEIFEHNSFEQICINYVNEKLQQIFIQLTLKAEQETYEREKIKWTPIKYFDNKVVCELIEA
Sc_cMyo1A       376  KAIYNNLFDWIVDRVNVSLQAFPGAD---KSIGILDIYGFEIFEHNSFEQICINYVNEKLQQIFIQLTLKAEQETYEREKIKWTPIKYFDNKVVCDLIEA
SaaMyo1A        377  KAIYNNLFDWIVDRVNVSLQAFPGAD---KSIGILDIYGFEIFEHNSFEQICINYVNEKLQQIFIQLTLKAEQETYEREKIKWTPIKYFDNKVVCDLIEA
SakMyo1A        362  KGIYDNLFNWIVDRVNVSLQAFPGAD---KSIGILDIYGFEIFEQNSFEQICINYVNEKLQQIFIQLTLKAEQETYEREKIKWMPIKYFDNKVVCDLIEA
SmiMyo1A        377  KAIYNNLFDWIVERVNVSLQAFPGAD---KSIGILDIYGFEIFEHNSFEQICINYVNEKLQQIFIQLTLKAEQETYEKEKIKWTPIKYFDNKVVCDLIEA
Sap_aMyo1A      377  KAIYNNLFDWIVDRVNVSLQAFPGAD---KSIGILDIYGFEIFEHNSFEQICINYVNEKLQQIFIQLTLKAEQETYEREKIKWTPIKYFDNKVVCDLIEA
ZrMyo1          377  KALYNNLFDWIVDRVNQSLQAYPGAE---KSIGILDIYGFEIFEHNSFEQICINYVNEKLQQIFIQLTLKQEQEEYAREKIQWTPIKYFDNKVVCDLIEG
TodMyo1         377  KGIYNNLFDWIVDRVNISLQAFPGAD---KSIGILDIYGFEIFEHNSFEQICINYVNEKLQQIFIQLTLKSEQETYDRERIQWTPIKYFDNKVVCDLIEA
CglMyo1B        377  KAIYNNLFEWIVERVNNSLQAFPGAD---KSIGILDIYGFEIFEHNSFEQICINYVNEKLQQIFIQLTLKSEQDTYKKEQIHWTPIEYFDNKIVCDLIEA
WaMyo1Alpha     377  KGLYNNLFDWIVARVNKALQTVGGAS---KSIGILDIYGFEIFEHNSFEQICINYVNEKLQQIFIQLTLKSEQDEYVKEQIQWTPINYFNNKVVCDLIEL
WicMyo1         377  KGLYNNLFDWIVARVNKALQTIGGAS---KSIGILDIYGFEIFEHNSFEQICINYVNEKLQQIFIQLTLKSEQDEYVREQIQWTPINYFNNKIVCDLIEA
CyjMyo1         370  AAMYSVLFDHIIVILNKVLDKGNKY----KSIGILDIYGFEIFEHNSFEQLCINYVNEKLQQIFIQLTLKSEQDEYVREQIQWTPIQFFNNKIVVDLIEA
YlMyo1          378  KGIYNNLFDWIVERVNQSLRAPQDAA---RTIGILDIYGFEIFESNSFEQICINYVNEKLQQIFIQLTLKTEQDEYVREQIAWTPINYFNNKIVCDLIEE
CdnMyo1B        377  KAIYNNLFEWIVDRVNISLQAFPGAD---KSIGILDIYGFEIFEHNSFEQICINYVNEKLQQIFIQLTLKSEQDTYQKEQIQWTPIKYFDNKVVCDLIEA
Kop_bMyo1       378  KGIYSNLFDWIVDRTNKSLAHISETVH--KTIGILDIYGFEIFEQNSFEQLCINYVNEKLQQIFIQLTLKSEQDEYVKEQIQWTPIKYFNNKIVCDLIEA
NdMyo1B         376  KAIYNNLFEWIVDRVNTSLQAFPGAD---KSIGILDIYGFEIFEHNSFEQICINYVNEKLQQIFIQLTLKTEQDTYQKEQIQWTPIKYFDNKIVCDLIEA
KcMyo1          379  KGIYNNLFDWIVERVNASLKGLQQHVN--KSIGILDIYGFEIFEHNSFEQLCINYVNEKLQQIFIELTLKSEQDEYVREQIQWTPIKYFNNKIVCDLIEA
OgpMyo1         383  KGLYNNMFDWIVERVNKSFQSLNLEAH--RSIGILDIYGFEIFEHNSFEQLCINYVNEKLQQIFIQLTLKSEQDEYVREQIQWTPINYFNNKIVCDLIES
DebMyo1         382  KGLYTNLFDWIVDRVNLSFSNMNSATF--KSIGILDIYGFEIFEHNSFEQLCINYVNEKLQQIFIQLTLKSEQDEYVREKIAWTPIKYFNNKVVCDLIEA
PiuMyo1         383  MGIYNNLFDWIVDRVNQSFSSNKSDIF--KSIGILDIYGFEIFEHNSFEQLCINYVNEKLQQIFIQLTLKSEQDEYVKEQIQWTPIKFFNNQIVCDLIES
NabMyo1B        377  KALYSHLFDWIVDRVNSSLQAFPGAE---KSIGILDIYGFEIFDHNSFEQLCINYVNEKLQQIFIQLTLKSEQETYEREKIQWTPIEFFDNKVVCDLIEA
ZbMyo1          377  KALYNNLFDWIVDRVNQSLQAYPGAE---KSIGILDIYGFEIFEHNSFEQICINYVNEKLQQIFIQLTLKQEQEEYAREQIKWTPIKYFDNKVVCDLIEG
Ca_bMyo1        386  KGIYNNLFEWIVERVNISLAGSQQQSS--KSIGILDIYGFEIFEHNSFEQICINYVNEKLQQIFIQLTLKAEQDEYVQEQIKWTPIDYFNNKVVCDLIEA
StaMyo1         385  KGIYNNLFEWIVQRVNVSLKGVQQSD---KTIGILDIYGFEIFDHNSFEQVCINYVNEKLQQIFIQLTLKAEQEEYVQEQIKWTPIDYFNNKVVCDLIEA
ShpMyo1         383  KGIYNNLFEWIVERVNVSLKGVQQSD---KTIGILDIYGFEIFEHNSFEQVCINYVNEKLQQIFIQLTLKAEQDEYVQEQIKWTPIEYFNNKVVCDLIEE
CllMyo1         385  KGIYNNLFEWIVERVNASLSAAGQNLEK-KSVGILDIYGFEIFEHNSFEQICINYVNEKLQQIFIQLTLKAEQDEYVQEQIKWTPIDYFNNKVVCDLIEA
MefMyo1         384  KGIYNNLFEWIVERVNLSLKDSGQNYAK-KSIGILDIYGFEIFEQNSFEQICINYVNEKLQQIFIQLTLKAEQDEYVQEQIQWTPIDYFNNKVVCDLIEA
CnmMyo1         384  KGIYNNLFEWIVERVNISLQGHQQQSS--KSIGILDIYGFEIFDHNSFEQVCINYVNEKLQQIFIQLTLKAEQDEYVQEQIKWTPIEYFNNKVVCDLIEA
DhhMyo1         384  KGIYNNLFDWIVERVNLSLKGSGPAYEK-KSIGILDIYGFEIFEHNSFEQICINYVNEKLQQIFIQLTLKAEQDEYVQEQIKWTPINYFNNKVVCDLIEA
DehMyo1         384  KGIYNNLFDWIVERVNLSLQGSGAVQEK-KSIGILDIYGFEIFEHNSFEQICINYVNEKLQQTFIQLTLKAEQDEYVQEQIKWTPIDYFNNKVVCDLIEA
CatMyo1         384  KGLYNNLFDWIVARVNVSLKGEGPGMEK-KSIGILDIYGFEIFEHNSFEQICINYVNEKLQQIFIQLTLKAEQDEYVQEQIKWTPIEYFNNKVVCDLIEA
LoeMyo1         391  KGLYNNLFDWIVERVNLSLQGNQGQSE--KSIGILDIYGFEIFEHNSFEQICINYVNEKLQQIFIQLTLKAEQDEYVQEQIKWTPIDYFNNKVVCDLIEA
MrgMyo1         382  KGIYNYLFDWIVERVNISLRGRAEAMEK-KTIGILDIYGFEIFEHNSFEQICINYVNEKLQQIFIQLTLKAEQDEYVQEQIKWTPIDYFNNKVVCDLIEA
ShsMyo1         380  KGIYNNLFEWIVERVNVSLKGASQSS---KTIGILDIYGFEIFEHNSFEQICINYVNEKLQQIFIQLTLKAEQDEYVKEQIKWTPIDYFNNKVVCDLIEA
CameMyo1        385  KGIYNNLFDWIVERVNLSLGGKQHQSN--KSVGILDIYGFEIFEHNSFEQVCINYVNEKLQQIFIQLTLKAEQEEYVQEQIKWTPIDYFNNKVVCDLIEA
MifMyo1Alpha    383  KGLYNNLFDWIVERVNLSLKNTDVSSER-KSIGILDIYGFEIFEHNSFEQICINYVNEKLQQIFIQLTLKAEQDEYVQEQIKWTPINYFNNKVVCDLIEA
CadMyo1         387  KGIYNNLFEWIVERVNVSLSSHQQQSC--KTIGILDIYGFEIFEHNSFEQICINYVNEKLQQIFIQLTLKAEQDEYVQEQIKWTPIDYFNNKVVCDLIEA
CaoMyo1         385  KGIYNNLFDWIVERVNLSLGGKENQSN--KSIGILDIYGFEIFEHNSFEQVCINYVNEKLQQIFIQLTLKAEQDEYVQEQIKWTPIDYFNNKVVCDLIEA
CapMyo1         385  KGIYNNLFDWIVERVNLSLGGKQNQSN--KSIGILDIYGFEIFEHNSFEQVCINYVNEKLQQIFIQLTLKAEQDEYVQEQIKWTPIDYFNNKVVCDLIEA
Ct_aMyo1        383  KGIYNNLFEWIVERVNKSLQGHQQQSA--RSIGILDIYGFEIFEHNSFEQICINYVNEKLQQIFIQLTLKAEQDEYVQEQIKWTPIDYFNNKVVCDLIEA
PtaMyo1         381  KGLYNNLFDWIVKRVNISLNTSGSSGSAAKSIGILDIYGFEIFDNNSFEQFCINYVNEKLQQVFIQLTIKEEQEEYKREQITWTPIKYFDNKVVCELIED
CacaMyo1B       380  KAIYNNLFDWIVDRVNLSLEAFSGAE---KSIGILDIYGFEIFEQNSFEQLCINYVNEKLQQIFIQLTLKSEQETYEREKIQWTPIKFFDNKVVCDLIEA
NadMyo1         377  KAIYSNLFDWIVGRVNKSLQAYSGAD---KSIGILDIYGFEIFEHNSFEQLCINYVNEKLQQIFIQLTLKSEQETYEREKIQWTPIKYFDNKVVCDLIEA
```

```
                         510       520       530       540       550       560       570       580       590       600
                 ....|....|....|....|....|....|....|....|....|....|....|....|....|....|....|....|....|....|....|....|
CnbMyo1A     474 R-RPPGIFAAMNDSVATAHADSSAADQAFAQRLNLFSS-NPHFELRQNKFVIKHYAGDVTYDVLGITDKNKDQLQKDLVELVGTTTNPFLTT-LFPNQVD
CdnMyo1A     474 R-RPPGIFAAMNDSVATAHADSSAADQAFAQRLNLFSS-NPHFEQRQNKFVIKHYAGDVTYDVLGITDKNKDQLQKDLVELVGTTTNPFLTT-LFPNQVD
CacaMyo1A    475 R-RPPGIFAAMNDSVATAHADSNAADQAFAQRLNLFSS-NPHFELRQNKFVIKHYAGDVTYDINGITDKNKDQLQKDLVELINTSSNTFLTA-LFPEEID
NabMyo1A     474 R-RPPGIFAAMNDSVATAHADSNAADQAFSQRLNLFTS-NPHFELRQNKFVIKHYAGDVTYDVNGITDKNKDQLQKDLVELVGTTSNPFLST-IFPVQID
NdMyo1A      474 R-RPPGIFAAMNDSVATAHADSSAADQAFAQRLNLFSS-NPHFELRQNKFVIKHYAGDVTYDVLGITDKNKDQLQKDLVELVGTTTNPFLMN-LFPNNVD
CnbMyo1B     474 R-RPPGIFAAMNDAIATAHADSNAADQAFSQRLNLFST-NPHFELRQNKFVIKHYAGDVTYDVLGITDKNKDQLQKDLVELLSTTSNDFVQT-IFPNQVD
CglMyo1A     475 K-RPPGIFAAMNDSVATAHADSSAADQAFAQRLSLFSS-NPHFEQRQNKFVIKHYAGDVTYDVLGMTDKNKDQLQKDLVELVGTTTNAFLTT-LFPNQVD
Sab_aMyo1B   474 R-RPPGIFAAMNDSVATAHADSNAADQAFAQRLNLFTT-NPHFELRSSKFVIKHYAGDVTYDIDGITDKNKDQLQKDLVELLGTTTNTFVAT-IFPDNVD
Sc_cMyo1B    474 R-RPPGIFAAMNDSVATAHADSNAADQAFAQRLNLFTT-NPHFDLRSNKFVIKHYAGDVTYDIDGITDKNKDQLQKDLVELIGTTTNTFLAT-IFPDTVD
SaaMyo1B     474 R-RPPGIFAAMNDSVATAHADSDAADQAFAQRLNLFTT-NPHFALRSNKFVIKHYAGDVTYDIDGITDKNKDQLQKDLVELIGTTTNTFLAT-IFPDTVD
SakMyo1B     474 R-RPPGIFAAMNDSVATAHADSNAADQAFAQRLNLFTT-NPHFDLRSNKFVIKHYAGDVTYDIDGITDKNKDQLQKDLVELIGTTSNTFLAA-IFPDTID
SmiMyo1B     474 R-RPPGIFAAMNDSVATAHADSNAADQAFAQRLNLFTT-NPHFDLRSNKFVIKHYAGDVTYDIDGITDKNKDQLQKDLVELIGTTTNAFLAT-IFPDTVD
Sap_aMyo1B   474 R-RPPGIFAAMNDSVATAHADSNAADQAFAQRLNLFTT-NPHFDLRSNKFVIKHYAGDVTYDIDGITDKNKDQLQKDLVELIGTTTNTFLAT-IFPDTVD
NacMyo1      474 K-RPPGIFAAMNDSVATAHADSNAADQAFAQRLNMFSS-NPHFELRSNKFVIKHYAGDVTYDVNGITDKNKDQLQKDLVELIATTTNPFLSG-IFPVEVD
KaaMyo1      474 R-RPPGIFAAMNDSVATAHADSSAADQAFAQRLNMFST-NPHFEPRQGKFVIKHYAGDVTYDINGITDKNKDQLQKDLVELLSTTQNTFVNT-IFPAEVD
VpMyo1       474 R-RPPGIFAAMNDSVATAHADSDAADQAFSQRLNLFST-NPHFALRSNKFVIKHYAGDVTYDVNGITDKNKDQLQRDLVELIATSSNAFLRT-IFPDNVD
TtpMyo1A     474 R-RPPGIFAAMNDSVATAHADSNAADQAFSQRLNLFSS-NPHFELRSNKFVIKHYAGDVTYDVNGITDKNKDQLQKDLAELIGTTQNGFLKS-LFPEQVN
TtpMyo1B     474 R-RPPGIFAAMNDSVATAHADSNAADQAFSQRLNLFSS-NPHFELRSNKFVIKHYAGDVTYDVNGITDKNKDQLQKDLAELIGTTQNGFLKS-LFPEQVN
TtbMyo1A     481 R-RPAGIFAAMNDSIATAHADSDAADQAFSQSLDMFRS-NPNFDMRSSKFVIKHYAGDVTYDIDGITDKNKDTLQKDLVELLGTTQNEFVTT-IFPQQVN
TtbMyo1B     476 K-RPAGIFAAMNDSVATAHADSNAADSAFSQRLNLFTT-NPHFELRSSKFVIKHYAGDVMYDVNGITDKNKDQLQKDLSELLGTSTNQFVST-IFPNEVD
ErgMyo1      473 K-RPPGIFAALNDSVATAHADSNAADQAFAQRLNLFTS-NPHFELRSSKFVIKHYAGDVTYDIGGMTDKNKDQLQRDLVELLNSTSNTFLAT-IFPDTGD
ErcMyo1      474 R-RPPGIFAALNDSVATAHADSNAADQAFAQRLNFFSS-NPHFEARSSKFIIKHYAGDVTYDIDGMTNKNKDHLQKDLVDLIGTTSNPFLST-IFTDGSD
HsvMyo1      473 R-RPPGIFAAMNDAVATAHADSSAADQTFAQRLNLFTS-NKHFDLRQNKFVIKHYAGDVIYDVLGISDKNKDQLQKDLVELISTTSNAFLKT-IFPDTVN
KlMyo1       472 K-RPPGIFATLDDSVATAHADSNAADQAFAQRLNLFST-NAHFDLRSSKFVIKHYAGDVTYDISGMTDKNKDQLVKDLAELVQTTTNPFLST-IFPDTID
KmmMyo1      472 K-RPPGIFATLDDSVATAHADSSAADQAFAQRLNMFST-NPHFDLRSSKFVIKHYAGDVTYDIMGMTDKNKDQLQKDLAELIQTTTNPFLMN-IFPDAVD
KlwMyo1      472 K-RPPGIFATLDDSVATAHADSSAADQAFAQRLNMFSN-NPHFDLRSTKFVIRHYAGEVTYDINGMTDKNKDQLQKDLAELIETTTNSFLST-IFPDAVD
KaMyo1       473 K-RPPGIFATLNDSVATAHADSNAADQAFAQRLNLFSS-NAHFSLRSSKFVIKHYAGDVTYDILGMTDKNKDQLQKDLVELLGTSSNNFLSV-IFPDTVD
KnMyo1       474 K-RPPGIFAAMNDSVATAHADSNAADQAFAQRLNLFST-NPYFELRQNKFVIKHYAGDVTYDINGITDKNKDQLQKDLIELMGTTQNPFIHT-IFPAEID
LwMyo1       474 R-RPPGIIAAMNDSVATAHADSDAADQAFAQRLNLFSS-NPHFELRSSKFIIKHYAGDVTYDIFGMTDKNKDQLQKDLVELVKTSSNEFLCS-LFPTVVD
LatMyo1      474 R-RPPGIIAAMNDSVATAHADSNAADQAFAQRLNLFSS-NPHFELRSTKFIIKHYAGDVTYDIFGMTDKNKDQLQKDLVELIGTSSNTFLSS-IFPTTVD
Lak_aMyo1    478 K-RPPGIFAALNDSVATAHADSSAADQAFAQRLNLFTS-NAHFELRSSKFIIKHYAGDVTYDILGMTDKNKDLLQKDLVELVGTSSNTFLSA-IFPDTVD
Sab_aMyo1A   474 K-RPPGIFAAMNDSIATAHADSNAADQAFAQRLNLFNS-NPYFELRANKFVVKHYAGDVTYDINGITDKNKDQLQKDLIELVGTTTNTFLST-IFPEDID
Sc_cMyo1A    473 K-NPPGILAAMNDSIATAHADSNAADQAFAQRLNLFNS-NPYFELRANKFVIKHYAGDVTYDINGITDKNKDQLQKDLIELIGTTTNTFLST-IFPDDVD
SaaMyo1A     474 K-RPPGIFAAMNDSIATAHADSNAADQAFAQRLNLFNS-NPYFELRANKFVIKHYAGDVTYDINAITDKNKDQLQKDLIELVGTTANPFLSK-IFPNDID
SakMyo1A     459 K-RPPGIFAAMNDSIATAHADSNAADQAFAQRLNLFNS-NQYFELRANKFVIKHYAGDVTYDINGITDKNKDSLQKDLIELVGTTANTFLST-IFPDDID
SmiMyo1A     474 K-RPPGIFAAMNDSIATAHADSNAADQAFAQRLNLFNS-NPYFELRANKFVIKHYAGDVTYDINGITDKNKDQLQKDLIELIGTSANPFLSS-IFPDDID
Sap_aMyo1A   474 K-RPPGIFAAMNDSIATAHADSNAADQAFAQRLNLFNS-NPYFELRANKFVIKHYAGDVTYDINGITDKNKDQLQKDLIELIGTTTNTFLST-IFPDDVD
ZrMyo1       474 R-RPPGIFAMMNDSVATAHADPSAADQAFAGRLSMFAS-NPHFEMRSSKFIIKHYAGDVTYDVHGITDKNKDQLQKDLVELIGTSANPFINN-IFPTTD
TodMyo1      474 K-RPPGIFAAMNDSVATAHADSNAADQAFAQRLNLFSS-NPHFELRSSKFIIKHYAGDVTYDVNGITDKNKDQLQKDLVELIGTTTNTFLST-IFPDAID
CglMyo1B     474 K-RPPGIFAAMNDAIATAHADSDAADQAFAQRLNLFTT-NPHFELRQNKFVVKHYAGDVTYDIFGITDKNKDQLQKDLVELLSTTSNSFVRE-IFPDQPQ
WaMyo1Alpha  474 K-RPPGIFAALNDSCATAHADSQAADQSFAQRLNMVSA-NKHFELRQSKFVIKHYAGDVTYDINGMTDKNKDQMLKDLLDLATSSNEFLQT-LFPETVN
WicMyo1      474 K-RPPGIFAALNDSCATAHADSSAADQSFAQRLNMVTS-NKHFELRQSKFLVKHYAGDVTYEINGMTDKNKDQMLKDLLDLLSTSSNQFLTN-LFPETID
CyjMyo1      466 K-RPPGVMAALNDACATAHAKSDESDASFAQRLNLMTS-NQHFELRQAKFVIKHYAGDVTYDIQHMTDKNKDQLQKDLMEMLATSQNSFIHT-LFPKPVD
YlMyo1       475 K-RPPGIFAALNDACATAHADPNAADENFIQRMSMVGQ-NPHFEQRQRKFVIKHYAGDVTYDVKGITDKNKDQLLKDLLLQKSGNPFLVS-LFPDPVN
CdnMyo1B     474 K-RPPGIFAAMNDAIATAHADSDAADQAFAQRLNLFSS-NPHFELRQNKFVIKHYAGDVTYDVLGITDKNKDQLQKDLVELLTTTSNTFVQT-IFPTQVD
Kop_bMyo1    476 K-RPPGIFSILDDACATAHADSSAADQAFSQRLNMIAT-NPHFDLRSSKFVIKHYAGDVTYDIDTITDKNKDQMLKDLLEMIKTSQDSFLLS-LFPEPVE
NdMyo1B      473 K-RPPGIFAAMNDAIATAHADSNAADQAFSQRLNLFSS-NPHFELRQSKFVIKHYAGDVTYDIIGITDKNKDQLQKDLVELLTTTSNTFVQA-IFPQHID
KcMyo1       477 K-RPPGIFAALNDACATAHADSDAADQTFAQRLNMVAN-NPHFELRQQKFIIKHYAGDVTYDFHNMTDKNKDQMLKDLLEMIKTSSKQFLLS-LFPDAVD
OgpMyo1      481 K-RPPGIFAALNDACATAHADSSAADQSFAQRLNMVSS-NPHFELRSSKFIIKHYAGDVTYDIGTMTDKNKDQLLKDLLEMIGTSQNTFIQT-IFPEKID
DebMyo1      480 K-RPPGIFAALDDACATAHADSVAADQSFSQRLGMLDS-NPHFSSRGSKFVIRHYAGDVTYDIKHITDKNKDQLLNDLLEMIQTSQDPFVHT-LFPNKVD
PiuMyo1      481 K-RPPGIFAALNDACATAHADSSAADQSFAQRLNMLST-NQHFDLRGSKFVVKHYAGDVTYDIAAMTDKNKDQLLKDLLELLQTSQDPFIRE-LFPNSVD
NabMyo1B     474 K-RPPGIFAAMNDSVATAHADSNAADQAFSQRLTMFTS-NAHFELRQSKFVIKHYAGDVTYEINGITDKNKDQLQKDLVNLIVTSQSEFLKS-IFPTEID
ZbMyo1       474 R-RPPGIFAMMNDSVATAHADPSAADQAFAGRLNMFAS-NPHFEMRSSKFVIKHYAGDVTYDINGITDKNKDQLQKDLVDLMGTSENIFLKT-IFPDTSD
Ca_bMyo1     484 TRPQPGLFAALNDSIKTAHADSEAADQVFAQRLSMVGASNRHFGDRRGKFIIKHYAGDVTYDVAGMTDKNKDAMLRDLLELVSTSQNSFINQVLFPPDLL
StaMyo1      482 VRPQPGLFAALNDSIKTAHADSDAADQVFAQRLSMVGASNRHFEDRRGKFIIKHYAGDVTYDVAGMTDKNKDAMLRDLLELLGTSSNPFINQVLFPPGLL
ShpMyo1      480 VRPNPGLFAALNDSIKTAHADSEAADQVFAQRLSMVGASNRHFEDRRGKFIIKHYAGDVTYDVAGMTDKNKDAMLRDLLELLGTSSNQFITQILFPPGLL
CllMyo1      484 VRPQPGLFAALNDSIKTAHADSDAADQVFAQRLSMVGASNRHFEDRRGKFIIKHYAGDVTYEVAGMTDKNKDSMLRDLLELLSTSTNTFVTQSLFPPEML
MefMyo1      483 TRPQPGLFAALNDSIKTAHADSDAADQVFAQRLSMVGANNRHFEDRRGKFIIKHYAGDVTYEVAGMTDKNKDSMLRDLLELLSTSSNDFVTQSLFPPHLL
CnmMyo1      482 TRPQPGLFAALNDSIKTAHADSDAADQVFAQRLSMVGASNKHFEDRRGKFIIKHYAGDVTYDVAGMTDKNKDAMLKDLLEMLGTSNNPFITQVLFPPHLL
DhhMyo1      483 LRPQPGLFAALNDSVKTAHADSDAADQVFAQRLSMVGANNAHFEDRRGKFIIKHYAGDVTYDVAGMTDKNKDSMLRDLLEVLSTSSNSFVSQVLFPPELL
DehMyo1      483 LRPQPGLFAALNDSVKTAHADSDAADQVFAQRLSMVGANNAHFEDRRGKFIIKHYAGDVTYDVAGMTDKNKDSMLRDLVEVLSTSSNSFVSQVLFPPDLL
CatMyo1      483 TRPTPGLFAALNDSVKTAHADSDAADQVFSQRLGMVGSTSRHFEDRRGKFIIKHYAGDVTYEVSGMTDKNKDSMLRDLLEMLSTSKNSFISSVLFPPEML
LoeMyo1      489 TRPQPGLFAALNDSIKTAHADSDAADQVFAQRLSMVGASNRHFEDRRGKFIIKHYAGDVTYDVAGMTDKNKDAMLRDLLELLGTSSNAFVTQTLFPPNLL
MrgMyo1      481 TRPQPGLFAALNDSIKTAHADSDAADQVFAQRLSMVGANNRHFEDRKGKFIIKHYAGDVVYDVAGMTDKNKDAMLRDLLEMLSTSQNTFVNSVLFPPDLL
ShsMyo1      477 TRPQPGLFAALNDSIKTAHADSDAADQVFAQRLSMVGASNRHFEDRKGKFIIKHYAGDVTYEVAGMTDKNKDALLRDLVELLSTSANPFVNQVLFPPSLL
CameMyo1     483 TRPQPGLFAALNDSIKTAHADSDAADQVFAQRLSMVGANNRHFEDRRGKFIIKHYAGDVTYDVAGMTDKNKDGILRDLLELLGTSSNPFVNQTLFPPELL
MifMyo1Alpha 482 MKPHPGLFAALNDSVKTAHADSDAADQVFAQRLSMVGANNSHFEDRRGKFIIKHYAGDVTYDVAGMTDKNKDAMLRDLLEVLSTSTNGFVSKVLFPPELL
CadMyo1      485 TRPQPGLFAALNDSIKTAHADSEAADQVFAQRLSMVGASNRHFEDRRGKFIIKHYAGDVTYDVAGMTDKNKDAMLRDLLELVSTSKNTFINQILFPPDLL
CaoMyo1      483 TRPQPGLFAALNDSIKTAHADSDAADQVFAQRLSMVGANNRHFEDRRGKFIIKHYAGDVTYDVAGMTDKNKDGILRDLLELLGTSSNPFVNQTLFPPELL
CapMyo1      483 TRPQPGLFAALNDSIKTAHADSDAADQVFAQRLSMVGANNRHFEDRRGKFIIKHYAGDVTYDVAGMTDKNKDGILRDLLELLGTSSNPFVNQTLFPPELL
Ct_aMyo1     481 TRPQPGLFAALNDSIKTAHADSDAADQVFAQRLSMVGASNKHFEDRRGKFIIKHYAGDVTYDVAGMTDKNKDAMLRDLLELLGTSNQSFITQVLFPPELL
PtaMyo1      480 --KRGGIFSLLNDACATAHADPSAADQSFAQRLNMSSG-NKYFELRSNKFIVHHYAGDVTYDIDGITDKNKDSMLKDLVEMLSTSTNTFITENIVEKIDT
CacaMyo1B    477 K-RPPGIFAAMNDAIATAHADSDAADQVFAQRLNIFNS-NPYFEMRQNKFVIKHYAGDVTYNVDSITDKDKDQLQKDLVELISTTSNTFLSS-IFPEPVD
NadMyo1      474 R-RPPGIFAAMNDSVATAHADSNAADQAFSQRLNLFST-NPHFELRSNKFVIKHYAGDVTYDVNGITDKNKDQLQKDLVELMGTTTNTFLST-IFPMEVD
```

```
                         610       620       630       640       650       660       670       680       690       700
                ....|....|....|....|....|....|....|....|....|....|....|....|....|....|....|....|....|....|....|....|
CnbMyo1A      571 R---DNKKRPPTAGDKIIKSANELVETLSKAQPSYIRTIKPNQTKSPNDYDDHQVLHQVKYLGLQENVRIRRAGFAYRQGFDKFVERFYLLSPRCSYAGD
CdnMyo1A      571 R---DNKKRPPTAGDKIIKSANELVETLSKAQPSYIRTIKPNQTKSPNDYDDHQVLHQVKYLGLQENVRIRRAGFAYRQGFDKFVERFYLLSPRCSYAGD
CacaMyo1A     572 K---TSKRRPPSAGDRMIQSANDLVDTLAKAQPSYIRTIKPNQTKSPNDYDDKQVLHQVKYLGLQENVRIRRAGFAYRQIYEKFVERFYLLSPRCSYAGE
NabMyo1A      571 K---DSKKRPPSAGDKIITSANDLVDTLSKARPSYIRTIKPNQTKSPNDYDDKQVLHQVKYLGLQENVRIRRAGFAYRQLFDKFVERFYLLSPRCSYAGD
NdMyo1A       571 R---DSKKRPPTAGDKIIKSANELVDTLSKAQPSYIRTIKPNQTKSPNDYDDHQVLHQVKYLGLQENVRIRRAGFAYRQSFDKFVERFYLLSPRCSYAGD
CnbMyo1B      571 K---DSRKRPPTSGDKIIKSANELVETLSKAQPSYIRTIKPNQTKSPKDYDDHQVLHQIKYLGLKENVRIRRAGFAYRQTFEKFVERFYLLSPQCSYAGD
CglMyo1A      572 K---DNKRRPPTAGDKIIKSANELVETLSKAQPSYIRTIKPNQTKSPNDYDDHQVLHQVKYLGLQENVRIRRAGFAYRQGFEKFVERFYLLSPRCSYAGD
Sab_aMyo1B    571 K---ESKRRPPTAGDKIIKSANELVETLSKAQPSYIRTIKPNETKSPNDYDDRQVLHQIKYLGLQENVRIRRAGFAYRQVFEKFVERFYLLSPHCSYAGD
Sc_cMyo1B     571 R---ESKRRPPTAGDKIIKSANDLVETLSKAQPSYIRTIKPNETKSPNDYDDRQVLHQIKYLGLQENVRIRRAGFAYRQVFEKFVERFYLLSPHCSYAGD
SaaMyo1B      571 R---ESKRRPPTAGDKIIKSANELVETLSKAQPSYIRTIKPNETKSPNDYDDRQVLHQIKYLGLQENVRIRRAGFAYRQVFEKFVERFYLLSPHCSYAGD
SakMyo1B      571 R---ESKRRPPTAGDKIIKSANELVETLSKAQPSYIRTIKPNETKSPNDYDDRQVLHQIKYLGLQENVRIRRAGFAYRQVFDKFVERFYLLSPHCSYAGD
SmiMyo1B      571 R---DSKRRPPTAGDKIIKSANDLVETLSKAQPSYIRTIKPNETKSPNDYDDRQVLHQIKYLGLQENVRIRRAGFAYRQVFEKFVERFYLLSPHCSYAGD
Sap_aMyo1B    571 R---ESKRRPPTAGDKIIKSANDLVETLSKAQPSYIRTIKPNETKSPNDYDDRQVLHQIKYLGLQENVRIRRAGFAYRQVFEKFVERFYLLSPHCSYAGD
NacMyo1       571 K---ESKRRPPTAGDKIIKSANELVETLSKAQPSYIRTIKPNQTKSPNDYDDHQVLHQVKYLGLQENVRIRRAGFAYRQTFEKFVERFYLLSPHCSYAGD
KaaMyo1       571 K---ESKRRPPTAGDKIIKSANELVETLSKAQPSYIRTIKPNQTKSPNDYDDQQVLHQIKYLGLQENVRIRRAGFAYRQTYDKFVERFYLLSPHCSYAGD
VpMyo1        571 K---DSKRRPPTAGDKIIKSANELVETLSKAQPSYIRTIKPNQTKSPNDYDDQQVLHQVKYLGLKENVRIRRAGFAYRQVFDKFVERFYLLSPSCSYAGD
TtpMyo1A      571 T---ENKRKAPTAGDKIIKSANDLVETLSKAQPSYIRTIKPNQTKLPEDYDDSQVLHQVKYLGLQENVRIRRAGFAYRQVFDKFVERFYLLSPQCSYAGD
TtpMyo1B      571 T---ENKRKAPTAGDKIIKSANDLVETLSKAQPSYIRTIKPNQTKLPEDYDDSQVLHQVKYLGLQENVRIRRAGFAYRQVFDKFVERFYLLSPQCSYAGD
TtbMyo1A      578 H---ESRRRPPTAGDKIIKSANELVETLSKSSPSYIRTIKPNQTKSPKDYDDHQVLHQVKYLGLKENVRIRRAGFAVRQTFEKFVERFYLLSPDCSYAGE
TtbMyo1B      573 K---ESKRRPPTAGDKIIKSANELVETLSKASPSYIRTIKPNQTKVPTDYDDNQVLHQVKYLGLKENVRIRRAGFAYRQTFDKFVERFYLLSPACSYAGD
ErgMyo1       570 K---DSKRIPSTAGDKIIRSANELVDTLSKAQPSYIRTIKPNQTKSPLDYDDRQVLHQVKYLGLQENVRIRRACYAYRHIFDKFVERFYLLSPQCSYAGD
ErcMyo1       571 R---TSKKRPPTAGDKIIKSANELVDTLSKSQPSYIRTIKPNQTKSPNDYDDQQILHQIKYLGLQENVRIRRAGFAYRQTFDKFVERFYLLSPMCSYAGD
HsvMyo1       570 T---ESKRRPPTAGDKIITSANALVETLSKTTPSYIRTIKPNQTKRPEDYDEKQVLHQIKYLGLQENVRIRRSGFAYRQTFEKFVERFYLLSPKCSYAGD
KlMyo1        569 K---SSKRRPPTAGNKIIKSANELVETLSKAQPSYIRTIKPNQTKSPRDYDDQQVLHQVKYLGLQENVRIRRAGFAYRQTFEKFVERFYLLSPKCSYAGD
KmmMyo1       569 K---SSKKRPPTAGTKIIKSANELVDTLSKAQPSYIRTIKPNQTKSPRDYDDQQVLHQVKYLGLQENVRIRRAGFAYRQTFEKFVERFYLLSPQCSYAGD
KlwMyo1       569 R---SSKRRPPTAGTKIIKSANELVDTLSKSQPSYIRTIKPNQTKSPKDYDDQQVLHQVKYLGLQENVRIRRAGFAYRQTFEKFVERFYLLSPKCSYAGD
KaMyo1        570 K---NSKKRPPTAGDKIIKSANELVDTLSKAQPSYIRTIKPNQTKSPNDYDDQQVLHQIKYLGLQENVRIRRAGFAYRQTFDKFVERFYLLSPQCSYAGD
KnMyo1        571 K---DSKKRPPTAGDKIIKSANELVETLSKAEPSYIRTIKPNETKSPTDYDNHRVLHQVKYLGLQENVRIRRAGFAYRQTFDKFVERFYLLSPHCSYAGD
LwMyo1        571 K---NSKRRPPSAGDKIIKSANELVETLSKAQPSYIRTIKPNQTKSPNDYDDHQVLHQVKYLGLQENVRIRRAGFAYRQVFDKFVERFYLLSPQCSYAGD
LatMyo1       571 K---NSKRRPPSAGDKIIKSANELVETLSKAQPSYIRTIKPNQTKSPNDYDDHQVLHQVKYLGLQENVRIRRAGFAYRQTFDKFVERFYLLSPQCSYAGD
Lak_aMyo1     575 K---GSKRRPPTAGDKIITSANELVDTLSKASPSYIRTIKPNQTKSPNDYDDHQVLHQVKYLGLQENVRIRRAGFAYRQTFEKFVERFYLLSPKCSYAGD
Sab_aMyo1A    571 K---DSKRRPPTAGDKIIKSANELVETLSKAEPSYIRTIKPNQTKSPDDYDDHQVLHQVKYLGLQENVRIRRAGFAYRQTFEKFVERFYLLSPDCSYAGD
Sc_cMyo1A     570 K---DSKRRPPTAGDKIIKSANELVETLSKAEPSYIRTIKPNQTKSPNDYDDHQVLHQVKYLGLQENVRIRRAGFAYRQTFEKFVERFYLLSPDCSYAGD
SaaMyo1A      571 K---DSKRRPPTAGDKIIKSANELVETLSKAEPSYIRTIKPNQTKSPDDYDDHQVLHQVKYLGLQENVRIRRAGFAYRQTFEKFVERFYLLSSDCSYAGD
SakMyo1A      556 K---DSKRRPPTAGDKIIKSA-------------------------------------------------------------------------------
SmiMyo1A      571 K---DSKRRPPTAGDKIIKSANELVETLSKAEPSYIRTIKPNQTKSSDDYDDHQVLHQVKYLGLQENVRIRRAGFAYRQTFEKFVERFYLLSPDCSYAGD
Sap_aMyo1A    571 K---DSKRRPPTAGDKIIKSANELVETLSKAEPSYIRTIKPNQTKSPDDYDDHQVLHQVKYLGLQENVRIRRAGFAYRQSFEKFVERFYLLSPDCSYAGD
ZrMyo1        571 K---TSKRRPPTAGDRIIKSANELVDTLSKAQPSYIRTIKPNQTKSPQDYDDHQVLHQVKYLGLQENVRIRRAGFAYRQVFDKFVERFYLLSPRCSYAGE
TodMyo1       571 R---ESKKRPPTAGDKIIKSANELVETLSKAQPSYIRTIKPNQTKSPNDYDDAQVLHQVKYLGLQENVRIRRAGFAYRQLFDKFVERFYLLSPRCSYAGD
CglMyo1B      571 T---DSRRRPPTSGDKIIKSANELVETLSKAQPSYIRTIKPNDTKSSTIYDDQRVLHQIKYLGLKENVRIRRAGFAHRQVFEKFVERFYLLSPQCSYAGD
WaMyo1Alpha   571 Q---ESKRRPPTAGDKIIKSANELVDTLSKSSPSYIRTIKPNQNRSPTEYDEKQVLHQVKYLGLQENVRIRRAGFAYRQTFEKFAERFYLLSPQCSYAGD
WicMyo1       571 Q---NSKRRPPTAGDKIIKSANELVDTLSKASPSYIRTIKPNQNRSPSEYDTKQVLHQVKYLGLQENVRIRRAGFAYRQTFDKFSERFYLLSPKCSYAGD
CyjMyo1       563 H---GSRRRPPTASDKIIKSANELVETLSKPSYIRTIKPNEHRSASEFDDRQVLHQIKYLGLKENVRIRRAGFAYRQTFEKFVERFYLLSRKCSYAGE
YlMyo1        572 T---DNRRRPPTASDKIKKSANDLVTTLSAAQPSYIRTIKPNQNRSPTEYDEKAVLHQVKYLGLQENVRIRRAGFAYRQTFEKFVERFMLLSGKTSYAGD
CdnMyo1B      571 K---DSRRKPPTSGDKIIKSANELVETLSKAQPSYIRTIKPNQTKSPNDYDNHQVLHQVKYLGLKENVRIRRAGFAYRQTFDKFVERFYLLSPECSYAGD
Kop_bMyo1     573 TNAGGRKNRPTTASAKIKTSANLLVDTLSQAQPSYIRTIKPNQNKSPTEYDDRAVLHQVKYLGLQENVRIRRAGFAYRQTFEKFSERFYLLSTKTSYAGD
NdMyo1B       570 K---DSRRKPPTSGDKIIKSANELVETLSKAQPSYIRTIKPNQTKSPNDYDDHQVLHQVKYLGLKENVRIRRAGFAYRQTFDKFVERFYLLSPECSYAGD
KcMyo1        574 T---NSKKRPPTAGDKIKTSANLLVETLSQASPSYIRTIKPNQNRSPSEYDTKAVLHQVKYLGLQENVRIRRAGFAYRQTFEKFAERFHLLSTKTSYAGE
OgpMyo1       578 T---SSKKRPPTAGDKIKTSANLLVDTLSQAQPSYIRTIKPNQNRSPTEYDTKAVLHQVKYLGLQENVRIRRAGFAYRQTFDKFVERFYLLSTRTSYAGE
DebMyo1       577 E---NNKKRPPTASQKIKKSANDLMTTLSRATPSYIRTIKPNDHRSATEYDVSHVLHQIKYLGLKENVRIRRAGFAYRQTFEKFVERFYLLSSQTSYAGE
PiuMyo1       578 Q---SSKKRPPTAGDKIKTSANLLVDTLSQATPSYIRTIKPNQHRSPTEYDTKAVLHQVKYLGLKENVRIRRAGFAYRQTFEKFVERFYLLSTKTSYAGE
NabMyo1B      571 Q---GSKRRPPTAGDKIIKSANELVETLSKAQPSYIRTIKPNETKSPKDYNDQQTLHQIKYLGLRENVRIRRAGFAYRQTFEKFVERFYLLSNKLFYAGD
ZbMyo1        571 K---TSKRRPPTAGDKIIKSANELVETLSKAQPSYIRTIKPNQTKSPNDYDDQQVLHQVKYLGLQENVRIRRAGFAYRQVFDKFVERFYLLSPRLSYAGE
Ca_bMyo1      584 AQLTDSRKRPETASDKIKKSANILVDTLSQCTPSYIRTIKPNQTKKPRDYDNQQVLHQIKYLGLKENVRIRRAGFAYRSTFERFVQRFYLLSPATGYAGD
StaMyo1       582 AALTDSKKRLETASDKIKTSANALVDTLSKCSPSYIRTIKPNQSKKPRDYDNHQVLHQIKYLGLKENVRIRRAGFAYRSTFERFVQRFYLLSPQTGYAGD
ShpMyo1       580 AALTDSKKRLETASDKIKTSANKLVDTLSKCSPSYIRTIKPNQSKKPRDYDNHQVLHQIKYLGLKENVRIRRAGFAYRSTFERFVQRFYLLSPQTGYAGD
CllMyo1       584 AAVTDSKKRPETASDKIKRSANLLVDTLSQCQPSYIRTIKPNQTKRPKEYDNQQVLHQIKYLGLKENVRIRRAGFAYRTTFDKFVQRFYLLSPATGYAGD
MefMyo1       583 QALTDSKKRPDTASDKIKKSANILVDTLSQCQPSYIRTIKPNQTKRPKEYDNQQVLHQIKYLGLKENVRIRRAGFAYRTTFDKFVQRFYLLSPATGYAGD
CnmMyo1       582 AALTDSKKRPESASDKIKKSANLLVDTLSQCTPSYIRTIKPNQTKKPRDYDNQQVLHQIKYLGLKENVRIRRAGFAYRSTFERFVQRFYLLSPATGYAGD
DhhMyo1       583 AALTDSKKRPETASDKIKKSANLLVDTLSQCQPSYIRTVKPNQTKRPKEYDNNQVLHQIKYLGLKENVRIRRAGFAYRTTFDKFVQRFYLLSPKTGYAGD
DehMyo1       583 AALTDSKKRPETASDKIKKSANLLVDTLSQCQPSYIRTIKPNQTKRPKEYDNNQVLHQIKYLGLKENVRIRRAGFAYRTTFDKFVQRFYLLSPKTGYAGD
CatMyo1       583 TALLDNKKRLETASDKIKKSANLLVDTLSQCQPSYIRTIKPNQTKRPKEYDNAQVLHQVKYLGLKENVRIRRAGFAYRTTFDKFVQRFYLLSSKTGYAGD
LoeMyo1       589 SELVDSKKRPETASDKIKKSANILVDTLSQATPSYIRTIKPNQTKKPRDYDNQQVLHQIKYLGLKENVRIRRAGFAYRSTFQRFVQRFYLLSPATGYAGD
MrgMyo1       581 AVLTDKKKRPETASDKIKKSANLLVDTLSQCQPSYIRTIKPNQTKRPKEYDNAQVLHQVKYLGLKENVRIRRAGFAYRTTFDKFVQRFYLLSPKTGYAGD
ShsMyo1       577 ASITDSKKRPETASDKIKRSANDLVGTLSKCQPSYIRTIKPNQTKRPKEYENQQVLHQIKYLGLKENVRIRRAGFAFRTTFEKFVQRFYLLSPATGYAGD
CameMyo1      583 NALIDTKKRPPTASDKIKTSANALVDTLSQCTPSYIRTIKPNQSKKPRDYDNQQVLHQIKYLGLKENVRIRRAGFAYRSTFQRFVQRFYLLSPATGYAGD
MifMyo1Alpha  582 ASLTDSKKRPESASDKIKRSANLLVDTLSQCQPSYIRTIKPNQTKRPKEYDNHQVLHQVKYLGLKENVRIRRAGFAYRTTFEKFVQRFYLLSPKTGYAGD
CadMyo1       585 SILTDSRKKPETASDKIKKSANILVDTLSQCTPSYIRTIKPNQTKKPHDYDNQQVLHQIKYLGLKENVRIRRAGFAYRSTFERFVQRFYLLSSTTGYAGD
CaoMyo1       583 NALIDTKKRPPTASDKIKTSANALVDTLSQCTPSYIRTIKPNQTKKPRDYDNQQVLHQIKYLGLKENVRIRRAGFAYRSTFQRFVQRFYLLSPATGYAGD
CapMyo1       583 NALIDTKKRPPTASDKIKTSANALVDTLSQCTPSYIRTIKPNQTKKPRDYDNQQVLHQIKYLGLKENVRIRRAGFAYRSTFQRFVQRFYLLSPATGYAGD
Ct_aMyo1      581 STLTDSKKRPETASDKIKRSANLLVDTLSQCAPSYIRTIKPNQTKKPRDYDNQQVLHQIKYLGLKENVRIRRAGFAYRSTFERFVQRFYLLSPVTGYAGD
PtaMyo1       577 ----NSKRRPPSGGDKIRQSANALVEKLSQCTPSYIRTIKPNENKAPLDYNVKNVLHQIKYLGLKENVKVRKAGFSYRSTFEQFVQRFFLLSPYTCYAGE
CacaMyo1B     574 K---SSRKRPTTAGDKIIKSANDLVDTLSKAQPSYIRTIKPNETKSPKDYDDQRVLHQVKYLGLQENVRIRRAGFAYRQTFEKFVESFYLLSPSLSYAGD
NadMyo1       571 K---ESKRRPPTAGDKIIKSANELVETLSKAQPSYIRTIKPNETKSPNDYDDHQVLHQVKYLGLQENVRIRRAGFAYRQVFDKFVERFYLLSPHCSYAGD
```

```
                           710       720       730       740       750       760       770       780       790       800
                  ....|....|....|....|....|....|....|....|....|....|....|....|....|....|....|....|....|....|....|....|
CnbMyo1A      668 YTWTGETLEAVKLILQDAMIPDKEYQLGVTQVFIKTPETLFALENMRDKFWHNMAARIQRAWRRYLQRRIDAAIKIQRTIKERKEGNK----FEQVRDYG
CdnMyo1A      668 YTWTGETLDAVKYILQDALIPEKEYQLGVTQVFIKTPETLFALENMRDKFWHNMAARIQRAWRRYLQRRIDAAVRIQRTIKERKEGNK----YEQLRDYG
CacaMyo1A     669 YTWQGETLAAVKYILEDARIPQKEFQLGVTSVFIKTPETLFALEGMRDKFWHNMAARIQRAWRRFLLRRVDAAIKIQRTIKERKIGNK----YEQLRDYG
NabMyo1A      668 YTFQGETLDAVRYILEDASIPGKEYQLGVTSVFIKTPETLFALENMRDKFWHNMAARIQRAWRRFLQRRIDSAIKIQRVIRERKEGNK----FEKLRNGG
NdMyo1A       668 YTWTGETLDAVRYILQDALIPEKEYQLGVTQVFVKTPETLFALENMRDKFWHNMAARIQRAWRRYLQRRIDAAIRIQRTIKERKEGNK----YEQLRDYG
CnbMyo1B      668 YIWDGETLDAVKLILQDASIPQSEYQVGVTQVFIKQPETLFALENMRDRYWYNMAARIQRACRRYLQKRIDAAIKIQNAFRGKSGVGSFR--NDELRNAG
CglMyo1A      669 YTWTGDILEAVRLILQDALIPEKEYQLGVTQVFIKTPETLFALENMRDKFWHNMAARIQRAWRRYLQRRIDAAVKIQRTIKERKEGNK----FEKLRDYG
Sab_aMyo1B    668 YTWQGDTLDAVKYILQDSSIPQQEYQLGVTSVFIKTPETLFALEHMRDKYWYNMAARIQRAWRRFLQRRIDAAIKIQRTIRERKGGNK----YEKLRDYG
Sc_cMyo1B     668 YTWQGDTLDAVKYILQDSSIPQQEYQLGVTSVFIKTPETLFALEHMRDRYWHNMAARIQRAWRRFLQRRIDAATKIQRTIRERKEGNK----YEKLRDYG
SaaMyo1B      668 YTWQGDTLDAVKYILQDASIPQQEYQLGVTSVFIKTPETLFALEHMRDKYWYNMAARIQRAWRRFLQRRIDAAIKIQRTIRERKGGNK----YEKLRDYG
SakMyo1B      668 YTWQGDTLNAVKFILKDSSIPQQEYQLGVTSVFIKTPETLFALEHMRDRYWYNMAARIQRAWRRFLRRRIDAAIKIQRTIRERKEGNK----YEKLRDYG
SmiMyo1B      668 YTWQGETLDAVKYILQDSSIPQQEYQLGVTSVFIKTPETLFALEHMRDRYWHNMAARIQRAWRRFLQRRIDAATKIQRTIRERKEGNK----YEKLRDYG
Sap_aMyo1B    668 YTWQGDTLDAVKYILQDSSIPQQEYQLGVTSVFIKTPETLFALEHMRDKYWHNMAARIQRAWRRFLQRRIDAATKIQRTIRERKEGNK----YEKLRDYG
NacMyo1       668 YTWQGETLDAVQHILNDASIPASEYQLGTTSVFIKTPETLFALEHMRDRYWHNMAARIQRAWRRFLRRRIDAAIRIQRTIRERKSGNK----YEQLRDEG
KaaMyo1       668 YTWQGDTLDAVKYILLDASVPEKEYQLGVTSVFIKTPETLFALEHMRDRYWHNMAARIQRAWRRFLQRRIDSAIRIQRTIRERKEGNK----YEKLRDYG
VpMyo1        668 YTWQGETLEAVKLILKEASIPEKEYQVGVSQVFIKTPETLFALEHMRDKYWYNMAARIQRAWRRFLQRRIDSATRIQRAIREKKGGNK----YEKLRDEG
TtpMyo1A      668 YTWQGETIDAVKWILKDASIPEKEYQIGVSQVFIKTPETLFALEHMRDRYWHNMAARIQRAWRRFLQRRIDSAVKIQRVIRERKGGNK----YEKLRDKG
TtpMyo1B      668 YTWQGETIDAVKWILKDASIPEKEYQIGVSQVFIKTPETLFALEHMRDRYWHNMAARIQRAWRRFLQRRIDSAVKIQRVIRERKGGNK----YEKLRDKG
TtbMyo1A      675 YTWDGPVEEAVKLILRDTSTPQKEYQMGVTCVFIKNPETYFTFEAMRDRYWYNMAAKIQRALRKHLQKRLDSAIKIQRAIRGQANGGLGRG-DVDMREYS
TtbMyo1B      670 YTFQGEPYDAIKYILRDASIPQQEYQLGTTSVFIKNPETLFALENMRDKYWYNMAARIQRAWRRFNVRRVDAASRIQRAIRERKGGNN----FEQLRDFG
ErgMyo1       667 YTWQGNTLDAVNLILRDTSIPVTEYQLGVTKVFIKTPETLFALENMRDKYWHNMASRIQRAWRRFLQRRIDSAIRIQRAIREMKHGNQ----FEQLRDYG
ErcMyo1       668 YTWHGETLDAVKQILKDTSIPTKEYQLGVSKVFIKTPETLFALEHMRDKYWHNMAARIQRAWRRFLQRRIDSAMRIQRAIREKKHGNK----FEKLRDYG
HsvMyo1       667 YIWQGDEIEACNQILKDARIPVSEYQVGVTKVFIKTPETLFALESMRDKYWHNMAARIQRAWRRFLQRRIDAAASIQRAIREKKSGNK----YVQLRDYG
KlMyo1        666 YTWQGDTLGAVKQILQDASIPTTEYQLGVTKVFIKTPETLFALEHMRDRYWYNMAARIQRAWRRFIQRRIDSAIKIQRAIREKKGGNQ----YEQLRDYG
KmmMyo1       666 YTWQGDTLGAVKQILKDASIPTTEYQLGVTKVFIKTPETLFALEHMRDKYWYNMAARIQRAWRRFIQRRIDSAIKIQRAIKEKKGGNQ----YEQLRDYG
KlwMyo1       666 YTWQGDTLGAVKQILQDASIPAAEFQLGVTKVFIKTPETLFALEHMRDKYWYNMAARIQRAWRRFIQRRIDSAVRIQRAIREKKGGNQ----YEQLRDYG
KaMyo1        667 YTWQGSTLDAVTQILKDSSIPTGEYQLGVSKVFIKTPETLFALEHMRDRYWYNMAARIQRAWRRFIQRRIDSATKIQRAIREKKGGNQ----YEQLRDYG
KnMyo1        668 YTWQGETLDAVKHILIDTAIPQKEYQLGTTSVFIKTPETLFALEHMRDRYWHNMAARIQRAWRRFLQRRIDAAIRIQRIIREKKDGNK----YEKLRDYG
LwMyo1        668 YTWQGDTLEAVKLILKDASIPPTEYQLGVSKVFIKTPESLFALETMRDKYWHNMAARIQRAWRRFLLRRIDAASRIQRAIREKKGGNE----FEKLRDYG
LatMyo1       668 YIWQGDTLDAVKLILKDASIPPSEYQLGVSKVFIKTPESLFALETMRDKYWYNMAARIQRAWRRFLLRRVDAASRIQRAIREKKGGNK----YEKLRDSG
Lak_aMyo1     672 YTWQGETLEAVRHILSDASIPTTEYQLGVSKVFIKTPETLFALEHMRDKYWYNMAARIQRAWRRFLQRRIDAAARIQRAIREKKGGNK----YEQLRDYG
Sab_aMyo1A    668 YTWEGDTLEAVKLILRDALIPEKEFQLGVTSVFIKTPESLFALEDMRDKYWYNMAARIQRAWRRFLQRRVDAAIKIQRTIREKKGGNK----YEKLRDYG
Sc_cMyo1A     667 YTWDGDTLEAVKLILRDAMIPEKEFQLGVTSVFIKTPESLFALEDMRDKYWYNMAARIQRAWRRFLQRRIDAAIKIQRTIREKKGGNK----YVKLRDYG
SaaMyo1A      668 YTWEGDTLEAVRLILRDAFIPEKEFQLGVTSVFIKTPESLFALEDMRDKYWYNMAARIQRAWRRFFQRRIDAAIKIQRTIREKKGGNK----YEKLRDYG
SakMyo1A      573 ----------------------------------------------------------------------------------------------------
SmiMyo1A      668 YTWEGDTLEAVKLILRDAMIPEKEFQLGVTSVFIKTPESLFALEDMRDKYWYNMAARIQRAWRRFLQRRIDAAIKIQRTIREKKGGNK----YVKLRDYG
Sap_aMyo1A    668 YTWEGDTLEAVKLILRDAMIPEKEFQLGVTSVFIKTPESLFALEDMRDKYWYNMAARIQRAWRRFLQRRIDAAIKIQRIIREKKGGNK----YVKLRDYG
ZrMyo1        668 YTWEGDVLEAVDMILQDAAIPQKEFQLGVTSVFIKTPETLFALENMRDRFWHNMAARIQRAWRRFNQKRVDSAIKIQRAVREKSSGNE----FEQFRDQG
TodMyo1       668 YTWQGDTLEAVKMILVDASIPEKEYQLGVTQVFIKTPETLFALEHMRDRYWYNMAARIQRAWRRFRQRRIDAAVRIQRAIKEKKGGNK----YEQLRDYG
CglMyo1B      668 YVWDGETLDAVKLILQDASIPTTEYEIGVTQIFIKHPETLFALENMRDKYWYNMAARIQRAWRRYLQKRIDAAIRIQNAIRGKSGVSTFR--NDELRNAG
WaMyo1Alpha   668 YIWQGDSRSAVLQILKDAAIPASEYQMGVTKVFIKTPETLFGLESLRDRYWYNMAARIQRAWRRYLQRRIDCAIRIQRFWRERTGGNQ----FEQLRDYG
WicMyo1       668 YIWEGDSKTGVVQILKDAGIPSSEYQMGVSKVFIKTPETLFGLESLRDRYWYNMAARIQRAWRRYQKRKIDSAIKIQRFWREKTGGNQ----YEQLRDFG
CyjMyo1       660 FTWSGDIKDAVLHILQDSAIPATEYQMGTTKVFIKTPETLFGLESLRDRYWHNMAAIIQRAIRRYMKKKVDAAIIIQRIWRERTGGND----YEKFRDYG
YlMyo1        669 YIWQGSAYDATLCILRDAGIPQTEYQMGTTKVFIKTPETLFALEHMRDMWWHNMAARIQRAWRRYLAYKTECAIKIQRFWRLKRGLDGLK--EIQFRDSG
CdnMyo1B      668 YIWDGETLDAVKMILRDASIPQSEYQVGVTQVFVKQPETLFALENMRDKYWYNMAARIQRGIRRYLQKRIDAAIKIQNAFRGKSGVGSFR--NDELRNAG
Kop_bMyo1     673 YIWEGDHKSACTVILKDAGIPETEWQVGVTKIFIKKPETLFSLESMRENYWANKAKVIQRAIRRWFKVRVDAAKTLQRIIREKKGGNV----YEQLRDYG
NdMyo1B       667 YVWDGETLDAVKMILRDASIPQSEYQVGVTQVFIKQPETLFALENMRDKYWYNMAARIQRGVRRYLQKRIDAAIKIQNAFRGKSGVSSFR--NDELRDAG
KcMyo1        671 YVWQGDARSAVSVILQDANIASSEWQLGTTKVFIKKPETLFSLEEMRENWWANKARVIQRACRRYLFQRWESAKLIQKTWREYKGGNK----FEKLRDYG
OgpMyo1       675 YIWDGDAKTATTLILQDTGIPNTEWQLGVTKVFIKKPETLFSLEEMRENYWANKAKVIQRACRRWLKRRENAAKFVQKMWRERVGGNK----YEQLRDYG
DebMyo1       674 YIYQGDAKTATEIILKDAGFPGTEWQLGVTKVFIKKPETLFALEDKRENYWASKAKIIQRACRRYLRRRERAARIVQRLWRARITGEADK--YIALRDYG
PiuMyo1       675 YIWTGDARTATEIILRDSNFDPSEWQLGVSKVFIKKPETLFALEDLRENYWANKAKVIQRACRKWLRRREEAAKIVQRLWRIRNKGFQETNQYIELRDFS
NabMyo1B      668 YTWQGDTSDAVKYILKDAAVKEKEFQLGVTSIFIKTPETLFELEHKRDKFWHTMATRIQRAWRKHYKKKLDVILLIQRRHRERKGGNK----YHKLRDTG
ZbMyo1        668 YTWDGDVLEAVKMILADAAIPQKEYQLGVTSVFIKTPETLFALEHMRDRFWYNMAARIQRAWRRFNQKRVDAAIKIQRAAKERNTGNP----YEKLRDQG
Ca_bMyo1      684 YIWRGDDISAVKEILKSCHIPPSEYQLGTTKVFIKTPETLFALEDMRDKYWHNMAARIQRAWRRYVKRKEDAAKTIQNAWRIKKHGNQ----FEQFRDYG
StaMyo1       682 YIWRGDDISAVKEILRACYIPSGEFQMGTSKIFIKTPETLFALEDMRDKYWHNMATRIQRAWRRYVKRKEDAAKTIQQAWRIKKHGNQ----FEQLRDYG
ShpMyo1       680 YIWRGDDISAVKEILRACYIPATEFQMGTSKVFIKTPETLFALEDMRDKYWHNMATRIQRAWRRYVKRKEDAAKTIQRAWRVKKHGNQ----FEQLRDYG
CllMyo1       684 YIWQGDDITAVKEILKSCYIPDTEYQMGTSKVFIKTPETLFALEDMRDKYWHNMAARIQRAWRRYLKRKEDAAKVIQNAWRVKKHGNK----FEQFRDEG
MefMyo1       683 YIWRGDDITAVKEILRSCHIPPSEYQMGTTKVFIKTPETLFALEDMRDKYWHNMAARIQRAWRRYLKRKDDAAKVIQNAWRTKKHGNK----FEQIRDEG
CnmMyo1       682 YIWQGDDISAVKEILRACHIPNTEYQLGTTKVFIKTPETLFALEDMRDKYWHNMAARIQRAWRRYIKRKEDAAKTIQNAWRIKKHGNQ----FEQFRDYG
DhhMyo1       683 YIWNGDDVTAVHEILRSCHIPDSEYQMGTTKVFIKTPETLFALEDMRDKYWHNMAARIQRAWRRYIKRKDDAARLIQSAWKNKTHGNQ----FEQLRDYG
DehMyo1       683 YIWQGDDVTAVHEILRSCHIPDSEYQMGTTKVFIKTPETLFALEDMRDKYWHNMAARIQRAWRRYIKRKDDAARLIQSAWKNKTHGNQ----FEQLRDYG
CatMyo1       683 YIWRGDDISAVKEILKACFIPDSEYQMGTTKVFIKTPETLFALEDMRDKYWHNMAARIQRAWRRYLKRKEDAAIVMQRAWRNKTHGNK----FEQFRDYG
LoeMyo1       689 YIWQGDDLTAVKEILRACHIPTSEYQLGTTKVFIKTPETLFALEDMRDKYWHNMAARIQRAWRRYIKRKEDAAKTIQRAWRMKNHGNQ----FEQFRDYG
MrgMyo1       681 YIWNGDDISAVREILKSCHIPDTEFQMGTSKVFIKTPETLFAMEDMRDKYWHNMAARIQRAWRRYVKRKEDAARLIQNAWKVKKHGNQ----FEQLRDYG
ShsMyo1       677 YIWRGDDISAVKEILKSCFIPATEYQLGTTKVFIKTPETLFGLEDMRDKYWHNMAARIQRAWRRYVKRKEDAARTIQSAWRIKKHGNQ----FEQLRDYG
CameMyo1      683 YIWQGDDLTAVKEILRSCHIPSTEYQLGTTKVFIKTPETLFALEDMRDKYWHNMAARIQRAWRRYVKRKEDAAKTIQRAWKMKTHGNQ----FEQLRDYG
MifMyo1Alpha  682 YIWTGDDISAVKEILRSCFIPESEFQLGTTKVFIKTPETLFGLEDMRDKYWHNMAARIQRAWRRYLKRKEDAAKTMQRAWKNKTHGNQ----FEQLRDYG
CadMyo1       685 YIWRGDDISAVKEILKSCHIPSSEYQLGTTKVFIKTPETLFALEDMRDKYWHNMAARIQRAWRRYVKRKEDAAKTIQNAWRIKKHGNQ----FEQFRDYG
CaoMyo1       683 YIWQGDDLTAVKEILRSCHIPQSEYQLGTTKVFIKTPETLFALEDMRDKYWHNMAARIQRAWRRYVKRKEDAAKVIQNAWRMKTHGNQ----YEQLRDYG
CapMyo1       683 YIWQGDDLTAVKEILRACHIPQTEYQLGTTKVFIKTPETLFALEDMRDKYWHNMAARIQRAWRRYVKRKEDAAKIIQNAWRMKTHGNQ----YEQLRDYG
Ct_aMyo1      681 YIWQGDDISAVKEILKACFIPASEYQLGTTKVFIKTPETLFALEDMRDKYWHNMAARIQRAWRRYVKRKEDAAKVIQNAWRMKTHGNQ----FEQFRDYG
PtaMyo1       674 YIHQGDTKEAIMHILHSANVPIDEYKIGLTKIFIKSPQTIFGLEDLKDKYFDTMAIRIQRAWRKRIQKKIDAAVVLQKSWRIHKKGNK----FEQLRDYG
CacaMyo1B     671 YVWEGDIIQATKLILKDALVPTKEYQLGVTSIFIKTPETLFELEHKKDLYWHNMATKIQKAMRKYFQKKMDAILMIQRRIREKRGGGNK---DQRLRELG
NadMyo1       668 YTWQGETLDAIKYILADASIPATEYQLGTTSVFIKTPETLFALENMRDKYWHNMAARIQRAWRRFLQRRIDAAIKIQRTIRERKSGNK----YVQLRDYG
```

```
                       810       820       830       840       850       860       870       880       890       900
                ....|....|....|....|....|....|....|....|....|....|....|....|....|....|....|....|....|....|....|....|
CnbMyo1A      764 TKLLGG-RKERRSMSLLGYRAFMGDYLSCNESKSK-GAYIKRQAGIQEKVIFSFHGEALHSKFGRSAQRLKKTFILS-PTAFYIIGQVRVQNAMQYT--A
CdnMyo1A      764 TKLLGG-RKERRSMSLLGYRAFMGDYLSCNESKSK-GAYIKRQAGISEKVIFSFHGEALHSKFGRSAQRLKKTFILS-PTTLYIIGQVKVQNAMQYT--A
CacaMyo1A     765 TKLLGG-RKERRSMSLLGYRAFMGDYLSCNESKSK-GSYIKKKVGIEGRVVFSIHGEALHSKFGRSAMRLKKTFILT-PTTFYIVGQAKVQNSLQYT--V
NabMyo1A      764 TKLLGN-RKERRSMSLLGYRAFMGDYLSCNESKSK-GAYIKRQVGIKEKVIFSIHGEALHSKFGRSAQRMKKTFILT-PTTLYLVGQTMVQNSLQYT--A
NdMyo1A       764 TNLLGG-RKERRSMSLLGYRAFMGDYLSCNESKSK-GAYIKRQAGITEKVIFSFQGEALHSKFGRSAQRLKKTFILS-PTTLYIIGQVKVQNAMQYT--A
CnbMyo1B      766 DKVYGG-KKERRNMSLLGFRGYYGDYLSCNEPKTR-GSFIKRQAGITEHVIFSFHGSSLHSISGKR---LRKTFILT-PTSLWIVGHTKTRNAMKYI--T
CglMyo1A      765 TSLLGN-KKERRSMSLLGYRAFMGDYLSCNESKSN-GSYIKRQAGISEKVVFSFHGEALHSKFGRSAQRLKKTFILS-PTTLYIIGQVRVQNAMQYT--A
Sab_aMyo1B    764 TKILGG-RKERRSMSLLGYRAFMGDYLSCNESKSK-GAYIKRQAGIKERIIFSIHGEALHTKFGRSAQRLKKTFLLS-PTTFYIVGQTLVQNAMTYT--Q
Sc_cMyo1B     764 TKVLGG-RKERRSMSLLGYRAFMGDYLSCNESKSK-GAYIKRQVSIKEKVIFSIHGEALHTKFGRSAQRLKKTFLLT-PTTLYIVGQTLVQNAMTYT--Q
SaaMyo1B      764 TKVLGG-RKERRSMSLLGYRAFMGDYLSCNESKSK-GAYIKRQVGIKETVIFSIHGEALHTKFGRSAQRLKKTFLLT-PTTLYIVGEALVQNTMTYT--Q
SakMyo1B      764 TKILGG-RKERRSMSLLGYRAFMGDYLSCNESKSK-GAYIKRQVGIKEKIIFSIHGEALHTKFGRSAQRLKKTLLLT-PTTFYV---------------
SmiMyo1B      764 TKVLAG-RKERRSMSLLGYRAFMGDYLSCNESKSK-GAYIKRQVGIKEKVIFSIHGEALHTKFGRSAQRLKKTFLLT-STTFYIVSQTLIQNAMTYT--Q
Sap_aMyo1B    764 TKVLGG-RKERRSMSLLGYRAFMGDYLSCNESKSK-GAYIKRQVGIKEMIIFSIHGEALHTKFGRSAQRLKKTFLLT-PTTLYIVGQTLVQNAMTYT--Q
NacMyo1       764 TKLLGG-RKERRSMSLLGYRAFMGDYLSCNESKSK-GSYIKRQAGIKERVVFSIHGEMLHSKFGRSAQRLRKTFILT-PTTFYIVGQTMVQNSMKYM--Q
KaaMyo1       764 TKLLGG-NKERRSMSLLGYRAFMGDYLSCNESKSR-GSYIKRQVGIKEVVVFSIQGESLHSKFGRSAQRLKKVFILT-PTTFYIVGQTMVQNAMKYV--Q
VpMyo1        764 SKILAA-RKERRTMSLLGFRAFMGDYLLCNERKSR-GAYIKKQAGISDKVVFSIKGEQLQSKFGRSSVRVKKVLILT-HSHLYIIGQSMVQNSVQYT--T
TtpMyo1A      764 TELLGG-RKERRSMSLIGYRAFMGDYLLCNDSKAR-GAYIKRQAGIKDSVIFSIKGHCLQSKFGRSSVRVKKVFILT-AKTFYVIAQTNVQNAMQYS--L
TtpMyo1B      764 TELLGG-RKERRSMSLIGYRAFMGDYLLCNDSKAR-GAYIKRQAGIKDSVIFSIKGHCLQSKFGRSSVRVKKVFILT-AKTFYVIAQTNVQNAMQYS--L
TtbMyo1A      774 NSLLYG-QKKRRRFSMSGYRGYYGDYLSCNDEKSL-GSYITRNAGITDRILFSINGSELHLRNNKPAQKFKRTFVLT-ETTFYIIAHKIVHNAMTYS--L
TtbMyo1B      766 NTLVVN-KKERRSMSLLGSRAFLGDYLSMNEQKSN-GAYIKRQVSIKDKVIFSIHGEALQTKFGRSARRLKKTFILT-PTAFYVVGQALVQNSMTYQ--T
ErgMyo1       763 NKLLGG-RKERRAMSLLGYRAFLGDYLSCNELKSK-GAFVKRKAGINDHVVFSINGQALHSKFGRSAQRLPKTFILT-PSHFYIIGKTLVNNQLTYA--V
ErcMyo1       764 NNLLGG-RKERRAMSLLGYRAFLGDYLSCNELKSK-GAFIKRKAGIDEHVIFSIDGESLHSKFGRSAQRLSKTIILT-PTNLYIIGKTVVENQLSYS--V
HsvMyo1       763 TDKVLAYRKERRSMSLLGYRAFMGDYLSCNEPKSK-GSFIKKQAGISEFVVFSMKGEALLSKFGRSAQRLPKTIILT-KTVLYIVGQTVLNNQISYT--V
KlMyo1        762 HRLLGG-RKERRAMSLLGYRAFMGDYLSCNEMKSK-GAFIKRQAGISEIVVFSIKGEALHSKFGRSAVRLPITIILT-PTTLYIVGQTVAQNQVSYT--V
KmmMyo1       762 NRLLGN-RKERRAMSLLGYRAFMGDYLSCNEMKSK-GAYIKRQAGIKEVVVFSIHGEALHSKFGRSAVRLPKTIILT-PTTLYIIGQAVAQNQVSYT--V
KlwMyo1       762 NTLLGG-RKERRAMSLLGYRAFMGDYLSCNESKSK-GAFVKRQAGISELVVFSINGESLHSKFGRSAVRLAKTIILT-PTTLYVIGQTVAQNQVAYT--I
KaMyo1        763 SSLLSG-RKQRRTMSLLGYRSFMGDYLSCNELKSK-GAYIKRQAGISEKVMFSMHGEILHSKFGRSAVRLPRTLILT-PTSLYIVAQTVVQNQASYT--V
KnMyo1        764 SSLLGG-QKERRTMSLLGYRAFMGDYLSCNESKTK-GSYIKRQAGIKERVVFSFTGEALHSKFGRSAQRLPKTFIVT-PTKFYIVAQAMLQNSMNYA--A
LwMyo1        764 SKLLAG-RKERRSMSLLGCRAFMGDYLSCNESKSK-GAYIKRQAGVTDKVIFSINGESLHSKFGRSALRLKKTFILT-SSHLYIIGQNAVQNQITYA--V
LatMyo1       764 TKLLAG-RKERRSMSLLGYRAFMGDYLSCNEPKAK-GAYIKRQAGINEKVIFSINGESLHSKFGRSALRLRKAFILT-PSHFYVIGQNAVQNQITYT--V
Lak_aMyo1     768 TKLLGG-RKERRSMSLLGYRAFMGDYLSCNESKSK-GAYIKRQAGISEKAIFSIHGEALHSKFGRSAQRLKKTFILT-SKALYIIGQTMVNNQVSYT--V
Sab_aMyo1A    764 TKLLGG-KKERRSMSLLGYRAFMGDYLSCNESKTK-GSYIRRQAGIKEKAVFSMQGECLHSKFGRSAQRLKTVFILT-KKALYIVGQTRVQNAMKYT--Q
Sc_cMyo1A     763 TKLLAG-KKERRSMSLLGYRAFMGDYLSCNESKTK-GSYIRRQVGIKDKVVFSIKGECLHSKFGRSAQRLKKVFILT-KKTFYIIGQTREQNAMKYT--Q
SaaMyo1A      764 TKLLDG-KKERRSMSLLGYRAFMGDYLSCNESKTK-GIYIRRQAGIKEKAIFSMKGECLHSKFGRSAQRLKKVFILT-KKAFYIIGQTRVQNAMSYT--Q
SakMyo1A      573 -------------------------------------------------------------------------------------------MRYT--Q
SmiMyo1A      764 TKLLGG-KKERRTMSLLGYRAFMGDYLSCNEAKTK-GSYIRRQAGIKEKAVFSMKGECLHSKFGRSAQRLKKVFILT-KKSFYIIGQSRVQNSMKYM--Q
Sap_aMyo1A    764 AKLLGG-KKERRSMSLLGYRAFMGDYLSCNESKTK-GSYIRRQVGIKDKVVFSIKGECLHSKFGRSAQRLKKVFILT-KKTFYIIGQTREQNAMKYT--Q
ZrMyo1        764 HKLLGG-RKERRTMSLLGYRAFMGDYLCTNERKSK-GAYIKRQAGIKERTVFSMYGDMLHTKFGRSAQRLKKTFILT-SRHFYIIGQVMQQNTLRYS--V
TodMyo1       764 TKLLGG-RKERRSMSLLGYRAFMGDYLSCNERKSK-GAFIAKQVGVTERVVFSIHGEALHSKFGRSAQRMRKTFILT-STYFYIIGQVMIQNTLSYN--P
CglMyo1B      766 DKVYGG-KKERRNMSLLGLRGFYGDYLSCNESKTR-GSYIKRQANITERVLFSSHGNSLHAMYGGASQRLRKTFILT-PTSLWIVGHTKARNAMKYI--T
WaMyo1Alpha   764 TKLLGA-RKERRSMSLLGYRAFTGDYLSANERNSR-GAFIKNQVGIKDKIIFSANGEALHSKFGRSSQRVPRTVFLS-STTLYIVAHQVIDRKVSYT--I
WicMyo1       764 TKLLGS-RKERRSMSLLGYRSFMGDYLNSNELNTR-GSFVKNQVGIKEKIIFSSNAESLHSKFGRSSTRVPRTVFLT-SSTVYIVAHQVIDRKVSYT--L
CyjMyo1       756 TRLLGS-RKQRRKFSLLGYRQFTGDYLNANERKSK-GAFIKNQVGIKDKVIFSSGCEVLHSKFGRSAQRLPRHVFVT-SSTVYIVAHQVVDRKATYT--M
YlMyo1        767 HKLLGG-RKERRTYSLIGYRRFQGDYLGCNNGSG-FGDFLMKQIGVTDKVFFSCRGQLQQSRLGRSSVRVPRTFILT-KNNFYVVAQQIHQKQLVVT--N
CdnMyo1B      766 DKVYGG-KKERRNMSLLGFRGFYGDYLSCNENKTR-GAFIKRQAGVTEHVIFSFHGSAIHSITGKR---LRKTFILT-ATDLWIIGHTKTRNAMKYI--T
Kop_bMyo1     769 TSLVIH-KKERRRMSMLGSRQYTGDYLGCNDKSG-FGRFLISQVGINDRIIFSFRGEILHAKFGRSSVRVPRIFLLT-RSTFYMIAEEVIERKISYN---
NdMyo1B       765 ENIYGG-KKERRNMSLLGFRGFYGDYLSCNENKTR-GSFIKRQAGVTEHVIFSLHGSALHAITGKR---LRKTFILT-ATDLWIVGHTKTRNAMKYI--T
KcMyo1        767 TSLVIN-KKERRRFSMNGYRAYMGDYLNCNDKSG-FGKFLLGAANITGFVVFSLRGETLHAKFGRSSTRVPRIFILT-TTTFFMIAEQVVERKLQYV--K
OgpMyo1       771 TSLVVN-KKQRRTLSILGSRAYMGDYLGCNDKTGGFGKFLMQSIGLSDYVIFSSRGQILHSKFGRSSQRLNRIYILT-SSQFYVIAEELVNRKVSYV--V
DebMyo1       772 SKMLGG-RKQRRRLSILGSRSYLGDYLGCNDQTGGFGKFLMDAIGASYKISFSARGQQLHSKFGRSAERLPKIFLITSSGEFVIVGQQIIEGQAKYS--I
PiuMyo1       775 NQIVYG-KKQRRRLSVLGSRQFVGDYIGVNDKTGGFGKFIKNAINLKDKVKFSAKGDQLHSKFGRSSQRLPKIFLITKDDRFIVVGQNVVNNKAQYS--V
NabMyo1B      764 NRLLDN-RKERRTLSLLGYRGFYGDYLSCNEPRSR-GSYIKRQAGIKEKVNFSIQGQSLKSKLGRSAIRIRSTLILT-ATTFYVINKVKTQNTVKYL--V
ZbMyo1        764 TKLLGG-KKERRTMSLLGYRAFVGDYLGTNERKSK-GAYIKRQAGIKEKTLFSMYGEALRTKFGRSALRVKKTFILT-SKYFYIVGQVRQQNTLHYA--V
Ca_bMyo1      780 NGLLQG-RKERRRMSMLGSRAFMGDYLGCNYKSG-YGRFIINQVGINESVIFSSKGEILLSKFGRSSKRLPRIFIVT-KTSIYIIAEVLVEKRLQLQ--K
StaMyo1       778 NGLLQG-RKERRRMSMLGSRAFMGDYLGCNFKSG-YGRFIVSSIGLNEQVVFSGKGEILTAKFGRSSKRSPRIFILT-RSSLYVIAEVLVDKRLVLQ--S
ShpMyo1       776 NGLLQG-RKERRRMSMLGSRAFMGDYLGCNFSSG-YGRFIVNQIGLKEQVIFSAKGEILTAKFGRSSKRMPRIFILT-RSNLYIIAEVLVEKRLQLQ--T
CllMyo1       780 SALLQG-RKERRRMSMLGSRAFMGDYLACNDSTG-FGRFVVSQTGISEPVIFSSKGEILLSKFGRSSKRLNRIFILT-KSYLYVIAEVLVEKRLQLQ--K
MefMyo1       779 TAILQG-RKERRRFSILGSRAYMGDYLGCNDSSG-YGRFVVSQTSISEPVVFSGKGEILLSKFGRSSKRLSRIFVLT-RSSFIVIAEALVENRLQLQ--K
CnmMyo1       778 NGLLQG-RKERRRMSMLGSRAFMGDYLGCNYKSG-YGRFVVSQAGINDQVIFSSKGEILLSKFGRSSKRLPRVFVLT-KTTLFVVAETLIEKRLQLT--K
DhhMyo1       779 NGLLQG-RKERRRMSMLGSRAFMGDYLGCNDSSG-FGRFIMNQAGVSERVIFSAKGDILLSKFGRSSKRLPRIFILT-KNSLYVIAQTLVQNRLQVQ--K
DehMyo1       779 NGLLHG-RKERRRMSMLGSRAFMGDYLGCKYSSG-FGRFIMSQAGISDSVIFSAKGDILLSKFGRSSKRLSRIFILT-KNSLYVIAQTLVQNRLQVQ--K
CatMyo1       779 NGLLQG-RKERRRFSMLGSRAFMGDYLGCNDSTG-YGKYVVNQAGISEHVIFSSKGEILIAKFGRSSKRLARIFIMT-KNSFYVVAETLVEKKLQLL--K
LoeMyo1       785 NSLLQG-RKERRRFSMLGSRAYMGDYLACNDKTG-FGRFVVNQVGIKEPVVFSAKGVILLSKFGRSSKRFPRIFVLT-KSNLYIIAEVLVEKRLQLQ--R
MrgMyo1       777 NGLLQG-RKERRRMSMLGSRAFMGDYLGCNYSSG-FGRFVLNQVGLNEHVVFSGKGEILLSKFGRSSKRLPRIFVLG-RSSLYIIAENLVERRLQLS--K
ShsMyo1       773 NGLVQG-RKERRRMSMLGSRAFMGDYLGCSDTSG-YGRFIVTQTGINEKVVFSGRGEILLSKFGRSSKRLPRIFVVT-RTTIFIIAEVLIEKRLQLQ--S
CameMyo1      779 NSLLQG-RKERRRLSMLGSRAFMGDYMNCNDKSG-YGRYVVTQAGINDYVVFSGQGDILLSKFGRSSKRLPRYFILS-KTNLYVVAAVLVNKRLQLQ--K
MifMyo1Alpha  778 NSLLKG-QKERRRLSMLGSRAFMGDYLGCNDSSG-YGRYILNQIGIQQKIIFSGNGEILVSKFGRSSKRLPRIFLLD-KSNLYVIAQVLVDKRLQLQ--K
CadMyo1       781 NDLLQG-RKERRRMSMLGSRAFMGDYLGCNYKSGGYGRFIINQVGINESVIFSSKGEILLSKFGRSSKRLPRIFIVT-KTSIYIIAEVLMEKRLQLQ--K
CaoMyo1       779 NTLLQG-RKERRRLSMLGSRAFMGDYLGCNDKSG-YGRFVINQAGIKDHVVFSGKGEILLSKFGRSSKRLPRIFILS-KTSLYVVAEALVEKRLQLQ--K
CapMyo1       779 NSLLQG-RKERRRLSMLGSRAFMGDYLGCNDKSG-YGRFVVNQAGIKDHVVFSGKGEILLSKFGRSSKRLPRIFILS-KTSLYVVAEALVEKRLQLQ--K
Ct_aMyo1      777 NGLLQG-RKERRRFSMLGSRAYMGDYLGCNYKSG-YGRFVVSQAGINDHVVFSCKGEILLSKFGRSSKRLPRVFILT-KSTLYIVAEMLVEKRLQLS--K
PtaMyo1       770 NTLVYN-KKERRSYSLLGSRAFLGDYLNCNGGGS-FGSFIKKNAGITDRVIFSSRCESLYHKFGRSSMRIPRILILA-KSTLYILEEAYNQQLKKITINK
CacaMyo1B     768 NTLLAG-KKERRTFSLLGNRSFMGDYLSCNEAKT-KGEYIKKLTGSNEKVIFSIHGSLLHYKVGRAAMKIKSTFILT-RSTLFIMNRLKQTPQNRTGYET
NadMyo1       764 TKILGD-RKERRSMSLLGYRAFMGDYLACNQSKS-KGSYIKRQAGIKEKVIFSIHGEMLHSKFGRSAQRLKKTFILT-PTTFYIVGQTMIQNAMKYT--Q
```

```
                                  910       920       930       940       950       960       970       980       990      1000
                         ....|....|....|....|....|....|....|....|....|....|....|....|....|....|....|....|....|....|....|....|
CnbMyo1A         859     EYKININSIIQLSLTNLQDDWVGVVLANSSL--PDPLINLSFKTELITHLKMLNDKIQIKVGPTLDYQKKPSKMHSVKCQISDTAPKYS--DLYKSSTIF
CdnMyo1A         859     EYKINVNAIIQLSLTNLQDDWVGVVLANSSL--PDPLINLSFKTELITHLKKLNDKIQIKVGPTLDYQKKPSKMHSVKCQISDSAPKYG--DMYKSSTIF
CacaMyo1A        860     DYSISVESIKQVSLTSMQDDWIGIVLSNSTL--PDPFINTYFKTELITHLKQLNPKIQVQVGPTIQYQKKPRKFHAVKTQISDTAPKYG--DVYKSSTIY
NabMyo1A         859     DYKINVNSIQQVSLTNLQDDWVGIVLINSTL--PDPFINTYFKTELITHLKKVNPKITIKVGPTIEYQKKPRKIHSVKAQVSDSAPKYG--DIYKSSTIS
NdMyo1A          859     EYKINVNSIIQLSLTNLQDDWVGVVLANSTL--PDPLINLSFKTELITQLKKLNDRIQVKIGPTLDYQKKPSKMHSVKCQISDSAPKYG--DMYKSSTIF
CnbMyo1B         858     DYRIDVHNIRSISVTNLQDDWLAINLASSPK--PDPLINLKFKTELITHIKQLNPKITICVGNVIEYQKMPKKMFAVKAQINESAPKHG--DLYRSGTIL
CglMyo1A         860     DYKINVNSILQLNMTNLQDDWVGIVLANSSM--PDPLINLSFKTELITHLKTLNSKIQVKVGPTLEYQKKPGKMHSVKCQVSDTAPKYG--DVYKSSTIF
Sab_aMyo1B       859     DYKIDVRSIQAVGLTNLQDGWIAINMTNSTQ--PDPLINTYFKTELITHLKKLNDKIQVKIGPTIEYQKKPGKLHLVKCQVSESAPKYS--DIYKSSTIS
Sc_cMyo1B        859     DYKIDVRNIQAVSLTNLQDDWVAIKLASSGQ--PDPLINTYFKTELITHLKRLNDKIQIKIGSAIEYQKKPGKLHSVKCQINESAPKYG--DIYKSSTIS
SaaMyo1B         859     DYKIDVQSIQSVSLTNLQDGWIAVNLASPTQ--PDPLINTYFKTELITQLKKLNDRIQVKIGSTIQYQKKPGKLHSVKCQVSESAPKYG--DTYKSSTIS
SakMyo1B         844     --------------------------SNSGQ--PDPFINTYFKTELVTHLKKLNDKIQVKIGPTIEYQKKPGKLHAVKCQLSESAPKYS--DIYKSSTIS
SmiMyo1B         859     DYKIDVRNIQAVSLTNLQDGWLAINLTNSSQ--PDPLINTYFKTELITHLKKLSDGLQIKIGSTIEYQKKPGKLHSVKSQVNESAPKYG--DIYKSSTIS
Sap_aMyo1B       859     DYKIDVRNIQAVSLTNLQDGWVGINLTGSGQ--PDPLINTYFKTELITHLKRFNDKIQIKIGSTIEYQKRPGKLHSVKCQINESSPKYG--DIYKSSTIS
NacMyo1          859     DYSINLKSIREVSLTSLQDDWIGIVMSGSSL--PDPFINTSFKTELITQMKKLNGGIQVKVGPTIQYQKKPGKLHSVKSSISDTAPKYS--DLYKSSTIF
KaaMyo1          859     EYAINVNSIRSLSLTNLQDDWIGIVLSNSAQ--PDPLINTYFKTELITHLKKLNNNIQIKIGATLDYQKKPGKLHPVKSSISESAPKYG--DIYKSSTIF
VpMyo1           859     EYKIDVNKIAGVSMTNLQDDWVAINLSNSTQ--PDPLLHTFFKTELVTQLKKLNGRIQVKIGTVIEYQKKPGKMHKVKSQVSEATPKYN--DNYKSGTIL
TtpMyo1A         859     EYNIPVDHIVSVGLTNLQDDWMAINLAKSNQ--PDLLINTFFKTELITHLKKLNSKIQIKIGPTIEYQKKPGKIRRVKCEITEAAPKYG--DNYKSRKIY
TtpMyo1B         859     EYNIPVDHIVSVGLTNLQDDWMAINLAKSNQ--PDLLINTFFKTELITHLKKLNSKIQIKIGPTIEYQKKPGKIRRVKCEITEAAPKYG--DNYKSKKIY
TtbMyo1A         869     EYEVPLEYIESISITNLRDDWMAINLIEFDQ--PDPFIRTFFKTELIGRILTQNSNIQLRIGPTIEYQRNVGEITSVQAVESEDAPKMK--DLYKDDTIF
TtbMyo1B         861     DFRIPISSIHSISMSKLQDDWLAININDPSQ--PDPFINTYFKTELITHLKQLNPKINIKIGPTIEYCKKPKKIHVVKAQYSESAPKYS--DTYKSSTIS
ErgMyo1          858     DYKIDVRQIVYVSLTNLQDDWVGIFVSNSTQ--PDPLINTYFKTELITHLKKLNNRIEIKIGPTIEYQKKPGKIHAVKSQINENAPPLG--DIYKSSTIM
ErcMyo1          859     DYKIDVGRIQFVSLTNLQDDWVGIFVNGATH--PDPLINTYFKTELIAHLKKLNNRIDIRIGPTIEYQKKPGKIHIVKSHVNETAPKYG--DIYKSSTIS
HsvMyo1          859     DHKIPITQIANVSLTNLQDDWIGVYVNNAVQ--PDPLINCIFKTELITHMKKLNPRLQVKIGPNIEYQKKPGKVHLVKSKVDASAPLNG--DFYKSSTIS
KlMyo1           857     DRKVNISHIKHVSLTNLADDWVGIYVQGENL--PDPFINTIFKTELITHLKQLNRAIEVKIGPTIEYQKKPGKLHTVKCQISESAPKYS--DIYKSSTIS
KmmMyo1          857     DRKVNVAHIKYVSLTSLADDWVGIYVQGENL--PDPFINTVFKTELITHLKQLNRALEIKVGPTITYQKKPGKIHTVKCQVSQAAPKYG--DIYKSSTIS
KlwMyo1          857     DRKVKVSSIKYVSLSNLADDWVGIYVTGDNL--PDPFINTVFKTELITHLKKLNTSITVKVGP--------------KSKISESAPKYG--DIYKSSTIS
KaMyo1           858     DRKVDVSRIQNVSLTNLADDWVGIYVNGDNL--PDPFVNTIFKTELITHLKKLNKSIVVKVGPTIEYQKKPGKVHIVKCQISESAPKYS--DMYKSSTIS
KnMyo1           859     EYVIDIKTIKQLNMSNLQDGWIGVVLANSQQ--PDPFICTDFKTELVTHLKKQNNNIHIKCGTTIEYQKKPGKRHLVRSTVSETAPKYG--DIYKSSTIY
LwMyo1           859     DYKIDVRQIKFVSLTNLQDDWIGVFVANATL--PDPLINTCFKTELITHLKKLNSGIEVKVGPTLEYQKKPGKIHLVKCQISESAPKYG--DIYKSSTIS
LatMyo1          859     DYKIDVKQIKYVSLTNLQDDWVGIFVANSTL--PDPLINTYFKTELITHLKSLNKGIEVKIGPTLEYQKKPGKIHVVKCQISESAPKYG--DIYKSSTIS
Lak_aMyo1        863     DYNIDVNIIQSISLTNLQDDWIGINIPQSTQ--PDPFVNTYFKTELITHLKKLNNRIDVKVGPTIGYQKKPGKLHSVKSQVSEAAPKYG--DIYKSSTIS
Sab_aMyo1A       859     DYGIDINKIRQVNLTNLQDDWIGIILTNSTQ--PDPFINTPFKTELVTHMKKLNEKIIVNVGPTIEYHKQPSKSHIVRSKISDSAPKYS--DIYKSSTIF
Sc_cMyo1A        858     DYKIDVGKIKQVSLTNLQDDWMGVILVNSTQ--SDPLINTPFKTELMTRLKKLNEKIMIKVGPTIEYHKQPNKLHTVRSKISDSAPKYG--DIYKSSTIY
SaaMyo1A         859     DYNIDLDKIKQVSLTNLQDDWMGIVLVNSAQ--PDPFINTPFKTELVTHMKKLNKKIIVDIGPAIKYHKQPNKLHTVRPKISDSAPKYG--DIYKSSTIY
SakMyo1A         579     DYKIDIGEIKQVNVTNLQDDWMGVILANSIQ--PDPLINTPFKTELVTRMKKLNEKIKIRVGPTIEYHKQPNKLHTVRSKISDSAPKYG--DIYKSSTIY
SmiMyo1A         859     DYKIDLNKIKQVSLTNLQDDWMGIVLINSAQ--SDPLINTLFKTELVTRMKKLNEKIITNIGPTIEYHKQPNKLHTVRSKISDSAPKYG--DIYRSSTIY
Sap_aMyo1A       859     DYKIDLGKIKQVCLTNLQDDWMGVILLNSAQ--PDPLINTPFKTELVTRMKKLNDKIIIKIGPTIEYHKQPNKLHAVRSKISDSAPKYG--DIYKSSTIY
ZrMyo1           859     DYQIDISNVRSVSLTNLQDNWVAINLLNATE--PDPLINCIFKTELITQMKRLNSNIHIKIGQTIEYRKKPKKIHIVRCQVDDSAPLRG--DIYKSSTVL
TodMyo1          859     EYKINVKNISSVSLTNLQDDWVGINVLNATQ--PDPLINTYFKTELITQLKKLNNKLQIKIGPSIEYQKKPNKLHAVKCQISETAPKRG--DLYKSSTIF
CglMyo1B         861     DYRIDLGKIRSISVTNLQDDWMAVNLMDSPK--PDPLINLPFKTELITRLTQLNPRIHVKVSSTIEYLRGPKKLFVVKSQYSDSAPKYH--DLYRNGTIL
WaMyo1Alpha      859     ENAIPLGNINYLGLSNLQDDWIAISLKNTPT--PDPFINLAFKTELVTQLKKLNNAIRIEIDTTIKYQKKPGKYHTVKTQVSGTAPKNG--DFYKSGTIQ
WicMyo1          859     ESSIPISNINYLGLSNLQDDWVAISLKNGSS-IPDPFINLSFKTEFITHLKKLNNSIKIDIDTTIKYQKKPGKFHTVKTQISSTAPPYS--DLYKSGTIQ
CyjMyo1          851     EMSVPINNINYLGLSNLQDDWVAISQKTQAV---DPFICLWSKTELVTQLKKLNSAISIQIDSTIKYQKKPGKYHIVKTAVNPNAPLHG--DLYKSGTIS
YlMyo1           862     EYTIPVRNITHMSMSNLRDDWFCLNQASSPY--GDQLMWCVFKTELAVQLRVVKPGVDIRIGPQIDYFKKQGKKASVKFQKTTTLQTKF--DMYKSGSVQ
CdnMyo1B         858     DYRIDVHIIRSISVTNLQDDWLAINLSNTPK--PDPLINLKFKTELITHIKQLNSKLSIRVGNGIEYQKVPKKMFVVKAQISDTAPKYS--DLYRGGVIL
Kop_bMyo1        863     VDSISVNSISYVGMSNLADDWLAISLIGGTR--PDPFINLKFKTELVTHLKNINRGLSIQIGPTIQYYKKPGKIHTIKTQQSTAVPLNG--DVYKSGTIQ
NdMyo1B          857     DYRIDLHTIRSISVTNLQDDWMAINIANSPK--PDPLINLKFKTELITHMKQLNPKITVRVEKGIEYQKVHKKMFVVKAQVSDAAPKYS--DLYRNGTIL
KcMyo1           862     ELAIPVNQIAGVSVSNLQDDWIAFNLTGDTQ--PDPFFNVCLKTELITHLKNMNRSLNIKIGPTVVYNKKPRKPHTIKYQINEAAPELS--DLYKSGTVQ
OgpMyo1          867     ETKIPVSAIHKVSLSQYQDDWVALNLNSTTQ--PDLFINTIFKTELVTHLKKLNRSLGIDIGPTITYNKKPGKPHVVKFQADSTSPKNG--DSYKSGTVK
DebMyo1          869     EEKISANQIPKISMSSFADDWLAVNLQSSTR--PDLFINTMFKTELVTRLKKANSALQIEIGPIVSYARKPGKMRTIKFRSSSEAPAEG--DIYKSSTVT
PiuMyo1          872     EFNKPITSIPQVSVSNLADDWLAINLNSKTE--PDLFINCLFKTEILGRFKKANPAITINIGSKIVYCKKPGKKHTVKFQLATSGNT----DAYKSSTVS
NabMyo1B         859     EHKVDVKKIKQVSLTNLQDNWVALNLMDASQ--IDPFVNSYFKTELVTHLKKLNSKIQIKIGPAIEYCKSPRKMQVVKAKISDTAPKFS--DLYKSGTIF
ZbMyo1           859     EYKIDVNSIRSVSLTNLQDNWVAINLVNSQQ--PDPFINTVFKTELVTRLKRLNNNIQIKIGPTIEYHKKPRKVHVVRCDINDTAPLRG--DTYKSSRIY
Ca_bMyo1         875     EFTIPISGINYLGLSTFQDNWVAISLHSPTPTTPDVFINLDFKTELVAQLKKLNPGITIKIGPTIEYQKKPGKFHTVKFIIGAGPEIPNNGDHYKSGTVS
StaMyo1          873     ELTIPLNSINYVGLSTLQDNWIAISLHSSTPNLHDVLINLDFKTELITHMKKINPGLTIKIGPTVEYQKKPGKYHSVKFVIGSGVEVPINSDHYKSGTVT
ShpMyo1          871     ELTIPLGGINYVALSTLQDNWVAVSLHSFTPTTHDVLINLDFKTELITHMKKISPGLTIKVGPTVEYQKKAGKYHSVKFTIGSGAEVPVNSDHYKSGTVT
CllMyo1          875     EFSIPISGIKYVALSTLQDNWVAVALHTPTSTTPDVFINLDFKTELIAQIKRVNRGLTIKIGPTVEYCKKPGKVHVVKFVHGTAPRFS---DVYKSGTVT
MefMyo1          874     EFTIPISGINYLGLSTFQDNWLAISLHSPTPSTPDIFINLDFKTELVTQIKKINRGVTVKIDATVQYQKKPGKFHVVKFQHGSAPMNS---DTYKSGTVT
CnmMyo1          873     EFSIPVSGINYVGLSTLQDNWVAVSLHSPTPTTPDIFINLDFKTELVAQLKKLNPGLTIKIGPTVEYQKKAGKYHTVKFISGSGPEVPVNGDHYKSGTVT
DhhMyo1          874     EFTIPVSGINYVGLSIYQDNWVAVSMHSPTPTTPDIFINLDFKTELITHLKKCNPGLSIKIGQTVEYQKKPGKFHTVKFVVGNTAPVTG--DTYKSGTVT
DehMyo1          874     EFTIPVSGINYVGLSIYQDNWVAVSLHSPTPTTPDIFINLDFKTELVTHLKKCNPGLNIKIGQTIEYQKKPGKFHTIKFIVGNTAPVNG--DSYKSGTVT
CatMyo1          874     EFTLPVGGLNYVGLSCYQDNWLALSLHSPTPSNPDLFINLDFKTELVAHLKKANPGLTIKIGPTIEYQKKPGKFHVVKVVVG-AAPKNG--DVYKSGTIT
LoeMyo1          880     EFALPVHSIKSVGLSQLQDNWVAVCLSSPTTTTPDVFINLDFKTELVAQLKKLNSGLSILIGPTVQYQKKPGKYQTVKFAIGSGPHIPPLIDSYKSGTVT
MrgMyo1          872     EFVIPINSINYVGLSTFQDNWLAVSLHSPTPTTPDVLINLDFKTELVTHLKKLNPGLTIKIGPTIEYQKKPGKFHTVKFVRSDVSTIPIHGDVYKSGTVS
ShsMyo1          868     EFKIPISGLNYVALSHLQDNWVALSLHSPTPSTPDVFISLDFKTEFVTHLKKINPGLTINIGTTVQYQKKPGKFHTVKFISGNSSDVPEYGDVYKSGTVT
CameMyo1         874     EFAIPVNSIKNVGLSTNQDNWVAVCLHTPTTTVPDIFINLDLKTELITHLKKLNPGLSIIIGPTIQYQKKPGKYHTVKSVVGSGPQIPPRSDVYKSGTIT
MifMyo1Alpha     873     EFVVPVSSINYVGLSTYQDNWVAVSLHSPTPTTPDLFINLDLKSELVTHLKKLNPGLTIKIGPTVSYQKKPGKYHTIKFEISNAANVPRNSDLYKSGTVT
CadMyo1          877     EFIIPINNINYLGLSTFQDNWIAISLHSPTPIIPDVFINLDFKTELMAQLKKLNPGIIIKIGPTIEYQKKPGKFHTVKFIIGSGPEIPINGDHYKSGTVS
CaoMyo1          874     EFVIPINSIKSVGLSTNQDNWVAVCLHTPTAMVPDIFINLDLKTEFITHLKKLNPGLSIVIGPTIQYQKKPGKYHTVKSVAGSGPQIPPRSDLYKSGTIT
CapMyo1          874     EFVIPINSIKSVGLSTNQDNWVAVCLHTPTTMVPDIFINLDLKTEFITHLKKLNPGLSIIIGPTIQYQKKPGKYHTVKSVVGSGPQISPRSDVYKSGTIT
Ct_aMyo1         872     EFSIPVSGINYVGLSNLQDNWIGVSLHSPTPTTPDVFINTDFKTELVAQLKKLNAGLTIKIAPTVEYQKKPGKYHSVKFIHSSGPEVPVNGDHYKSGTVS
PtaMyo1          867     EYAIPVSSIQSLNLSSFQDDWLAIIINSQTQ--ADPFINLMFKTELVTHLKNINQSLSIVIGPTVQYHKKAGNSKLHVIKYQYGDLAPVSGDIYKSGTIT
CacaMyo1B        865     GYKIAVDSIRQISMSNLQDNWIALNLGNSAK--SDAFINTPFKTELITHLKELNGNIKMNIGTPVQFIKLGGKNEAINFKISSSAPKYG--DIYKSGTVY
NadMyo1          859     DYSINVNSIKQFSLSTLQDDWVGVVLSNSTL--PDPFINTYFKTELITQIKKLNSKVQINVGPMIQYQKKPGKLHPVRATISDTAPKYG--DLYKSSTIY
```

```
                       1010      1020      1030      1040      1050      1060      1070      1080      1090      1100
                   ....|....|....|....|....|....|....|....|....|....|....|....|....|....|....|....|....|....|....|....|
CnbMyo1A      955  VRRGNPANSKSKKKPQ----------------------------------------------------KKSTLGSATANTATTHRTLAPARTSTTTTKKP
CdnMyo1A      955  VRRGNPANSKSKKKPL----------------------------------------------------KKSTFSSASVPASTTHRTLAPARTNTTASKKP
CacaMyo1A     956  VRRGHPGNSKSNKKPR----------------------------------------------------SRTSFVATTNG-VGHIGSTQTTPVANLSSRKP
NabMyo1A      955  VRRGNPASSKSKKKPR----------------------------------------------------GESSVNITQRRTTAPIQNNVPAQTNHVVQPTP
NdMyo1A       955  VRRGNPANSKSKKKPK----------------------------------------------------GKSTLGSASTTTFKTPKTLAPARTNTTKARKP
CnbMyo1B      954  VRRGNPPDSTASNRPD----------------------------------------------------FASGNLVEIDMKVKKSKTKVATKRTPQALPGA
CglMyo1A      956  VRRGNPANSKSKKKPR----------------------------------------------------------KKSSGMSAPTTQSSKTLAPPRMSSNN
Sab_aMyo1B    955  VRRGNPPNSQVRKKPR---------------------------------------------------------------GKSSISSAYRASPSQSTKRLA
Sc_cMyo1B     955  VRRGNPPNSQVHKKPR---------------------------------------------------------------KKSSISSGYHASSSQATRRPV
SaaMyo1B      955  VRRGNPPNSQVHQKPR---------------------------------------------------------------KKSTISSDYRATPSQTTRRPP
SakMyo1B      915  VRRGNPPNSQVHKKPK---------------------------------------------------------------KNSTLSSGYGANSSRTTRRPV
SmiMyo1B      955  VRRGNPPNSQVHKKPR---------------------------------------------------------------NKSSISSSYRASPSQTTKIPV
Sap_aMyo1B    955  VRRGNPPNSLVHKKTR---------------------------------------------------------------KKSSISSGHRASSSQATRKPV
NacMyo1       955  VRRGHPANSKSKKKPR--------------------------------------------------------------NKGGSSSASYSTSYTHNSRPKS
KaaMyo1       955  VRRGHPGNSKQKKKPR-----------------------------------------------------NKPSSSNYSYTSSATTTTSTTTTMNRVPVAS
VpMyo1        955  VRRGHPANSPQKKKPK----------------------------------------------------------------KGKGHSKHHSTSTSAPRSSV
TtpMyo1A      955  VRRGNPANSKQKKKPR-----------------------------------------------------------------GKKSSGIQQSVPAAARPSH
TtpMyo1B      955  VRRGNPANSKQKKKPR-----------------------------------------------------------------GKKSSGIQQSVPAAARPSH
TtbMyo1A      965  VLPGLPVESVPYEQPIRIEDGGIPCEIATWMAQHADEFDKPKPKPRPRQATPRPAAAEPATSSSRSMAASAAQSAYKTPSAQPSAPKPRAQPKPTPTPRK
TtbMyo1B      957  VRQGNPPNSKQHEKPDKKRS-------------------TITPRHNQRPQTHQPQYQKPTVSRNN------AQPQINNNSSIP-APRPKAVKKAAP----
ErgMyo1       954  VRRGRPGNCSQRKKPLSTRLPDTYTTRETGYKNAGHPTNIRQVQEPTHISHSQQHTSSNLGFVYSLNPSAVNHPRPVPSGSVGTTSTPPKQAASAAQAAY
ErcMyo1       955  VRRGRPGNSVQHKKPTG------LSSYASDFPSALNGN--RSFATANSRPVPPSHTNTQIPFEAQHHKKAPEPLPNVNRKPVQHPSMPISNHAANAAKAV
HsvMyo1       955  VRKGNPPNSRSKKRPLKKASS--SVGGSTTVRTTVQKTTVTTSKRASQPVSSQLNGTNKPAAPVPARRSKNPAPPPPNSAQR---------NGTNASAMA
KlMyo1        953  VRQGRPANSRQAPKPEKKSTLLSDGPSYNSNQSKGYGQQQHAQPSYGQQQQQQQRYAPQSHATPQTTQKKRAPPPPGQQN------------------FA
KmmMyo1       953  VRQGLPANSKQHPKPKKKSSLLDGSDGYAPVSTSQRQSRPQPPQAAQQSQPQPQPQQRQNRPVVPQATQKKRAPPPPGANNVSSSNTSSGAG-MDPKNLA
KlwMyo1       939  VRKGLPPNSKQKPRPKRMSTLSGDTGSNNRYSNSRQSQPQPQSKPRYQQPVATQQQY-----IPQAQKKKANPPPPPSANQGSQNTAQK---------MA
KaMyo1        954  VRQGHVGSSKQHPKPKKKSSLLSE--SYDSTSRVQYQ---------------KPVAQTFTNSRPAVSAKKAAPPAPPVAGGRNPSQPQADN-------LA
KnMyo1        955  VRKGHSPGSKQHKKPKGKPGVYSAGPVRSSNSRKATL---------------DPSAS--GASAPVAHQSRPAAPVPRTTSKKPAPPPPGAQGQS----LA
LwMyo1        955  VRRGNPPTSKSHPKPKKQSSGGDYLPSSSTNQYVQAAPSSRNPGKPTAMKPSVVKQAPAKPPAPKTRQKKAAPPPPAKQQVQQTMNAKVDVS------VA
LatMyo1       955  VRKGHPATSKSHPKPKKKSTYQDYAPQHRTSTSIGQP--SIPTATRSTVVPTTGKQAPAKPPAPKIRQKKPAPPPPAKKQSQPAPSSNVAN-------IA
Lak_aMyo1     959  VRRGHPASSRSNPKPKRKSSAGGYATSGGSSYTTTT---VTNTAMGSHQQTAQRVEPQRPVVPQHRTQKKPVPPPPVKRQQPQPQPQLQTQQKPSAVAMA
Sab_aMyo1A    955  VRRGHPPNSKSNKKPK------------------NPGGLKGTPMKSKKSSHTHTHKHKHSHRPHGKAQKKQPLPAQKLNNPLS---------------LA
Sc_cMyo1A     954  VRRGHPANSKSNKKPK------------------NPGGLSGKPIKSKKSKHKSTHKHTHSHRSHRDAAKKQPLPSQKPVNPLS---------------LR
SaaMyo1A      955  VRRGHPANSKSNKKPN------------------NPGGLSGKPIKSKKKKHKHTHKHTHSNRTHVATPKKQPLPTQKSNNPLS---------------LA
SakMyo1A      675  VRRGHPANSKSNKKPK------------------NPGGLNGRPIKSKKSKHKHTRKHTHSHRAHGVTPKKQPLPPQRLNNPLS---------------LA
SmiMyo1A      955  VRRGHPANSKSNKKPK------------------NPGGLSGKPIKSKKSKHKHTHKHAHSHKAHRNTTKKQPLPSQKSTTPLS---------------LA
Sap_aMyo1A    955  VRRGHPANSKSNKKPK------------------NPGGLSGKPIKSKKSKHKSTHKHTHSHRTHRVTAKKQPLPSQKPTNQLS---------------LA
ZrMyo1        955  VRRGNPANSVSNKKPK-----------------------NVGGFTRTTIKKRRSVKKPVPPPPATRTAKKAAPPPPKQLNPAS---------------LA
TodMyo1       955  VRRGHPANSHSHEKPR------------------------KGAYSGSTSTSSNGAQRTAPSAASTRTRKPVPVPAQHRP--------------------A
CglMyo1B      957  VRHGNPPDSTAENRPAFNN----------------------------EDMYGNLMEAKHKTMKKKVGTKRTPQALPTSS-------------------LA
WaMyo1Alpha   955  VQQGHPASSKSKPKPKKQS-------SGATNLDFTP-------------RNTGYTQPAR-------TVRKVPPPAPVQQQQQQPSSYTASAAPTTSNIPQ
WicMyo1       956  VNHGNPATSKSHEKPKRQS-------SGVANLDFTPR------------KQASYVSSGQPK-----STRKVPPPAPGSVHVPPQQQNPQPVFNNNN-NIN
CyjMyo1       946  VRAGAPASSTSHPRPKNKS--------SIGSSSYTPR------STYKPAATPAVSRPAAATTTLP-TARKAPPPAPVMRQQQPTPQPAHTQASAAS---T
YlMyo1        958  VPPGAPPNSVSKETPRGRAKGSRGGAPSRPAARGNAAHTPTPGAAALGYQQHQPGSAQPASPRARKAPPPAPNSRGNGHAESAQPQAYNNLQPHYQSLVN
CdnMyo1B      954  VRRGNPPDSTASNRPDFDSGNLVEIDMKVKKSKTKVATKRTPQALPGASLAAVAAQAAYHPNGYRPPARTQSTKKESTFTPKSPTKTVRKSSTSTTNTTK
Kop_bMyo1     959  VAPGLSASSVSKERPKGKNTGTAVYR-YRTGVEVSNSYSQRSNNYGAAANSGFANPHAQSVPVAAVQPTQRKKVPPPPPQQQKPRTSNIPGAVPVPQVQR
NdMyo1B       953  VRRGNPPDSTSSNRPNFDSGNLVEIDVKVKKNKTKVATKRTPQALPGASLAAVAAQAAYHPNGYKPPARTHPTKKETTHTSHSVVKTPKKSSSSATKKDS
KcMyo1        958  VKRGLPASSTSKPRPKGIDRPYSAARSSTATYSAPHTSAAPVSRAATSSRPAPPPAVSRKPAPQPHASSAAPSASAQAVSAASAAYSHTQATPQRKINRK
OgpMyo1       963  VAAGLPASSTSRQKPKG-----------RPILSNGNAIANYKRTVGSTAPVAHASNQATAAAVAAYHPAQPS--AAKPQINRKPVNRKPAPAVNKPK---
DebMyo1       965  VRQGLPGSTQSKPKPKG-----------RSIISN--AVAKYKLTLAATG--AGSVLQRQAHTQPARQGMQQP--VRRPALQQTRPAQQPAIQQSVNR---
PiuMyo1       966  ISRGSEPNAKNRPLPQP-----------KTKFTG-DKIAAYKRAAQNRGAGKKLLSASAAAAAYSPSQTQAAQQQQQQQQQQYHQPQQQQHQHQHQHQHQ
NabMyo1B      955  VRRGHPSGSKSRKKPKEKKQKLTESVISETNQTRPSRDISLAASAAQAAYNPSSISQRFRVQEAQPPTQPSKKTTQGVGHAIQTSHEKVFESFSDTNILT
ZbMyo1        955  VRRGHPANSTSNKKPKHVGGYVRTTIKKRRSVRKPVPPTSRTVKKPAPPPPKQINLAASAAQTAYNPNGNSSKSKTSGTTPSQQTSTRPARRTTRTTHTT
Ca_bMyo1      975  VKQGLPASSKNPKRPRGVSSKVDYSKYYNRGAARKT---------AAAAQATPRYNQPTPVANSGYSAQPAYPIPQQPQQPQQ---YQPQQSQQQTPYPT
StaMyo1       973  VRQGLPANSKNPKRPRGISGKVDYSKYYNRGAATSVASAVKPVMQRQPTHQFQQQAPVYQTPAYDTQVQQVQTSPQRRKAPPHVLTQVSSTAAAAAQAAY
ShpMyo1       971  VRQGLPANSKNPKRPRGRSGKVDYSKYYNRGAATRS-----------------SVSKPVIQRQPTHQFQQQAPVYQTPAYDTQVQQQQQQPVVQQQPVVQ
CllMyo1       972  VGQGLPPSSKNPKRPRGKQSAVDYSKYYNRGAQRSVADYAQPTVVQRPVQRQEYSIPQPSQQQYTQQSQPSQPSHHPQQHSQQHNQPKPMVSASIASAAS
MefMyo1       971  VQQGLSPNSKNPKRPRGVSSAVDYSKYYNRGVRRSNGGGQSAAAPAPAARNTGAPQPYMPPQPAARSTAQTPARVINNNVAAPVAPQRPQ----------
CnmMyo1       973  VRPGLPANSQNPKRPRGVSGKVDYSKYYNRGAARRTAAPSYSQPKPVANSGYSAQPTYQQQQQSIPQQQPVRRVPEPTSPVQHQQQQHHQQPRVVSPKTQ
DhhMyo1       972  VRQGLPGNSQNPKRPRGVAGKVDYSKYYNRGANSRSTSSYQHPAVAKPVQNNRPSYIQPPIQQTQHAAPPAQQ--TQQNVAPPVQNQSESQSRGYAPPDT
DehMyo1       972  VRQGLPGNSQNPKRPRGAAGKVDYSKYYNRGSNMRSTSSYQPPAVSKAPQSSRPSYIQPPVQQTQQRVVPPVQSQPKPQAQRYAPPDTQPQTQRHAPPDT
CatMyo1       971  VAAGLPGSSKNPKRPRGASGKVDYSKYYNRGSGRSNGFA--ASVSTAAAHRSYQPAAQASYQPAAQASYQPVAQASYQQP-------IAAPSVPQKARKP
LoeMyo1       980  VNQGLPPTSKNPKRPRAKSGKVDYSKYYNRGVRSSA---------QPAFQSQP---------TASYRSEPAYPQP-------TTQSYATQHQPQQPQVPT
MrgMyo1       972  VRPGLSPDSQNPKRPRATSSKVDYSKYYNRGGRLAPARTVPPAHPTSQQRTPVAPPSHATQQQPSYPTPVAPVHQQPNNHVRKVPPPAPSLNNNHQEAVT
ShsMyo1       968  VKPGLPSSSRNPKRPRGKSGKVDYSKYYNRG--VKKSFPVPSARPTAASQPSYKSPVEQS-YQPQVLAYQTPRPEEAKPVQRKAPPPAPVVQNQPQR---
CameMyo1      974  VTQGLPANSKNPKRPRGVSGKVDYSKYYNRGARNGTSSYQARPAAATRPSQPSYSQPESTHYQTSSPTQTASRPVHATPPGAPHSRTSSQDGPSLQVAAA
MifMyo1Alpha  973  VRQGLPATGKNPKKPRGPPSKVDFSKYYNRG---KTMKASNSYQTHNLRSQHTYNGSQGTS-PAASSAVYGNSRIHKPAPAS--LNQT---------QTY
CadMyo1       977  VKQGLPASSKNPKRPRGVSSKVDYSKYYNRGAARKTTTTTTTTAAAAAVATPTYNHPTPIVNSGYSAPQPAYPIPQQQQQQQQYQPQPQQQ-QHQTPYPI
CaoMyo1       974  VNQGLPASSKNPKRPRGASGKVDYSQYYNRGS-RTGTSSYQSRPAGTQPVKPSYSQPAP--------------------TQHQYQNSAPTQ---PKPRPV
CapMyo1       974  VNQGLPANSKNPKRPRGTSGKVDYSQYYNRGTRNGTSSYSSRPAAATTRPAQPSYSQPEQAQSRYQAPTPHQPASR------------------------
Ct_aMyo1      972  VRQGLPANSRNPKRPRGVSGKVDYSKYYNRGAARRT----------------AAPSYNQPKPVANSGYSAQPSYPQQQQQQQQQPQQPQQ-VQQPPRSP
PtaMyo1       965  VRKGNPSSSRQKKKPRGKPSHALE----NYRPTGNGNGNGYASSNGFASAVPSYQQNRHPV---------SSPSPAQNNYQNRKAPPPPAP---------
CacaMyo1B     961  VRRGNPADAKVMKKPKNKKRKVPVEYIEDVDPQPQQPVRQTTKLPDASLALAAQAVYKPDGGNRRQSGQYAHKPSKPVPKPRVKPDTTKRTVTNTIFKP
NadMyo1       955  VRRGHPAGSRSKKKPRGKGSGSGS----STTATYKRQ-SIKSTPQAPSGMKPPVARSRSKK---------PAP-PPPGSQQPSLAATAAQA---------
```

```
                          1110      1120      1130      1140      1150      1160      1170      1180      1190      1200
                 ....|....|....|....|....|....|....|....|....|....|....|....|....|....|....|....|....|....|....|....|
CnbMyo1A     1003 APVPPQIAN-GS---KPSAPVSRGGKKPAPPPPGAKKQP-APPANRKPVPQP------------------------------------------------
CdnMyo1A     1003 APAPPQMTN-GGSAAKPSAPVSRTSKKPAPPPPAAKKQP-MAAANRKPAPQP------------------------------------------------
CacaMyo1A    1003 APPVA-----------------PKTKKPVPPAPPKHSKP-ISSTTKKSAPPPPGSSQKPNTPI-------------------------------------
NabMyo1A     1003 QHMS------------------QATKKPAPPPAGSKPIASGFRKAKKPAPPPPTSKKAGVSQRAS-----------------------------------
NdMyo1A      1003 APAPPQTMN-GANTIKPTAPVSRTNKKPAPPPPGAKKQP--NTTERKAAPQPAQSGLQDT----------------------------------------
CnbMyo1B     1002 SLAAVAAQAAYHPNGFKPPARTQSTKKPAAPVKRSPVK-----TVKKVSATHATAIPAVPISNNRPTSSNFKFEQ-------------------------
CglMyo1A     998  QNTTVSQSLNGGMNVKPQTPASRSAKKPAPPPPGSKKPAPQPMAKKPAPHPTPQAQMQTQTQIPASQSSATQSS--------------------------
Sab_aMyo1B   992  SRVPAQ------TQRVPTAPTSRNSKKPAPPPPGTQSRVNVRSSNPAPAPTQRSAAAPA-----------------------------------------
Sc_cMyo1B    992  SIAAAQ--------HVPTAPASRHSKKPAPPPPGMQNKAATRRSVPNPASTLTASQSNARPSPPTAATRATPAAT-------------------------
SaaMyo1B     992  VSGISPQVQRASVTPASG-ISPQVQRIPVIPVSRNSKKPAPPPPGTQNKVNTRNSVPKPAPTQNNTAT--------------------------------
SakMyo1B     952  SSTPAQ--------RVPVAPVSRNSKKPAPPPPGMQNKVSAKRFAPTPAAAARSATAAVT----------------------------------------
SmiMyo1B     992  SNTPFQ--------RVPVAPASRNSKKPAPPPPGVQKKVTTKSRVPTSIPTSSKGNARPSP---------------------------------------
Sap_aMyo1B   992  SNVPAQ--------HVPVAPTPRHSKKPAPPPPGMQNKATTRRSAPNPGSNPITSQTNVRPAVPTDATRAT-----------------------------
NacMyo1      993  SISQQP------RSPKPVATSRPATKKVAPPPPGSQNLAASAAQAVYHPNGNSKPAAPNPGTKPQHRPAKKRVAPPKPQQAQKPTQRPKEQE--------
KaaMyo1      1002 INNRQKPSPSANVARPSAPVSRNTGKKPAPPPPGAQGRALAATAAQAAYRPNGDSKPAPSAPNPKPNTAIQQSRSAP-KPTLAKTQ-QKPQAQAR-----
VpMyo1       991  QS------------SQPSAPVSRKTKKPAPPPPGSKKLSSSVAQTASRPQPVANSARGAAQPQATPQPAQVTQPQQKK----------------------
TtpMyo1A     990  HKSS----------SKPSAPVSRTSKKPAPPPPGSK-------------KPVQN------KPVAQQVPVQTQQTQLNN----------------------
TtpMyo1B     990  HKSS----------SKPSAPVPRTSKKPAPPPPGSK-------------KPVQN------KPVAKQVPVQTQQTQLNN----------------------
TtbMyo1A     1065 TTAVPKPSEDERHGFGHNLKKSFGFGHAKKQPPPPAPAKSSQRRHVDSPVKVTTQNNQTNSSVPKAPRAPEPKPQPRASSTPSAPSAPKPASNIPAAPAM
TtbMyo1B     1026 -------------------------------PPP-----GSQR--------IQQQQSQPN---------PTPKPKPHVS---------------------
ErgMyo1      1054 NPHKLGGNMDNSSAAYGNASALPNSAPSQAPKPASRPVPKPAPRPGPKPGPKPGPKPGPKPAPKPMPRPAKSVENSVPRPNAIEQGK-------------
ErcMyo1      1047 YRPARP---------AKTAGHELPAQVRHQAPQ-QQQQQQQHAKQPQNPYAKKPVNSSTPGKTPVQRPNLGTSTESAK----------------------
HsvMyo1      1044 AAAAQSVYHPQQQQQQQQQQQQQSVARPQPQARPQPQVHSQPQSTPKPAAQSAI----------------------------------------------
KlMyo1       1035 ASAAQTAYHPQQASHARVPSTNNAHTQHNRQPAQQAAQPVQQAAQPAATTSQPTRR--------------------------------------------
KmmMyo1      1052 ATAAQAVYHPQTSQRPNKAAAPSRPQQPQQQQQQQQQQQQQQQQQQQQQQQQQQQQPAKKS---------------------------------------
KlwMyo1      1025 ASAAQSAYQPRPTSNNAHRPVPQQIQQPQKATVVPQAKK-------------------------------------------------------------
KaMyo1       1030 ATAAQAAYHPHMNVTPTKRRPAPIPPQIQQQQQQQQQQQQQQQQQPKPTSQAKKAIPP------------------------------------------
KnMyo1       1034 AVAAQAAYHPNGGVPARTPSTAHRPQPPQPQSRSTNTKKPAPQPPVPQQQKHPQPTPAVAPTPVKAN---------------------------------
LwMyo1       1049 ATAAQAAYHPQQVTENRANGTNTTSRPTHAYSSLDPLHQQGIKTTTGAANVSQGSKSS------------------------------------------
LatMyo1      1046 ATAAQAAYHPQPAVHNSEQEAGVNHGTPPRQTPMGASQGSRSRQEPKSNFATASSPAKSA----------------------------------------
Lak_aMyo1    1056 ASAAQVAYHPSQNSQQARHQQNAQQLQQAQQVQQVQQAQQAQQTQQQTPSQNTNPTVVPQKKAA------------------------------------
Sab_aMyo1A   1022 ATAAQAAYNPKPNSS-GKTVTPKPAPKPATKPSLMHSSKSPSKERNQEKNVSSIHKSLSSKGTKTPVPLPKKEVESQKTPKETTSNV-------------
Sc_cMyo1A    1021 ATAAQAAYNPKP----DKTVPIKSSAIPAAKVSSKHSSKPSSKEKVAVKKASSSHKSSSAKQNQVSMPPSKGVEKNKEPLKETTATATA-----------
SaaMyo1A     1022 ATAAQAAYNPKP----DKTV----SAKPVAKHNSKDNSKAPTKGRNIVRKVSQTHKAPSGKESKVLVSSSQKVNESKEPLKEATA---------------
SakMyo1A     742  ATAAQAAYNPKPHKTVSTKSAPKPAAKPAPKPIPKPNSKVPTKENAVVNNSSSSHKTSSTNEGKSSAPSPKEADENEEPFKEAAGNI-------------
SmiMyo1A     1022 ATAAQAAYNPKS----DKTALSKSFANPAARTSSKNNSKAPIKEKTILKKNTANNETSSAKENLISIPSSKKVNENQEPLNETTTNI-------------
Sap_aMyo1A   1022 ATAAQAAYNPKP----DKTVPRKASANPGTKPSSKYNSKSLSKENTILKKASSSHKSSSAKENQVSMPPSKRVNENKEPLKATTADI-------------
ZrMyo1       1017 ASAAQTAYHPSNLGASKPVPPPAQRSMNSSVPRPAPRTMPVRNARTTAPRQPAAPTAAYQPAAPTAAYQPAAPTTAYQPATVPRATPNPADTAQAPTSNP
TodMyo1      1011 APVSRTASK-------KPVPPP-----------PAAKKPAASNLAATAAQAAYHPNGVSRAAAPSQNATPASKPAVSQNTTPSVSRPQEENRGSQTTTFP
CglMyo1B     1010 ASAAQAAYHPKGIRSPTSTEQKSPSKSKPITKTRKPPVSSPVRNTSKTISNSKVYSAPKASVTKRTQDTVSVSKTSVKDDVTQEKNAIIQ----------
WaMyo1Alpha  1028 SVAAASAAYHPQ---QPAVHQQTSQRKVPPPRPAKKQVPPPRPAQRQQPAAVPQPVQSTPAVKST-----------------------------------
WicMyo1      1031 NNNTATAAAAAAYHPNQQQQQQVQPSIPPKPQQRKVPPPRPAKKIVPQPPPQRQQQFSPQPQVQSQPVQQPQVQQQPVAKSTPP----------------
CyjMyo1      1028 AIAAASSAYHPGG--QPAPPQS---RKAPPSRPQKKAVPQPPKPQPQVQQPAVAVQQSRQAPP-------------------------------------
YlMyo1       1058 PRSGQGQQ----------------------------------------------------QQQHHQAYQQPTAAQPAATSYSPAPAKAAPP---------
CdnMyo1B     1054 TATNIPKVPKAKNTFIDSKHDFGSGSVAAASTSIPASN--------------------------------------------------------------
Kop_bMyo1    1058 IQPTQPVQPTQHSQPVQPTQRTQPVQRSQPTQPIEPVKKNIPPPPP------------------------------------------------------
NdMyo1B      1053 VNVPKVPKSKGTLVDSNFKSAGNTTNTPIPDSTYSNTADKGEMQT-------------------------------------------------------
KcMyo1       1058 PPPKQPVASSSAKVAPP-----------------------------------------------------------------------------------
OgpMyo1      1046 -----PAPPQSRQTHVEPARAPQAAVHPVQHAQSSQANPVPPPPPPP-----------------------------------------------------
DebMyo1      1044 --------HESQRQPVQPS-QPQIKGRPVVNKRL-----APPPPPP------------------------------------------------------
PiuMyo1      1054 HQHQHVQQASVPAPPSANNKPRLSKPAAPQPRPKPAAPVSRNRQSMQPTSAQTSVAKPAPAPPAPPAPP-------------------------------
NabMyo1B     1055 STKINSAPIREDTLGLNVATSLS-----------------------------------------------------------------------------
ZbMyo1       1055 SIRSSNSQQGTSNRTSQQSSVAAPPTKRAS----------------------------------------------------------------------
Ca_bMyo1     1063 QSSIPSVNQNQSRHPQRKVPPPAPSLQVSAAQAALGKSPTQQRQTPAHNPVASPNRPASTTTTTTTSHTSRPVKKTAPAPPVKKTA--------------
StaMyo1      1073 GQANPQAQAQAQAQPKKTPVPTPVVPA-------------------------------------------------------------------------
ShpMyo1      1054 QQTSPSQQR-------RKVPPPAPVNVAAAAAQAAYGQKPQ------------TVQPAAQAKAQTPTPVVPSRAKKTAPAPPAKKTA-------------
CllMyo1      1072 AQPMPQRRKAPPPAPSHSEAIAAATAATTAHPELQH------------------GVRPPAKQNYEPAAKTQTRKTAAPTPPAKKVA--------------
MefMyo1      1060 --GHSKPAPMAPSRPQPHQNSQPAR------------------------------VVPPARP---------VQKNPAPAPPARKVAP-------------
CnmMyo1      1073 RKAPPPAPSLQVAAAQAALGKSPSQ-----------QQQHHH----------------------------------------------------------
DhhMyo1      1070 QTHAHPEQAAQAAQAAFHHTAPPTQNTSQRKVPPPAPSSFGQQP--------------------------------------------------------
DehMyo1      1072 QPHAHPEQAAQAAQAAFHHTAPQAQNTTQRKVPPSAPG-----------------SYGQQAPTQKPSAPSRPARKTAPAPPAKKNV--------------
CatMyo1      1062 P-----------------------TPQAKPAKKAAAPPPAPR------------------------------ATEQAKKAAPPPP---------------
LoeMyo1      1055 RTAS------------RKAPPPAPSTQTAAQVSTSPQAARKPAAPARPAKKIAPQPPVKKAVSPQP-----PAKKTVAAPP-------------------
MrgMyo1      1072 AATA-----------------------------------------AMNHVNIQQPATVVQNHNSNPTAPSRPAKKAAPAPPVKKTA--------------
ShsMyo1      1061 --------------------------------------------------------------QPQAIEPVRPVKKTAPSPPVRKTAPPPPP---------
CameMyo1     1074 QAALGKTHHQPAQVTSTKKPPARPAKKVAPPPPA------------------------------------------------------------------
MifMyo1Alpha 1058 PEPTVPTAPQQT--------------------------------------------------SNQKPVPPRPVKKTAPAPPAKKNVAPPPP---------
CadMyo1      1076 QSTIPSVNHNQPQQPQRKAPPPAPSLQVSAAQAAIGKSPTQQRQTPAHNPIASPNRPT--TTTTTTSHTGKPVKKIAPAPPMKKTA--------------
CaoMyo1      1050 HATSPTATHTRNQSHD------GPSLQVAAAQAALGQGQYNNNRTQPAQPTSPRRSPAP-SRPAKKVAPPPPTKKATSPPPKKNVA--------------
CapMyo1      1049 ---PVHSIPPAAPTHSRNQSHDGPSLQVAAAQAALGQKQQNNRPQPAQPTSPRKAPAPARPAKKVAP--PPPTKKATS-PPPKKTASQSV----------
Ct_aMyo1     1055 VQHQQPQQRVVSPKTQRKAPPPAPSLQVAAAQAALGKSPQHPQPQQHHAQPQPQHHQE-VSSPTKPKAPSRPVKRTAPAPPVKKS---------------
PtaMyo1      1042 -----------AMQQQPSARSQAVSAATAAYSHTNSKPPVRANKPPPPQPIAKKKVPP------PRPQPKQAAAASTPVPPP------------------
CacaMyo1B    1061 TTILSDAKGKKSIHLNNNDRASAISDSK------------------------------------------------------------------------
NadMyo1      1030 -----------AYHPNGSSKNQAPSPVGIKPSAR-PQVHSRPKPKPKPQPPVQQQQPQ------QQRQPVAQAKSAVVPPPP------------------
```

```
                       1210      1220      1230      1240      1250      1260      1270      1280      1290      1300
                   ....|....|....|....|....|....|....|....|....|....|....|....|....|....|....|....|....|....|....|....|
CnbMyo1A      1049 ----------------------------------------------------AAVSQPAVQTTPQPAAQPSAQTTNGGSSIPPPPPPPPMQSSEPKFEAA
CdnMyo1A      1052 ----------------------------------------------------AAASQPAVQATPQPAAHANAQASNSGSSIPPPPPPPPMQSSEPKFEAA
CacaMyo1A     1047 --------------------------------------------------------PAAQPIGQSSFSSNSQNNVERKQNSPPPPPPPQASNTDPKYQAA
NabMyo1A      1049 ---------------------------------------------------TVLNGNSVSSVSPVAQPEPQQQQHQNNVPPPPPPPPPSFVSADPKYEAA
NdMyo1A       1059 -------------------------------------------------------------------------VQANAAGTQPTPPPPTIQSTEPRFEAA
CnbMyo1B      1071 ---------------------------------------------------KTSQAPAGPTVATINSNASATATIAPSAPPAPPMPSSLDTPPDDRYEAA
CglMyo1A      1071 --------------------------------------------------------------------------------IPPPPPPPPSKTSEPQFEAA
Sab_aMyo1B    1044 --------------------------------------------------------------------ATVQANRQTSVPPPPPPPPPASKPKEPMFEAA
Sc_cMyo1B     1058 -----------------------------------------------------------------PAAAAMGSGRQANIPPPPPPPPPSSKPKEPMFEAA
SaaMyo1B      1058 ------------------------------------------------------------------APAVVRTNRQANIPPPPPPPP-VSKPKEPMFEAA
SakMyo1B      1003 ----------------------------------------------------------------------MRTNGQAHVPPPPPPPPPSSKPKEPMFEAA
SmiMyo1B      1044 ------------------------------------------------------------------TAATAQVNRQDNIPPPPPPPPPASKPKEPMFEAA
Sap_aMyo1B    1054 ------------------------------------------------------------------PVGATTKSNRQANIPPPPFIPPTTKPKEPMFEAA
NacMyo1       1078 ----------------------------------------------------------------------RHIQKPAAAAVPPPPPPPPMGQTEPKFEAA
KaaMyo1       1094 ---------------------------------------------------------------------QQQQQPSTASHPPPPPPPPPMQSSEPKYEAA
VpMyo1        1056 -------------------------------------------------------------------------------VAPPPPPPPPMQSSEPKYEAA
TtpMyo1A      1038 -------------------------------------------------------------------------------IPPPPPPPPPVQSSVPKFEAA
TtpMyo1B      1038 -------------------------------------------------------------------------------IPPPPPPPPPVQSSVPKFEAA
TtbMyo1A      1165 PTSLGGAAQPASSRPAAPLPGQAQSQPRPATITPAKPRSANVMPAKPRQTNVMPAKPRQTNVMPAKPRQTNMATTQQATPAPPPPPPPPAVAPQKTWEAA
TtbMyo1B      1052 ------------------------------------PRKA-----------VQPPLARKT------------------THKAPPPPPPVAQPVFPKYEAA
ErgMyo1       1140 ---------------------------------------------------------------------------TAPPPPPPPPPPPAAVPSEPVYEAA
ErcMyo1       1114 ---------------------------------------------------------------------------TAPPSPPPPPPPPAAKPAEPRYQAA
HsvMyo1       1097 ----------------------------------------------------------------------VNGGAVNKPGPPPPPPPPPAQKQDPKYKAA
KlMyo1        1090 -------------------------------------------------------------------------------VAPPPPPPPPTKQNIPKFQAA
KmmMyo1       1112 -------------------------------------------------------------------------------VPPPPPPPPPSNQNIPKFQAA
KlwMyo1       1063 -------------------------------------------------------------------------------AAPPPPPPPPVKESIPKFQAA
KaMyo1        1087 --------------------------------------------------------------------------------PPPPPPPPQAKEVIPKYQAA
KnMyo1        1100 ------------------------------------------------------------------------ANQGGSIPPPPPPPPPAAKPSEPMFEAA
LwMyo1        1106 -----------------------------------------------------------------------------VPPPPPPPPPAVAKPVFPKFTAA
LatMyo1       1105 -----------------------------------------------------------------------------VPPPPPPPPPAS-KPSVPKFEAA
Lak_aMyo1     1119 -------------------------------------------------------------------------------PPPPPPPPPAAKPAEPMYEAA
Sab_aMyo1A    1107 --------------------------------------------------------------------------------PVPPPPPPMGEPEDPKFEAA
Sc_cMyo1A     1105 ------------------------------------------------------------------------------NIPIPPPPPPMGQPKDPKFEAA
SaaMyo1A      1098 ------------------------------------------------------------------------------KVPIPPPPPPMNQPEDPKFEAA
SakMyo1A       828 --------------------------------------------------------------------------------PIPPPPPPISQPRDLKFEAA
SmiMyo1A      1104 --------------------------------------------------------------------------------PIPPPPPPMGQTEDPKFEAA
Sap_aMyo1A    1104 --------------------------------------------------------------------------------PTPPPPPPMGQPEDPKFEAA
ZrMyo1        1117 VP-------------------------------------------------------------------HVQQSNGAAPPPPPPPPMPKAAPAEPLYEAA
TodMyo1       1093 V-----------------------------------------------------------------------QTKNIPPPPPPPPPMAKQP--DPKFEAA
CglMyo1B      1099 ---------------------------------------------------------------------------TEEKQNYSLPENIPQSSQTDSYQAA
WaMyo1Alpha   1089 --------------------------------------------------------------------------------PAPPPPPPAAKPSDPTFKAA
WicMyo1       1114 --------------------------------------------------------------------------------PPPPPPAAASKPKDPTFKAA
CyjMyo1       1085 --------------------------------------------------------------------------------APPPPPAAVAKPEFPTYKAA
YlMyo1        1096 -------------------------------------------------------------------------------PPPPAPPAAPAAPAEPTYKAL
CdnMyo1B      1091 -----------------------------------------------------------------GMDNSSKQSQVPAAPPAPPMPSSFDSIPDDRYEAA
Kop_bMyo1     1103 ------------------------------------------------------------------------------------PPPAVETPKYPTYKAA
NdMyo1B       1097 ----------------------------------------------------------------------EAQEQISSANPGPPMPPSMESKPDDRYAAA
KcMyo1        1074 -----------------------------------------------------PPAAKRKPAAPTPSHSTPATASVTPTPPPPPPPPPAAAPAYPKYKAA
OgpMyo1       1088 ------------------------------------------------------------------------------------PPAAAAKPKWPTFKAA
DebMyo1       1076 --------------------------------------------------------------------------------------LQQQKPKWPTYKAA
PiuMyo1       1122 --------------------------------------------------------------------------------------AAVAKPKWPTYRAE
NabMyo1B      1077 ------------------------------------------------------------------------------QNSAPAPPPLPSKSELPKYEAL
ZbMyo1        1084 -------------------------------------------------------------ASNYDAAPQRNSSASSPPPPPPPPPMAKAAPAEPLYEAA
Ca_bMyo1      1148 ---------------------------------------------------------------------------------PPPPPPTLVKPKFPTYKAM
StaMyo1       1099 ------------------------------------------------------------------RNKKIAPAPPAKKNAPPPPPPALSKPKHPTYKAM
ShpMyo1       1121 ---------------------------------------------------------------------------------PPPPPPALSKPKHPTYKAM
CllMyo1       1139 --------------------------------------------------------------------------PKPPPAPPAPPSAPVQKPKYPTYKVL
MefMyo1       1106 ------------------------------------------------------------------------------PPPPPPPALAVAKPKHPTYKVL
CnmMyo1       1103 --------------------------------------------------HVASPTKPQAPSRPVKKTAPAPPVKKSPAPPPPPP--SLSKPKFPTYKAM
DhhMyo1       1113 -----------------------------------------------------SVQKPIAPSRPAKKTAPAPPQKKSVPPPPPPVVSQPPKPKFPTFKAA
DehMyo1       1140 -----------------------------------------------------------------------------APPPPPAAASPPPKPKFPTYKAA
CatMyo1       1093 ------------------------------------------------------------------------------PPAPPAAVSAPKTPKYPTYKVN
LoeMyo1       1118 ---------------------------------------------------------------------------------PPPPPPALSKPKHPTYRAM
MrgMyo1       1116 --------------------------------------------------------------------------------PPPPPSLSAAKPKWPTFKAN
ShsMyo1       1090 ---------------------------------------------------------------------------------PPPALSAPAKPKFPTYKAM
CameMyo1      1107 -------------------------------------------------------------------KKATSPPPKKTVAPPPPPPPALSKPKFPTYKAM
MifMyo1Alpha  1098 ----------------------------------------------------------------------------------PPPPSLSAKPKFPTYKAA
CadMyo1       1159 ------------------------------------------------------------------------------PPPPPPPPPTLVKPKFPTYKAM
CaoMyo1       1128 -------------------------------------------------------------------------------PPPPPPPPALSKPKFPTYKAM
CapMyo1       1133 --------------------------------------------------------------------------------PPPPPPPALSKPKFPTYKAM
Ct_aMyo1      1138 -------------------------------------------------------------------------------PAPPPPPPSLSKPKFPTYKAM
PtaMyo1       1107 ---------------------------------------------------------------------------------PPPPPQVKKEPEFPTYKAA
CacaMyo1B     1088 ------------------------------------------------------------DQKNTISEMSIPKSQSPIFDTPPPPPPPPMPTELPRYEAL
NadMyo1       1094 ---------------------------------------------------------------------------------PPPPPQASSEP---KFEAA
```

```
                     1310      1320      1330      1340      1350      1360      1370      1380      1390      1400
                ....|....|....|....|....|....|....|....|....|....|....|....|....|....|....|....|....|....|....|....|
CnbMyo1A      1098 YDFPGSGNPSELPLKKGDVVYITQQEPSG-WSLAKTLDGSKSGWVPTSYMTEHKGSATPAPPAPAASQSFT-----------------------------
CdnMyo1A      1101 YDFPGSGNPSELPLKKGEVVYISKQEPSG-WSLAKTLDGSKSGWVPTAYMTEHKGATPAAPPAPAAPQAT------------------------------
CacaMyo1A     1092 YDFVGTGSPSELPVKKGDVVYITRQEPSG-WGLARSLDGTATGWVPVAYMVEFKEGSTPTATNSNTTPG-------------------------------
NabMyo1A      1099 YDFPGTGAPSELPLKKGDVVYVSRQEPSG-WSLGKTLDGSKEGWVPTAYMVEYSSPTNASTQPSSQT---------------------------------
NdMyo1A       1087 YDFPGSGNPNELPLKKGEIVYITKQEPSG-WSLARTLDGSKSGWVPTAYMTENTPSGGPKPPVVAT----------------------------------
CnbMyo1B      1121 YDFPGSGNPSELPVKKGDIVHITKNEPSG-WSLAKTLDGSREGWVPTSYIVKYSGAQATAPPAPSAP---------------------------------
CglMyo1A      1092 YDFPGSGNPSELPLMKGDIVYITKEEPSG-WSLAKTLDGSKSGWVPTAYMVKHEGAKAPPPAPAV-----------------------------------
Sab_aMyo1B    1077 YDFPGSGSPSELPLKKGDVVYITREEPSG-WSLGKLLDGSKEGWVPSAYMKPHS----------------------------------------------
Sc_cMyo1B     1094 YDFPGSGSPSELPLKKGDVIYITREEPSG-WSLGKLLDGSKEGWVPTAYMKPHS----------------------------------------------
SaaMyo1B      1092 YDFPGSGSPSELPLKKGDVVYITRGEPSG-WSLGKLLDGSKEGWVPTAYMKPHS----------------------------------------------
SakMyo1B      1034 YDFPGSGSPSELPLKKGDVVYITREEPSG-WSLGKLLDGSKEGWVPTAYMQPHS----------------------------------------------
SmiMyo1B      1079 YDFPGSGSPSELPLKRGDVVYITREEPSG-WSLGKLLDGSKEGWVPTAYMKPHS----------------------------------------------
Sap_aMyo1B    1089 YDFPGSGSPSELPLKKGDVVYITREEPSG-WSLAKLLNGSKEGWVPTAYMKPHS----------------------------------------------
NacMyo1       1109 FDFAGTGAASELPLKKGEVVYITRQEPSG-WSLAKTLDGSKQGWVPTAYIAEVKG---------------------------------------------
KaaMyo1       1126 YDFPGSGAPSELPLKKGDVITVSRQEPSG-WSLGKLLDGSKEGWVPTAYIAEYKG---------------------------------------------
VpMyo1        1078 YDFPGSGSPSELPLKKGEVVYITREEPSG-WSLAKTLDGSREGWVPTNYVVKHQGGSVPPP---------------------------------------
TtpMyo1A      1060 YDFPGSGNPSELPLKKGDVVFISKQEASG-WSLAKTLDGAKEGWVPTNYIIAYTGSSTPAP---------------------------------------
TtpMyo1B      1060 YDFPGSGNPSELPLKKGDVVFISKQEASG-WSLAKTLDGAKEGWVPTNYIIAYTGSSTPAP---------------------------------------
TtbMyo1A      1265 YDFPGSGAATEMPLVKGDIVIVTENVESG-WSLAKKLDGSAEGWVPTAYLAERAESSAPTP---------------------------------------
TtbMyo1B      1088 YDFPGSGAPSELPVKKGDVIYITKEEPSG-WSLGKTLDGSKEGWVPTAYITPYYESVSQIP--------------------------------TSSPSLV
ErgMyo1       1166 FDFPGSGSPNEFPLKKGDRIYVTRQEPSG-WSLAKALDGSKEGWVPTAYIVESKA---------------------------------------------
ErcMyo1       1140 YDFAGTGSGSELPLRKGDIIYISKSDPSG-WSLAKTLNGSKEGWVPTAYIVEYKET--------------------------------------------
HsvMyo1       1128 YAFVGTGSPSELPLTLGQVVFISRHEPSG-WSLAKTLDGSKEGWVPTAYITEVTESASVPVSQPQQQTVQQVQQVQQVQQVQ------------------
KlMyo1        1112 YDFTGTGSASELPLSKGTVITVSKQDPSG-WSLGKLLDGSKEGWVPTNYIVEYKESSGPPPPPAPPVA--------------------------------
KmmMyo1       1134 YDFTGTGSPSELPLTKGTVITVSKQDPSG-WSLGKLLDGSKEGWVPTNYIVEYKESQAPPAPPAPPAA--------------------------------
KlwMyo1       1085 YDFTGTGSASELQLTKGTIISVSKQDPSG-WSLAKLLDGSKEGWVPTNYIVEYVESSGAPPPPPPPAA--------------------------------
KaMyo1        1108 YDFVGTGSASELPISKGTIVTVSKKDPSG-WSLAKLLDGSREGWVPTNYITEYKESSIPVPPPPPPSF--------------------------------
KnMyo1        1129 YDFPGSGAPSELPLKKGDVVYVTRQEASG-WSLGKTLDGSRQGWVPTAYMVEHTGSRGGTVTPSPTPAA-------------------------------
LwMyo1        1130 YEFVGTGSPSELPLSRGDVVYITKQEPSG-WSLAKTLDGTKEGWVPTSYMTEFHE---------------------------------------------
LatMyo1       1128 YDFVGTGSPSELPLSKGDIVYISRQEPSG-WSLAKTLDGAKEGWVPTSYMTEHKG---------------------------------------------
Lak_aMyo1     1141 YEFVGTGSPSELPLKKGDVVYISRNEASG-WSLAKTLDGSKEGWVPTAYM--------------------------------------------------
Sab_aMyo1A    1128 YDFPGSGSASELPLKKGDIVYIARDEPSG-WSLARLLDGSKEGWVPTAYITACKGSTKTVPTPEA-----------------------------------
Sc_cMyo1A     1128 YDFPGSGSSSELPLKKGDIVFISRDEPSG-WSLAKLLDGSKEGWVPTAYMTPYKDTRNTVPVAAT-----------------------------------
SaaMyo1A      1121 YNFPGSGSPSELPLIKGDVVFISRDEPSG-WSLARLLDGSKEGWVPTAYMTPCKGIRDTASTPAA-----------------------------------
SakMyo1A      849  YDFPGSGSPSELPLKKGDIVFISKEEPSG-WSLARLLDGKGEGWVPTAYMIMYKETRDTIPAVEA-----------------------------------
SmiMyo1A      1125 YDFPGSGSPSELPLKKGDVVFISRDESSG-WSLARLLDGSKEGWVPTAYMTPYTGASDTISAVEV-----------------------------------
Sap_aMyo1A    1125 YDFPGSGSSSELPLKKGDIVFISRDEPSG-WSLAKLLDGSKEGWVPTAYMTPLKETRRTVSITAT-----------------------------------
ZrMyo1        1150 YDFPSSGSPSEMPLSKGEVVYITRNEPSG-WSLAKTLDGSREGWVPTAYMTEHAVG--------------------------------------------
TodMyo1       1120 YDFPGSGAPSELPLKKGDVVFITRQEPSG-WSLGKLLDGSKEGWVPTAYMTEYHEAH-------------------------------------------
CglMyo1B      1125 YDFPGSGNPSELPLQKGDIIYVSKSDPSG-WSLASTLDNSKEGWVPTSYIVKYN----------------------------------------------
WaMyo1Alpha   1110 YDFVGTGSASELPIAKDQVVYILKKEANG-WWLAKTLDGSKEGWVPGSYVVECEPP----------------------------------------MTNG
WicMyo1       1135 YDFVGTGSASELPISKDEIVYILRKEGNG-WWLAKTLDGSKEGWVPGSYVVECEPP--------------------------------------------
CyjMyo1       1106 YDFVGTGSASELPISKDQVVYILKKENNG-WWLAKLLDGSKEGWVPGAYVVECAPPSTG------------------------------AKAAPPAPPAP
YlMyo1        1118 YDYVANGL-NQLSISAGEQVLISVKEDQG-WWLAKRMDGSEEGWTPAAYLEEVQGGAAAPPPA-------------------------------------
CdnMyo1B      1127 YDFPGSGNSSELPLKKGDVVHITKNEPSG-WSLAKTLDGSREGWVPTSYIVKYSGSQPTQATPAVTADATAPV---------------------------
Kop_bMyo1     1120 YEFQGTGSPSELPIQKDQIVYILQKEENG-WWLAKTLDEAKEGWVPAAYVVECAPPATKAVPVQAQPAAPQ-----------------------------
NdMyo1B       1128 YDFPGSGNPSELPLKKGDVVHVTKNEPSG-WSLAKTLDGTREGWVPTSYIAKCADTPPEVISSVQTASVQLQPQATSH----------------------
KcMyo1        1122 YEFAGSGSPSELPVAKDQVVYILKKETNG-WWLGKTLDTGKEGWVPAAYVVEFSESVTPTPAPVAYSAPQAASNGNGN----------------------
OgpMyo1       1105 YDFPGSGSPSEFPVTKDTVMYILQDQGAG-WSLAKSLDETREGWVPTAYIVPCDPPAHILKGGSSAPAAPPAPSIATSNSTTNGSARNG-----------
DebMyo1       1091 YDFPGSGSPSELPVTKDTVIYVLQDAGNG-WSLGKTLDESKEGWVPTAYIVKCEPPK-----SKSRPAPPPPPSLSTKNRSQK-----------------
PiuMyo1       1137 YDYPGNNGS--MALTKGDIMYILQSEPSG-WSLAKTLDEATEGWVPTAYIVECEPPS-----LLGGNVQSAPTSTQAP----------------------
NabMyo1B      1100 YDFPGTGAATELTLIKGDIVYISKNDPSG-WSLAKTLDESKEGWVPIAYMAEYKPAVTEMPVQEQQIQQEYNTHTTPNTS--------------------
ZbMyo1        1124 YDFPGSGSPSELPLAKGDVVYITRNEPSG-WSLAKTLDGSKEGWVPTAYVTEHVGGVSTVSSTTTQAASSTPAATFAQSTV-------------------
Ca_bMyo1      1168 FDYDGS-VAGSIPLVKDTIYYVTQVNGK--WGLVKTMDETKEGWSPIDYLKECSPNETQKSAPPPPPPPPAA----------------------------
StaMyo1       1134 YDYDGS-VPNTYPLTKHEIYYVEEKNDN--WALVKKLDESSKGWSPVAYLQECEPPQGKSAPPPPPPVQAQAQAQAQTQAREPISNPTSTTSSYT-----
ShpMyo1       1141 YDYDGS-VPNTFPLTKDEVYYVEEKNDN--WALVKKLDESAKGWSPVAYLQECAPPTGKASAPPPPPPPPAQAAQP------------------------
CllMyo1       1166 YDYDGS-VSGSVPLVKDDIMYVVNVNGQ--WGLVKDLKETIEGWAPFDYMKEVDPPANLFGGGKS-----------------------------------
MefMyo1       1129 YDYDGS-VSGSVPLVKEDVVYVVSVNGK--WGLVKDLKETREGWAPLDYMKEIEPPADLFGGAKVAAP--------------------------------
CnmMyo1       1152 YDYDGS-VAGSIPLVKDTVYYVVQINGK--WGLVKTMDETREGWSPIDYLQECSPNDAQKSAPPPPPAQAQTQTQQSTSNPASSA---------------
DhhMyo1       1161 YDFQGTGSASELPISKDTIVYITRKEDNG-WWLAKTLDESKEGWVPAAYVVECDPPASTSAGNAKSAPPPPPQLSSASQAQQSQQQ--------------
DehMyo1       1164 YDFQGTGSASELPISKETIVFITRKEDNG-WWLAKTLDETKEGWVPAAYVVECDPPANSPAGN-------------------------------------
CatMyo1       1116 FDYDGS-VAGSIPLKKDDIVYVANENGQ--WGLVKDLEETKEGWAPLSYMEKCEPPASIFGTR-------------------------------------
LoeMyo1       1138 YDYDGS-VAGSVPLVKDTIYYVLQINGK--WGLVKTMDETQEGWSPIDYLQEESSPSASAATQSYAPTTAS-----------------------------
MrgMyo1       1137 YDYDGS-VSGSMALSANDIVYITQNNGQ--WSLAKSLDESKEGWVPTAYISECPPPSNLGGSKSPPPPPPP-----------------------------
ShsMyo1       1110 YDYDGS-VAGSIPLVKDVVYYVESINGK--WGLVKTLDESKEGWSPIDYLSQCEPPSSLFGAANPPSRPVAPSQPK------------------------
CameMyo1      1141 FDYDGS-VPGSVALTKDTIYYVTQVNGK--WGLVKTMDESQEGWSPIDYLQECSPNANNVYSNSNTSAPSNPVSTASSNTNTT-----------------
MifMyo1Alpha  1117 YDFQGTGSGSELPISKDTVVYITRKEASG-WWLAKTLDEKSEGWVPAAYVVECDPPS---GATSKPSSSQAEEPAS------------------------
CadMyo1       1182 FDYDGS-VAGSIPLVKDTIYYVTQINGK--WGLVKTMDETKEGWSPIDYLKECSPNEVQKTVPPPA----------------------------------
CaoMyo1       1150 FDYDGS-VPGSVALTKDTIYYVTQVNGK--WGLVKTMDETQEGWSPIDYLQECSSN--------------------------------------------
CapMyo1       1154 FDYDGS-VPGSVALTKDTIYYVTQVNGK--WGLVKTMDESQEGWSPIDYLQECSPNANNIYSNS------------------------------------
Ct_aMyo1      1160 YDYDGS-VAGSIPLVKDTVYYVSQINGK--WGLVKTMDETKEGWSPIDYLQEVSPHSGTKSPPPAPPMQQQQQQ--------------------------
PtaMyo1       1127 FDFVGTGSASELPISKETIVYILRQEDANGWWLAKTLDGSKEGWVPAAYVVKCDPPSSGSTSAASSSAPATN----------------------------
CacaMyo1B     1129 YDFPGTGSANELILKKGDVLYVSKEDPS-GWSLADTIDKSKQGWVPTSYITLQQSPIFSVPPTEVPSKIDSMANERQ-----------------------
NadMyo1       1111 YDFPGSGAPSELPLKKGEVVYITRQE-QNGWSLAKTLDGSKTGWVPTAYMTEAKITSSTSTPITVPNTTTTVTQQVQQT---------------------
```

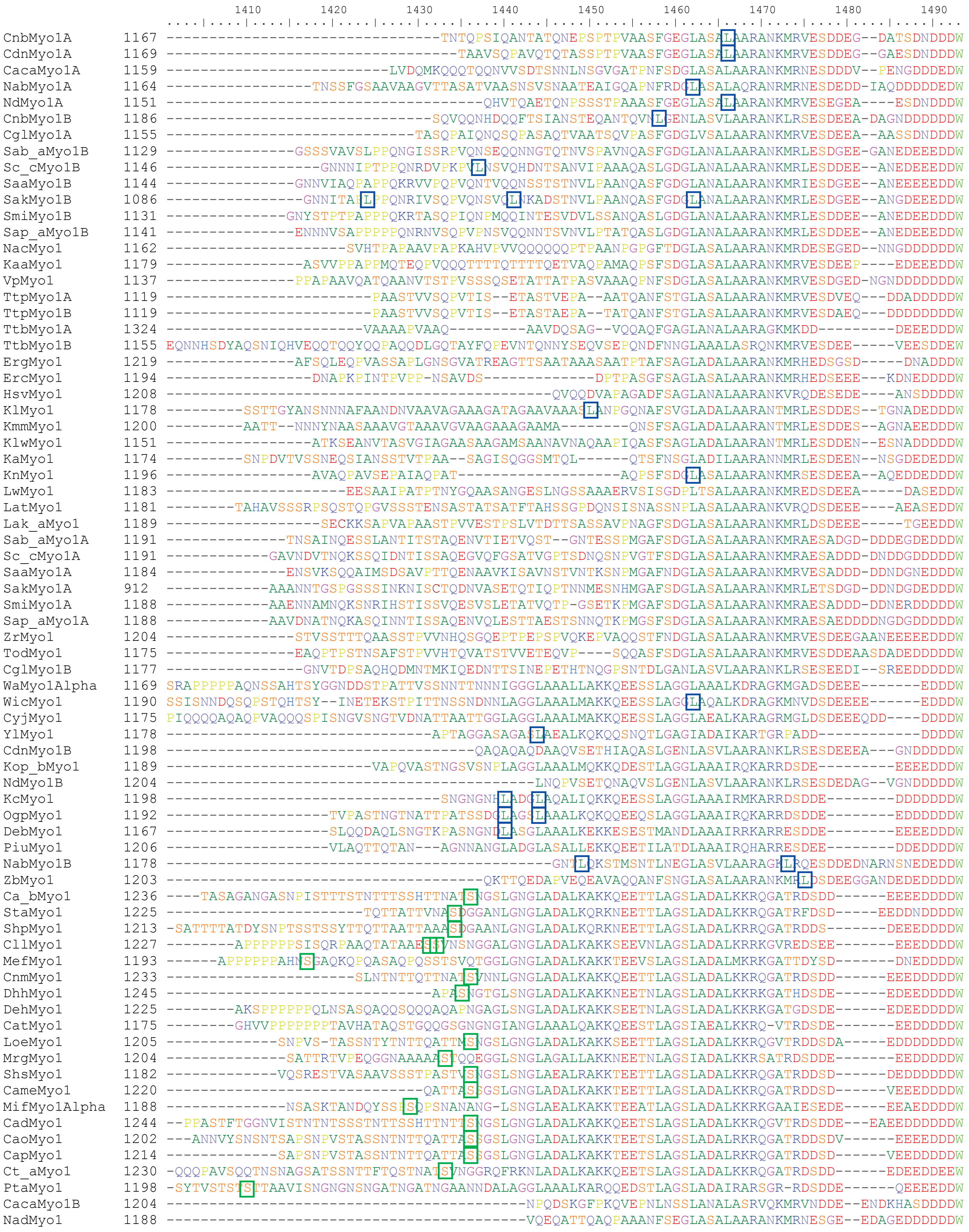

Supplement: Supplementary Data [file supp_evu152_suppl_data.zip › FigureS2.pdf]
