# Supplementary material for: Molecular Phylogeny of Sequenced Saccharomycetes Reveals Polyphyly of the Alternative Yeast Codon Usage
Source: Genome Biol Evol. 2014 Jul 22;6(12):3222–37. doi: 10.1093/gbe/evu152 (PMC4986446; doi:10.1093/gbe/evu152)
Supplement: Supplementary Data [file supp_evu152_suppl_data.zip › FigureS3.pdf]

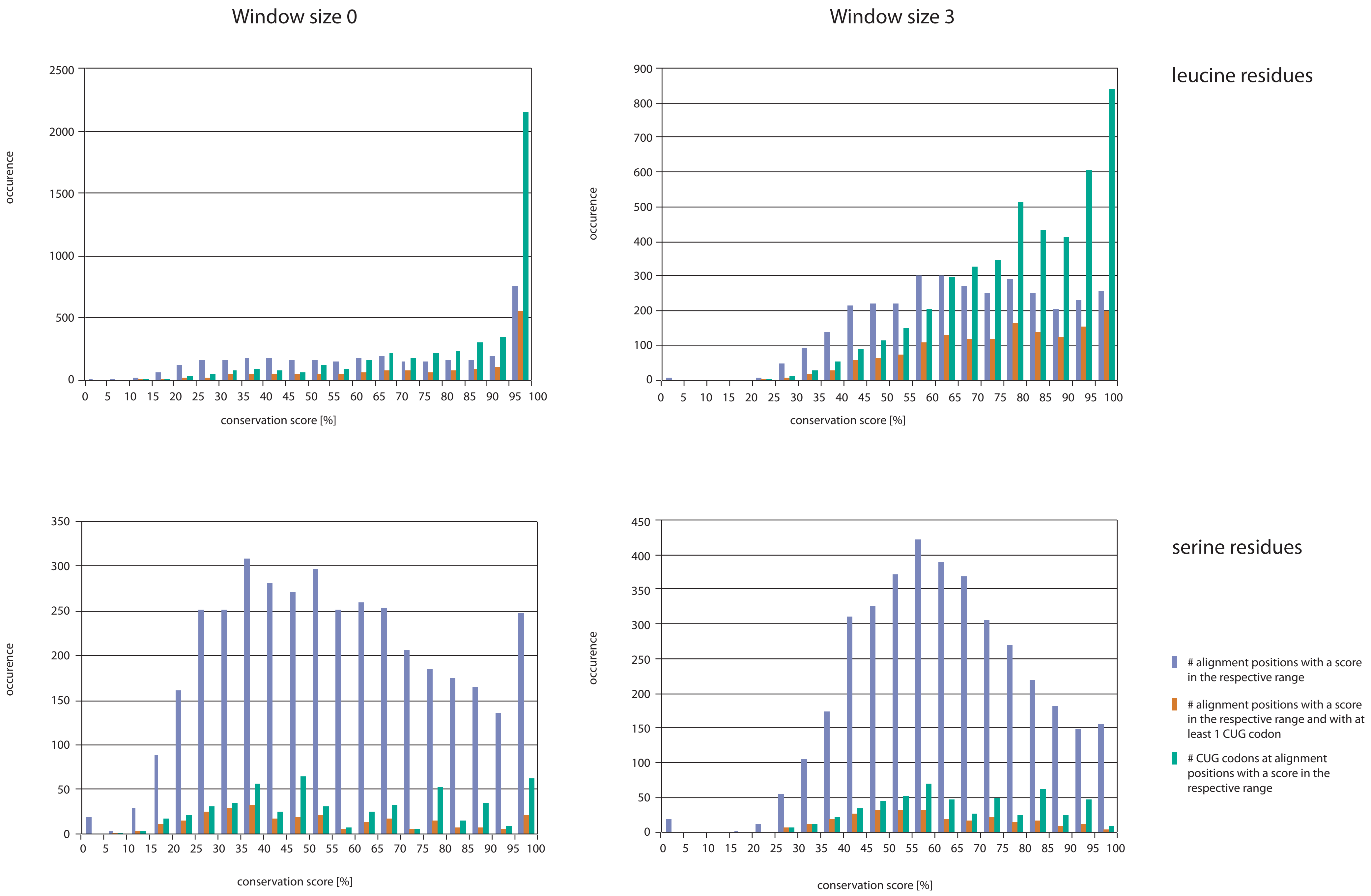
Window size 0
Window size 3
leucine residues
serine residues
occurence
conservation score [%]
2500
2000
1500
1000
500
0
900
800
700
600
500
400
300
200
100
450
400
350
300
250
200
150
100
50
350
0 5 10 15 20 25 30 35 40 45 50 55 60 65 70 75 80 85 90 95 100
alignment positions with a score in the respective range
alignment positions with a score in the respective range and with at least 1 CUG codon
CUG codons at alignment positions with a score in the respective range
