# Supplementary figures and images for: Molecular Phylogeny of Sequenced Saccharomycetes Reveals Polyphyly of the Alternative Yeast Codon Usage
Source: Genome Biol Evol. 2014 Jul 22;6(12):3222–37. doi: 10.1093/gbe/evu152 (PMC4986446; doi:10.1093/gbe/evu152)

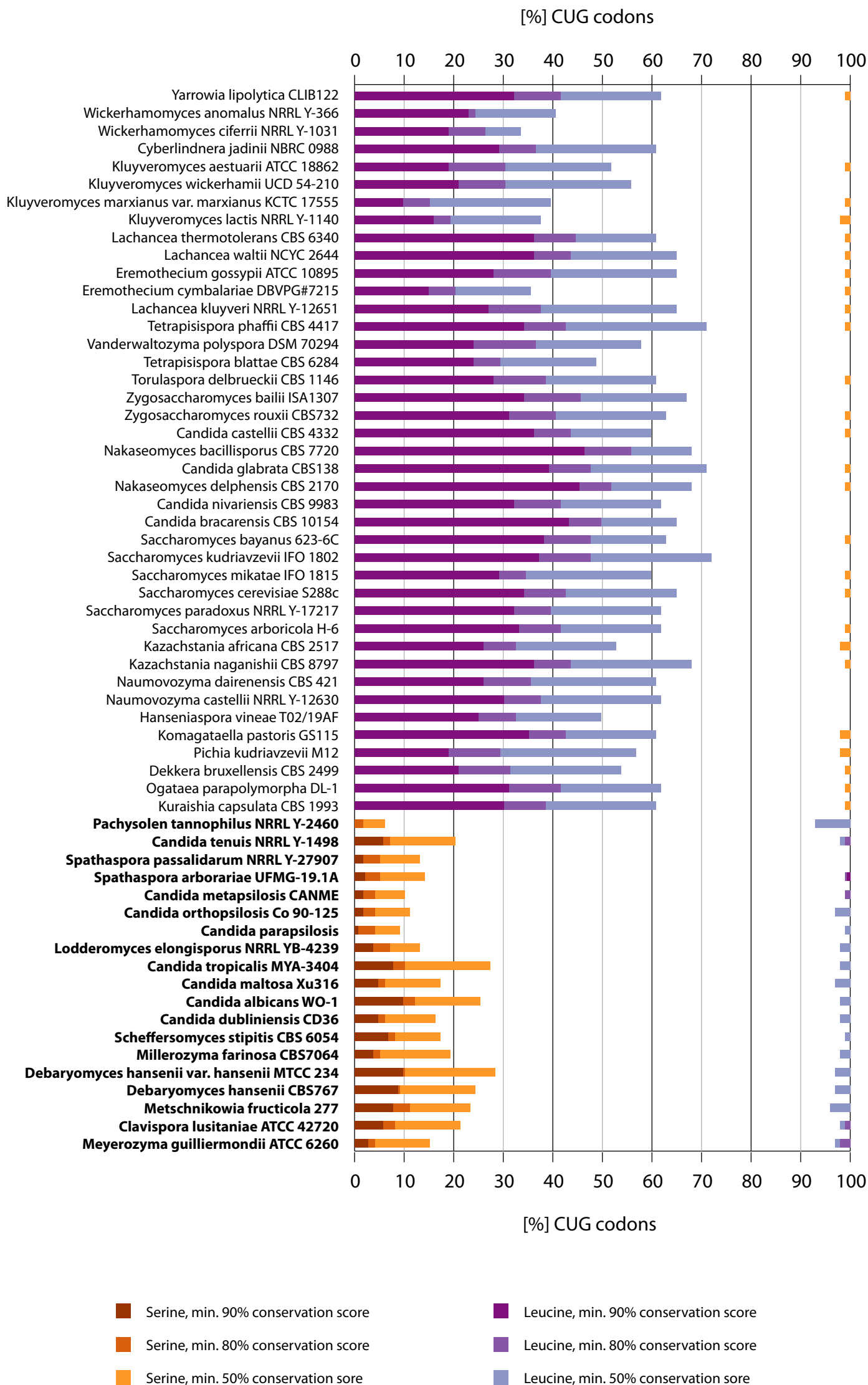

Supplement: Supplementary Data [file supp_evu152_suppl_data.zip › FigureS4.pdf]
